# New Chemical Scaffold with Antimicrobial Activity Identified in a Screening of Industrial Photoactive Compounds

**DOI:** 10.3390/antibiotics15030321

**Published:** 2026-03-20

**Authors:** José Manuel Ezquerra-Aznárez, Raquel Alonso-Román, Ainhoa Lucía, Raquel Andreu, Santiago Franco, José A. Aínsa, Santiago Ramón-García

**Affiliations:** 1 Department of Microbiology, Pediatrics, Radiology and Public Health, Faculty of Medicine, University of Zaragoza, 50009 Zaragoza, Spain; 2 Institute for Biocomputation and Physics of Complex Systems (BIFI), University of Zaragoza, 50018 Zaragoza, Spain; 3 Spanish Network for Research on Respiratory Diseases (CIBERES), Carlos III Health Institute, 28029 Madrid, Spain; 4 Department of Organic Chemistry, Faculty of Science, University of Zaragoza, 50009 Zaragoza, Spain; 5 Institute of Nanoscience and Materials of Aragon (INMA), Faculty of Science, CSIC-University of Zaragoza, 50009 Zaragoza, Spain; 6 Research and Development Agency of Aragon Foundation (Fundación ARAID), 50018 Zaragoza, Spain

**Keywords:** Gram-positive bacteria, new chemical scaffold, novel antimicrobials, photoactive compounds, *rny* locus

## Abstract

**Background/Objectives**: The emergence of antimicrobial resistance threatens advances achieved by medicine in the last century. This situation has been exacerbated by the suboptimal outcome of screening campaigns to provide novel antimicrobials. **Methods**: An alternative strategy was employed to identify new chemical scaffolds with antimicrobial activity. A collection of photoactive compounds originally synthesized for industrial purposes was screened for antibacterial activity. **Results**: 4*H*-pyran-4-ylidenes were identified as active against Gram-positive bacteria. Compounds belonging to this family displayed dose-dependent bactericidal activity against both wild-type and methicillin-resistant *Staphylococcus aureus*. No cytotoxicity was observed in the HepG2 hepatic cell line at the concentrations required for antimicrobial activity against *S. aureus*. Resistance to 4*H*-pyran-4-ylidenes in *S. aureus* was associated with point mutations in the *rny* locus, which encodes for a ribonuclease that plays a key role in RNA homeostasis. **Conclusions**: These findings indicate that chemical libraries not originally intended for drug discovery can be an innovative source of chemical diversity for the development of novel antimicrobials.

## 1. Introduction

The discovery of antimicrobials was one of the milestones of humankind in the 20th century. Their introduction brought unprecedented success in the treatment of infectious diseases and contributed to the development of other areas of medicine, such as invasive surgery and cancer chemotherapy. The current antimicrobial resistance (AMR) crisis threatens all these advances [1]. In 2021, there were an estimated 4.71 million deaths associated with AMR, including 1.14 million directly attributable to it, and it is projected that in 2050, at the current pace, there will be 1.91 million deaths directly attributable to AMR and 8.22 million deaths associated with it [2]. Therefore, there is an urgent need to discover and develop new antimicrobials.

Most of the currently available antimicrobial families were discovered in the mid-20th century, during a period known as the Golden Age of antimicrobial discovery. During that time, new antimicrobials were identified by screening soil-dwelling microorganisms for activity against pathogenic bacteria. This platform eventually collapsed when screening efforts repeatedly rediscovered known antibiotics or yielded molecules with significant toxicity issues [3]. The paradigm shifted at the end of the 20th century when the first bacterial genomes became available [4]. Genomic information allowed the identification of essential targets conserved across different bacterial species, and target-based approaches were developed to find active molecules against them. However, these approaches have had limited success in identifying new classes of antimicrobials [5,6], although notable exceptions exist. For example, the recently approved gepotidacin was discovered through screening of topoisomerase inhibitors [7,8]. Moreover, revisiting the Golden Age approach has yielded promising candidates such as teixobactin [9]. Continued discovery of new antimicrobial classes remains essential to ensure that the threat posed by AMR can be effectively managed.

Several factors can explain the failure of identifying new molecules through target-based approaches to inhibit microbial growth. First, the bacterial cell wall is a formidable barrier that protects bacteria from environmental threats, including antimicrobials [10]. Because of this, molecules that are good inhibitors of cytosolic targets might not be active against whole bacteria, thus limiting the success of target-based approaches in bacteria. An additional problem is the design of chemical libraries, which has been strongly influenced by Lipinski’s Rule of Five in order to improve the likelihood of the compounds having good oral bioavailability. Lipinski’s Rule of Five states that a potential drug should not violate more than one of these conditions: molecular weight under 500 Da, LogP below 5, fewer than five hydrogen bond donors, and fewer than 10 hydrogen bond acceptors. However, antimicrobials as a group represent an exception to these rules, displaying higher molecular weights and polarity [11]. Thus, chemical libraries used to run high-throughput screening (HTS) campaigns (either as target-based approaches or as whole cell screenings) for antimicrobial discovery have been conducted using libraries that did not fully reflect the chemical diversity of current antimicrobial families [12,13,14].

In this study, an unconventional approach for finding novel antimicrobials was taken, and unexplored chemical diversity was sought by screening a collection of photoactive organic dyes containing a 4*H*-pyranylidene moiety against a panel of pathogenic bacteria from the World Health Organization (WHO) priority list of antimicrobial-resistant pathogens [15]. Photoactive compounds are molecules capable of absorbing light and undergoing electronic excitation and charge-transfer processes, typically associated with extended π-conjugated systems. Organic dyes used in photochemical and optoelectronic applications encompass several well-known structural families, including xanthenes, phenothiazinums, triarylmethanes, and cyanines. In comparison with these classical dye scaffolds, 4*H*-pyran-4-ylidene derivatives also belong to the broader class of π-conjugated organic dyes but display a distinct structural framework and substitution pattern. The 4*H*-pyranylidene unit is a proaromatic electron donor that becomes aromatic upon charge transfer [16]. This enhanced aromatic stabilization strengthens its electron-donating capability, and its chemical structure can be readily modified through organic synthesis. Notably, representative compounds within this family comply with Lipinski’s Rule of Five, suggesting that this scaffold occupies a chemical region closer to drugs than purely dye-like compounds. For this reason, it is frequently employed as a key component in the development of π-conjugated systems across various fields, including dye-sensitized solar cells [17,18], second-order nonlinear optic applications [19], or photoinitiators for two-photon induced photopolymerization [20]. It was reasoned that the structural and electronic diversity of these photoactive molecular materials could provide access to unexplored chemical diversity, and it was found that 4*H*-pyran-4-ylidene derivatives were active against Gram-positive bacteria. Compounds belonging to this family were bactericidal to *Staphylococcus aureus*, including an MRSA strain, and non-toxic to mammalian cells.

## 2. Results

### 2.1. Identification of Antimicrobial Compounds in a Panel of Photoactive Molecular Materials

A single-dose activity assay was conducted using a set of 39 compounds of the 4*H*-pyran-4-ylidene family (see Table S1 for the chemical structure of Compounds **01**–**39**) from the photoactive molecular material (PMM) collection against a panel of relevant Gram-positive, Gram-negative, and mycobacterial strains (Table S2). Thirteen PMM compounds were active against at least one bacterial species at 50 µM (Tables S1 and S2).

**Figure 1:**
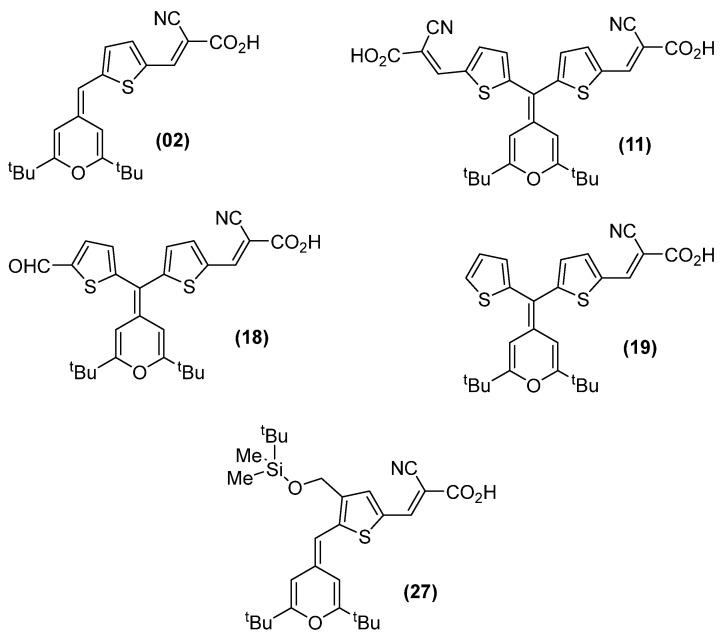
**Chemical structures of 4*H*-pyran-4-ylidene derivatives with broad-spectrum activity against Gram-positive species.** Compounds **02**, **11**, **18**, and **19** were reported in [21]; Compound **27** was reported in [22].

The activity of the 13 compounds identified as active against any of the bacterial strains tested was then validated using a dose–response assay, from which their Minimal Inhibitory Concentration (MIC) and Minimal Bactericidal Concentration (MBC) were found (Table 1). Four of the compounds selected from single-shot assay—**04**, **15**, **28**, and **36**—were inactive against all Gram-positive strains in the dose–response assays and, hence, were considered false-positives; these were discarded for further assays. The nine remaining compounds were validated as active and divided into two different groups according to their spectrum of activity. Five compounds—**02**, **11**, **18**, **19**, and **27**—were active against multiple Gram-positive species (Figure 1), and the other four—**05**, **07**, **13**, and **39**—were only active against one of the six Gram-positive strains tested (Table 1). MBC matched MIC values in most cases, strongly suggesting that 4*H*-pyran-4-ylidene derivatives were bactericidal to Gram-positive bacteria.

In order to test whether the lack of activity in Gram-negative bacteria was due to efflux, the susceptibility of *E. coli* strains lacking some of the major efflux systems to Compounds **02**, **18**, and **19** was evaluated. The MIC of these compounds was >50 µM against individual knockouts of the efflux pumps Smr, EmrE, MdrA and AcrAB-TolC as well as selected combinations (∆*emrE* ∆*mdrA*; ∆*emrE* ∆*acrB*; and ∆*emrE* ∆*mdrA* ∆*acrB*). Therefore, the deletion of these major efflux systems was insufficient to overcome *E. coli*’s intrinsic resistance to these compounds.

### 2.2. Compound **18** Is Bactericidal to S. aureus

**Figure 2:**
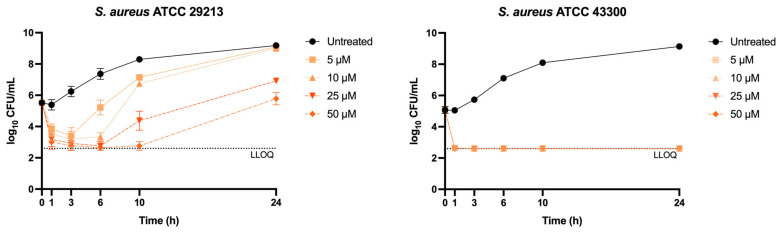
**Time-kill kinetics of Compound 18 against *Staphylococcus aureus*.** ATCC 29213 (MSSA, MIC = 25 µM) and ATCC 43300 (MRSA, MIC = 6.25 µM). Data represent the mean ± SD of three biological replicates. LLOQ (lower limit of quantification) was 400 CFU/mL.

In order to confirm the potential bactericidal activity of 4*H*-pyran-4-ylidene derivatives suggested by the dose–response MIC and MBC assays, time–kill kinetic assays (TKAs) of Compound **18** against two *S. aureus* strains were conducted: ATCC 29213 (methicillin-susceptible, MSSA, MIC = 25 µM) and ATCC 43300 (methicillin-resistant, MRSA, MIC = 6.25 µM) were conducted. Indeed, Compound **18** exhibited bactericidal activity against both strains, achieving maximal killing at the 6 h timepoint for concentrations equal to or greater than the MIC for the MSSA strain (Figure 2). Subinhibitory concentrations were also able to reduce the bacterial population at earlier timepoints, but growth soon restarted. On the other hand, Compound **18** was more potent against the MRSA strain, which was rapidly killed at all concentrations tested.

In order to evaluate whether the MSSA outgrowth was caused by the emergence of stable genetic resistance or transient phenotypic adaptations, the population exposed to 25 µM Compound **18** was sub-cultured in Compound **18**-free media and subsequently tested its susceptibility to Compound **18**. MIC determination using standard broth microdilution showed an increase in the MIC from 25 µM to 100 µM, indicating the emergence of a subpopulation with decreased susceptibility to Compound **18**.

### 2.3. Mutations in the rny Locus Confer S. aureus Resistance to Compound **18**

**Figure 3:**
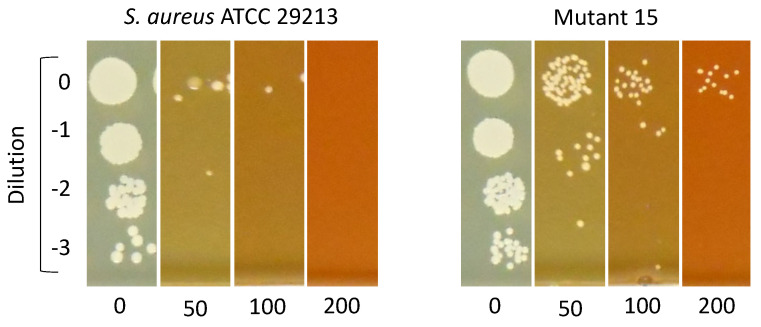
**Drop-dilution Compound 18 susceptibility testing of *S. aureus* ATCC 29213 and one of the resistant mutants isolated (mutant 15).** Compound **18** concentrations are indicated in µM (horizontally).

*S. aureus* MSSA mutants resistant to Compound **18** were isolated from two independent experiments. First, cells were treated with 25 µM (1× MIC) of Compound **18** for 24 h and, subsequently, exposed to 100 µM (4× MIC) of Compound **18** for a further 24 h. Then, the culture was inoculated onto compound-free plates, and individual clones were selected for MIC assays. Second, mutants were selected directly by seeding a culture onto plates containing 100 µM of Compound **18**. The frequency of mutation of *S. aureus* MSSA to Compound **18** was 7·10^−7^. In both cases, mutants with MIC increases of 4-fold or higher (i.e., 100 µM) were isolated. Their resistant phenotype was then validated using a drop-dilution assay to confirm that the mutants were able to grow in the presence of up to 200 µM of Compound **18** (Figure 3 and Figure S1). In contrast, multiple independent attempts to isolate resistant mutants in the MRSA background did not yield clones with stable MIC increases. Although colonies were recovered under selection, none displayed changes in their MIC upon retesting.

The genome of 17 mutants—15 isolated from liquid cultures and 2 from agar plates—that were able to grow in the presence of 50–200 µM of Compound **18** was sequenced. Those mutations identified, which were selected in two independent experiments, suggest a potential role of RNase Y in the susceptibility of *S. aureus* to Compound **18** (Table S4). Therefore, two nonsynonymous mutations (P280L and G240D) in the *rny* locus were genetically validated. Both engineered mutants displayed an increased MIC to Compound **18** compared to the engineered strain in which the wild-type allele was replaced by itself, confirming its role in *S. aureus* susceptibility (Table 2).

**Figure 4:**
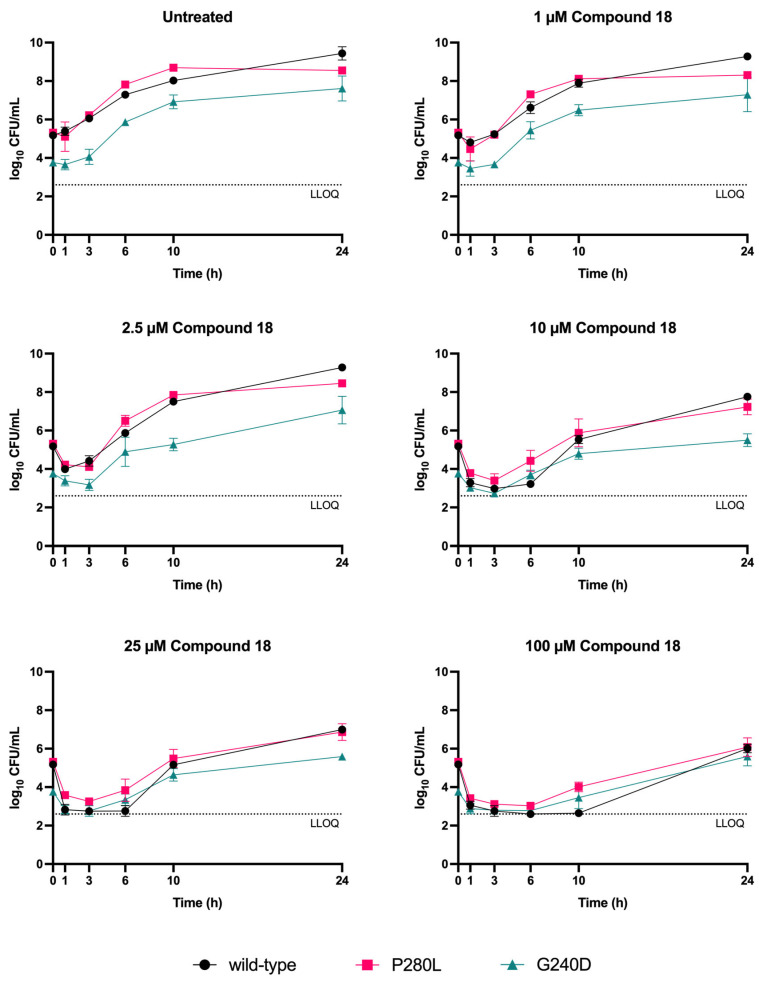
**Time-kill kinetics of Compound 18 against *S. aureus rny* engineered point mutants.** Wild-type *S. aureus* ATCC 29213 was engineered to replace wild-type *rny* with alleles carrying the P280L or G240D mutation and itself. Data represent the mean ± SD of three biological replicates. LLOQ (lower limit of quantification) was 400 CFU/mL.

These results were subsequently validated by TKA. Surprisingly, it was found that the killing kinetics of the engineered mutants resembled that of the strain engineered with the wild-type allele, with a rapid phase of initial killing followed by a rebound. However, the proportion of the population that survived the killing was higher in the mutant strains compared to the wild-type control, leading to a quicker recovery after the initial killing (Figure 4). It was also observed that the G240D mutation caused growth defects during the early phase of growth.

### 2.4. 4H-Pyran-4-ylidene Derivatives Are Not Cytotoxic to HepG2 Cells

The cytotoxicity of compounds **02**, **11**, **18**, **19**, and **27** against the HepG2 cell line was determined using the neutral red assay. All five compounds were not cytotoxic at the concentrations at which antimicrobial activity was observed, with selectivity indices ranging between 4.16 and 74.26 (Table 3).

### 2.5. Structure–Activity Relationships Studies

**Figure 5:**
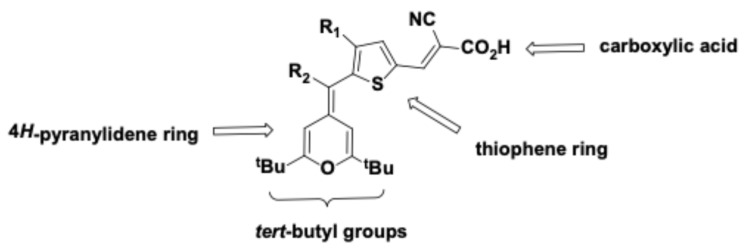
**Pharmacophore of 4*H*-pyran-4-ylidenes.** The presence of *tert-*butyl substituents, pyranylidene and thiophene rings, and a carboxyl group are required for antimicrobial activity against Gram-positive bacteria.

After comparing data gathered from the dose–response assays with the structure of the compounds (Table S1), some features related to their antimicrobial activity were observed. Based on these preliminary observations, a small set of additional derivatives (Compounds **40**–**47**; Table S1) was designed and synthesized to probe the structural requirements of the scaffold: (i) the role of the *tert*-butyl substituents, (ii) the presence of the carboxyl group, and (iii) the influence of different heterocyclic or bulky substituents on antimicrobial activity. After testing these compounds against Gram-positive strains (Table 4), some essential features to the 4*H*-pyran-4-ylidenes’ activity were inferred. First, *tert-*butyl substituents in the 4*H*-pyran-4-ylidene ring appear to be necessary for antimicrobial activity, as their replacement by phenyl groups rendered compounds inactive. Second, carboxyl groups and the presence of a five-member heterocycle were essential for activity against Gram-positive bacteria; when removed, activity was also lost. Bulky substituents, on the other hand, were not critical for antimicrobial activity (Figure 5). However, given the small number of molecules tested, their specific contributions to potency remain to be defined.

Based on the structure–activity relationship (SAR) trends observed across this series, a preliminary pharmacophore was proposed comprising the 4*H*-pyran-4-ylidene ring, two *tert*-butyl substituents, a five-membered heterocycle positioned to preserve conjugation, and a carboxyl group. Disruption of any of these features was associated with reduced potency, suggesting that both electronic and steric contributions from these groups are critical for antibacterial activity.

## 3. Discussion

Only six new classes of antibacterial compounds have been introduced in the last five decades. This is in stark contrast to the prolific decades of the Golden Era of antibiotic discovery. The paucity of novel antimicrobials illustrates the limited success of efforts after the Waksman platform [23]. The latest WHO report on antibacterial agents in clinical and preclinical development highlighted that most of the clinical candidates belong to pre-existing classes, underscoring the need for new antimicrobial families [24]. Simultaneously, bacterial strains have emerged resistant to virtually every antimicrobial, with mortality rates close to the pre-antibiotic era [25].

To address the growing AMR crisis, multiple innovative approaches have attempted to tackle this stagnation. These include screening under conditions that mimic the in vivo environment [26], designing chemical libraries enriched for properties that enhance bacterial penetration [13,27], and leveraging advances in culturing techniques to access previously unculturable microbes. The development of in situ culture systems and sequencing and bioinformatics tools has yielded new antimicrobial candidates with novel mechanisms of action, such as teixobactin and darobactin [9,28]. More recently, artificial intelligence and machine learning have emerged as powerful tools for accelerating antimicrobial discovery, identifying both small molecules and antimicrobial peptides with promising activity [29,30].

In this study, a different approach which involved seeking novel antimicrobial scaffolds from an unconventional chemical space was used. Rather than focusing on molecules designed for biological activity, we screened the in-house PMM (Photoactive Molecular Materials) collection of compounds, originally designed to be part of solar cells. This screening led to the identification of a novel family, the 4*H*-pyran-4-ylidenes, which exhibits activity against Gram-positive bacteria. Five of these compounds were active against multiple species, with MIC and MBC values ranging from 1.56 to 25 µM (Table 1). A structural similarity search using the SwissSimilarity tool [31] against the ZINC database revealed no analogous compounds, suggesting that this is a chemically distinct scaffold that could offer a new path for antimicrobial discovery. Given the absence of detectable activity against both wild-type *E. coli* and efflux deficient mutants, the present study prioritized characterization in Gram-positive pathogens. Notably, Compound **18** exhibited a lower MIC against MRSA than MSSA and did not give rise to resistant mutants in the MRSA background. The difference in potency may arise from differences in cell envelope organization or physiological adaptations associated with methicillin resistance, which could enhance penetration of Compound **18**, although further work is needed to clarify the underlying mechanisms.

Mutations in the *rny* locus, encoding the RNase Y enzyme, conferred decreased susceptibility of *S. aureus* to Compound **18**, one of the five 4*H*-pyran-4-ylidenes with activity against multiple Gram-positive bacterial species (Table 2). In Gram-positive bacteria, RNaseY plays a central role in RNA homeostasis, contributing to mRNA degradation and therefore, overall RNA stability. This effect is achieved in coordination with other RNases, which together form the degradosome [32]. RNase Y is not essential in *S. aureus*, but its deletion stabilizes several tens of mRNAs and decreases virulence, highlighting the broad impact of its deletion in gene expression regulation [33]. Whole genome sequencing of independently selected resistant mutants revealed recurrent mutations in *rny*. Importantly, introducing these mutations in the wild-type background resulted in increased MIC. Notably, rather than complete resistance, mutations in *rny* appear to increase the fraction of *S. aureus* surviving initial drug exposure, suggesting a mechanism of tolerance rather than strict resistance (Figure 4). It is, therefore, proposed that these mutations partially impair RNase Y activity and, consequently, change the stability of multiple mRNAs, increasing the fraction of bacteria able to endure Compound **18** exposure. A conceptually similar drug tolerance mechanism has previously been described in *Mycobacterium tuberculosis*, where the loss of RNase J—homologous to the RNase J1 and RNase J2 enzymes of the Gram-positive degradosome [34]—enhances survival in the presence of multiple anti-TB drugs [35]. Given that RNase Y is absent in Gammaproteobacteria (the class including all Gram-negative strains tested in this study) and mycobacteria and it is non-essential in *S. aureus* [36], it is unlikely to be the direct target of 4*H*-pyran-4-ylidenes but rather involved in its mechanism of resistance. Future work can focus on transcriptomic and proteomic analyses to evaluate *S. aureus* response to 4*H*-pyran-4-ylidenes.

Two main hypotheses can explain the lack of activity of 4*H*-pyran-4-ylidenes against the Gram-negative and mycobacterial strains tested: (i) the target of these compounds is unique to Gram-positive bacteria; and (ii) the differences in cell envelope architecture prevent intracellular accumulation of 4*H*-pyran-4-ylidenes. Regarding the second hypothesis, 4*H*-pyran-4-ylidenes were not active against *E. coli* strains lacking major efflux systems (AcrAB-TolC, EmrE, MdrA and combinations thereof) in standard broth microdilution assays. However, the possibility of other efflux systems contributing to their lack of activity cannot be excluded.

Preliminary toxicity studies in the HepG2 cell line provided selectivity indices between 4.16 and 74.26 (Table 3), indicating preferential antibacterial activity over mammalian cytotoxicity. While there is not a clear universal threshold to define a compound as safe, selective indices above 10 are considered favorable in early drug development [37]. This value was generally achieved for all compounds and bacterial strains tested, including methicillin-resistant *S. aureus* but not for methicillin-susceptible *S. aureus*.

SAR studies showed that three substituents are required for the antibacterial activity of the 4*H*-pyran-4-ylidene family: (1) a *tert*-butyl group, (2) carboxyl groups, and (3) a five-membered heterocycle (Figure 5). While these elements appear necessary for activity in the current dataset, the limited chemical space explored precludes definitive conclusions regarding their mechanistic roles or contribution to overall potency. Notably, this structural framework is distinct from the current antimicrobials in use against Gram-positive infections, such as β-lactams, glycopeptides, lipopeptides, or oxazolidinones, highlighting the novelty of this scaffold. Such structural divergence may represent an advantage in the context of antimicrobial resistance, as cross resistance with existing drug classes may be less likely. From a medicinal chemistry perspective, several of these features may represent structural alerts and liabilities for further development. *Tert-*butyl groups are usually susceptible to cytochrome P450 oxidation, potentially reducing metabolic stability of the compounds in which they are present, although this feature is not a limiting factor [38]. Carboxylic acid groups are also associated with poor pharmacological properties due to the limited membrane permeability of charged molecules, metabolic instability, and toxicity arising from the chemical reactivity of their degradation products; these issues are usually solved by replacing them with bioisosteres with better pharmacological properties [39]. Finally, the thiophene moiety is viewed as a double-edged sword, providing a versatile scaffold; however, it is also susceptible to cytochrome P450 metabolism, which can lead to the formation of toxic metabolites [40]. Compared with current antibacterial drugs, which have undergone extensive characterization for chemical use, the present scaffold remains at an early stage. While its potential liabilities are well recognized, future work could include the design of analogs to mitigate them; however, the development of a 4*H*-pyran-4-ylidene lead candidate was beyond the scope of this work, which is exploratory in nature. A better understanding of the mechanism of action will also help refine the SAR, leading to optimized derivatives with potency and selectivity comparable to current therapies used to treat *S. aureus* infections.

*S. aureus* remains a major global health concern, causing an array of diseases that range from superficial skin infections to fatal bacteriemia. MRSA is currently one of the most prevalent antimicrobial-resistant pathogens and is classified as a high-priority target for antimicrobial development [15]. The main current therapeutic options for MRSA are vancomycin, daptomycin, and linezolid, although the emergence of vancomycin-intermediate and -resistant strains threatens its future availability as an option [41]. Given the scarcity of new antibacterials in the development pipeline, a novel approach exploring chemical diversity originally intended for non-biological applications could represent an untapped source of innovation. While further investigation is needed to evaluate the therapeutic potential of 4*H*-pyran-4-ylidenes, these findings support the original hypothesis that expanding screening efforts beyond traditional drug-like molecules can lead to the identification of novel antimicrobial scaffolds.

## 4. Materials and Methods

### 4.1. Bacterial Strains and Culture Conditions

Bacterial strains used in this study are listed in Table S2. Gram-positive and Gram-negative bacteria were propagated in Müller–Hinton broth (Panreac AppliChem, Barcelona, Spain) with 22 mg/L Ca^2+^ and 12 mg/L Mg^2+^ (Müller–Hinton II). *Corynebacterium diphtheriae* was grown in Brain Heart Infusion (BHI) broth (Difco, Sparks, MD, USA). Mycobacterial strains were propagated in Middlebrook 7H9 broth (Difco) supplemented with 10% (*v*/*v*) Middlebrook ADC (Difco) and 0.05% Tween 80 (Scharlab, Barcelona, Spain). For CFU enumeration, Gram-positive bacteria were seeded onto LB agar (10 g/L tryptone, 5 g/L yeast extract, 5 g/L NaCl, and 17 g/L agar).

### 4.2. Compounds

Compounds belonging to the 4*H*-pyran-4-ylidene family were synthesized and characterized in-house following the schemes described in the Supplementary Materials or previously published studies. Compounds were dissolved in DMSO at a final concentration of 4 mM and stored at −20 °C. Secondary stocks for single-shot and dose–response assays were prepared in 96-well V-bottomed plates at 40-fold the final concentration in the assay plate. For subsequent assays, Compounds **02**, **11**, **18**, **19**, and **27** were dissolved in DMSO at 10 mM.

### 4.3. Screening of the Photoactive Molecular Material (PMM) Compound Library

Antimicrobial activity was evaluated following a two-step process. First, a single-shot assay was performed with compounds being tested at a final concentration of 50 µM against strains described in Table S2. Compounds active at 50 µM were subsequently tested in dose–response assays in two-fold serial dilutions of the compounds, starting from 50 µM. To perform each assay, compounds were prepared in plates at 40-fold the final concentration. Then, 5 µL was transferred onto 96-well flat-bottomed plates containing 195 µL of the corresponding bacterial suspension at a final density of 10^5^ CFU/mL. Growth controls were treated with the equivalent volume of DMSO (2.5% final concentration). Plates were incubated for 24–144 h depending on the bacterial species (Table S2) before the addition of 30 µL of a solution containing Tween 80 (10%, *v*/*v*) and MTT [3-(4,5-dimethylthiazol-2-yl)-2,5-diphenyltetrazolium bromide] (2.5 mg/mL) (Sigma, Madrid, Spain) as a reporter of bacterial growth. Optical density measures at 580 nm after 1 h incubation (overnight for *M. tuberculosis*) were used to define the Minimal Inhibitory Concentration (MIC), which was the lowest compound concentration that inhibited 90% MTT conversion to formazan. To minimize potential optical interference from the test compounds in the single-shot assay, compound activity was also evaluated using an agar-based readout. Briefly, five microliters of each well was seeded onto agar plates and incubated for an additional 24–72 h. Compounds were considered active if neither bacterial growth nor resazurin reduction was observed on the agar surface.

For *S. aureus* MSSA and MRSA dose–response assays, performance was validated by MIC determinations for reference antibiotics oxacillin (0.12 µg/mL and 4 µg/mL, respectively; the latter is consistent with the breakpoints defined for resistance) and rifampicin (0.006 µg/mL), which matched the values reported in CLSI M100 [42]. 

In order to determine the Minimal Bactericidal Concentration (MBC), before the addition of the MTT solution in a conventional MIC assay, 5 µL of each well was transferred to 96-well plates containing LB-agar before the addition of the MTT solution and incubated for a further 24 h. The readout was performed after the addition of 30 µL of a 0.1 mg/mL resazurin solution (Sigma) to each well and color changes from blue to pink were visually evaluated.

### 4.4. Time-Kill Kinetic Assays

A starting inoculum of 10^5^ CFU/mL of *S. aureus* was treated with 50, 25, 10, 5 µM of Compound **18** (corresponding to 2-, 1-, 0.4-, and 0.2-fold the MIC against *S. aureus* ATCC 29213). The growth control was treated with the concentration of DMSO corresponding to the amount present in the 50 µM culture (0.5% final concentration). Then, 10-fold serial dilutions in PBS were seeded onto LB agar plates at 0, 1, 3, 6, and 24 h, and CFUs enumerated after 24 h incubation at 37 °C. Following exposure to 25 µM of Compound **18**, MSSA cells were diluted 1:1000 in fresh medium and incubated overnight. Following this passage, Compound **18** MIC was determined using broth microdilution as described above. Each biological duplicate was tested in technical duplicates. The parental strain was included in parallel as a control.

For TKA performed with the engineered *S. aureus* strains, Compound **18** was tested at 100, 25, 10, 2.5, and 1 µM. The corresponding growth controls were treated with 1% DMSO, which represented the highest solvent concentration present in the assay, to account for potential solvent effects.

### 4.5. Mutant Isolation Assays

*S. aureus* ATCC 29213 cultures were treated with 25 µM of Compound **18** for 24 h and subsequently treated with 100 µM of Compound **18** and incubated overnight before seeding 10-fold serial dilutions onto drug-free LB agar plates. Twenty-three colonies were selected for subsequent phenotypic validation.

Mutant isolation was also attempted by seeding 10^7^, 10^8^, and 10^9^ CFUs of *S. aureus* ATCC 29213 onto Müller–Hinton II agar plates with 100 µM of Compound **18**. Plates were incubated at 37 °C for 48 h to allow a selection of late-growing mutants. Eleven colonies were selected for phenotypic validation. The frequency of mutation was calculated as the ratio of resistant colonies to the total number of viable CFU plated.

In both cases, the obtained colonies were cultured in Müller–Hinton II broth and used to determine the MIC of Compound **18** against them. Colonies with a 4-fold or greater increase in their MICs compared to the reference strain were selected for secondary validation, which consisted of seeding 5 µL of 10-fold serial dilutions on Müller–Hinton agar plates containing 50, 100, or 200 µM of Compound **18**.

### 4.6. Genomic DNA Extraction

Mutants of *S. aureus* with decreased susceptibility to Compound **18** were grown overnight in tubes containing 5 mL of Müller–Hinton II broth. Bacteria were then collected by centrifugation and resuspended in 400 µL of TE buffer (10 mM Tris-HCl, 1 mM EDTA). Lysostaphin (Sigma) was then added at a final concentration of 0.1 mg/mL, and the mixture was incubated for one hour at 37 °C. Then 0.05 mg of proteinase K dissolved in 75 µL of a 10% sodium dodecyl sulfate solution were added and samples were incubated for 10 min at 65 °C. Genomic DNA extraction was performed by adding 750 µL of chloroform–isoamyl alcohol (24:1, *v*/*v*). Samples were mixed thoroughly before centrifugation (5 min, 13,500 RCF). The supernatant was transferred to tubes containing 420 µL of ice-cold isopropanol. DNA precipitation was carried out at −20 °C for two hours before centrifuging samples (5 min, 13,500 RCF) to collect the nucleic acids. Pellets were finally resuspended in 50 µL of nuclease-free water (Qiagen, Hilden, Germany). DNA was quantified by absorbance readings at 260 nm using an ND-1000 spectrophotometer (NanoDrop, Thermo Fisher, Waltham, MA, USA).

### 4.7. Whole Genome Sequencing

Genomic DNA of seventeen mutants and the parental *S. aureus* ATCC 29213 was sequenced at the FISABIO Sequencing and Bioinformatics Service (Valencia, Spain) using Illumina technology or at the CIBA genomics facility (Zaragoza, Spain) using IonTorrent technology. Reads were filtered with fastp [43] to remove low-quality bases at the 3′ end. The filtered reads were subsequently mapped to the *S. aureus* ATCC 29213 chromosome (available at https://genomes.atcc.org/genomes/21eea9803c88405a, accessed on 16 March 2026) with BWA [44], and potential duplicates were removed with Picard tools (http://broadinstitute.github.io/picard, accessed on 16 March 2026). Single-nucleotide polymorphisms (SNPs) were identified using VarScan [45]; if at least 20 reads supported the genomic position, the SNP was found at a frequency of 0.9 or higher and was not found near an indel region (10 bp). Indels were identified using Genome Analysis ToolKit (GATK) [46]. SNPs and indels were annotated using SnpEFF [47], and those common to the parental strain were later removed.

### 4.8. Genetic Validation of Mutations Associated with Compound **18** Resistance

Mutations identified in Compound **18**-resistant *S. aureus* mutants were validated using the pMAD vector [48] to perform allelic replacement in the parental strain. Mutant alleles were amplified by PCR using genomic DNA as the template and cloned between the *Xma*I and *Sfo*I restriction sites of pMAD. The resulting plasmids were then electroporated into the methylase-deficient *Escherichia coli* GM2929 to obtain plasmid DNA suitable for electroporation into *S. aureus*. One microgram of each plasmid was electroporated into electrocompetent *S. aureus* and transformants were selected on Tryptic Soy Agar (TSA, Difco) plates containing 2.5 µg/mL erythromycin and 80 µg/mL X-gal (5-Bromo-4-Chloro-3-Indolyl β-D-Galactopyranoside) after 72 h of incubation at 28 °C. Then, one cyan colony from each transformation was transferred to Tryptic Soy Broth (TSB, Difco) with 2.5 µg/mL erythromycin and incubated at 44 °C overnight. This culture was inoculated onto TSA plates containing erythromycin and X-gal at 44 °C to select colonies in which the recombinant pMAD derivative was integrated into the chromosome of *S. aureus*. Such cyan colonies were replicated onto TSA plates with erythromycin and X-gal and cultured in TSB without erythromycin overnight at 28 °C; once grown, they were 10-fold serially diluted and seeded onto erythromycin-free TSA plates with X-gal and incubated at 37 °C for 48 h; white colonies (indicative of the loss of integrated pMAD derivative) were screened by colony PCR followed by Sanger sequencing to confirm the presence of the mutation.

### 4.9. Cytotoxicity Assays

Cytotoxicity of compounds **02**, **11**, **18**, **19**, and **27** (from a 10 mM DMSO stock solution) was tested in HepG2 cells (ECACC 85011430), which were exposed to a concentration of 250 µM and to a series of two-fold dilutions from 100 to 1.56 µM. Toxicity was determined using the Neutral Red Uptake (NRU) assay as described in ISO 10993-5:2009(E) [49]. Cells were seeded in 96-well flat-bottomed plates at a density of 2.5·10^4^ cells/well and incubated for 24 h in Dulbecco’s Modified Eagle Medium (DMEM). Then, the culture medium was replaced with 100 µL of fresh DMEM with the compounds to be tested. The assay was performed in technical triplicates, including 2.5% DMSO (final concentration of DMSO in the 250 µM wells), 10% DMSO (death control), and 1 µg/mL rifampicin (positive control).

After incubation with the compounds for 24 h, the culture medium was replaced by neutral red-containing medium. Cells were subsequently incubated for 3 h before the desorb solution (1% glacial acetic acid, 50% ethanol, *v*/*v*) was added and optical density was measured at 540 nm. Compounds were also tested under the same conditions in plates without cells in order to ensure that their strong colors did not interfere with the readout method. In the absence of cells, compounds alone showed negligible interference with the assay readout, as the compound-containing medium was replaced with neutral red-containing medium.

## Figures and Tables

**Table 1:** **Minimal inhibitory and bactericidal concentrations for the nine compounds tested in dose–response assays.** Concentrations are expressed in µM. MIC and MBC values ≤ 50 µM are color-coded: the darkest shade of blue represents the lowest values (≤1.56 µM), the intermediate shade represents 3.125–6.25 µM, and the lightest shade represents the highest values (12.5–50 µM). MICs and MBCs were determined in two independent experiments using technical duplicates. MSSA: Methicillin-susceptible *Staphylococcus aureus.*

	*C. diphtheriae*		*C. glutamicum*		*E. faecalis*		MSSA		*S. epidermidis*		*S. agalactiae*	
Compound	MIC	MBC	MIC	MBC	MIC	MBC	MIC	MBC	MIC	MBC	MIC	MBC
**02**	6.25	6.25	6.25	6.25	25	25	25	25	12.5	12.5	12.5	12.5
**05**	12.5	>50	>50	>50	>50	>50	>50	>50	>50	>50	>50	>50
**07**	>50	>50	>50	>50	>50	>50	25–50	>50	>50	>50	>50	>50
**11**	12.5	12.5	25	25	50	50	50	>50	12.5	12.5	12.5	12.5
**13**	25–50	25–50	>50	>50	>50	>50	>50	>50	>50	>50	>50	>50
**18**	1.56	1.56	6.25	6.25	1.56	3.12	25	25	3.12	3.12	6.25	6.25
**19**	0.78	1.56	3.12	3.12	0.78	0.78	>50	>50	>50	>50	3.12	3.12
**27**	1.56	1.56	6.25	6.25	3.12	3.12	>50	>50	>50	>50	1.56	1.56
**39**	1.56	1.56–6.25	>50	>50	>50	>50	>50	>50	>50	>50	>50	>50

**Table 2:** **Antimicrobial activity of Compound 18 against genetically engineered *S. aureus* ATCC 29213 *rny* mutants.** MICs were determined in two independent experiments using technical duplicates.

Strain	MIC Fold Change Relative to Wild-Type
*S. aureus* ATCC 29213 Rny WT	1
*S. aureus* ATCC 29213 Rny P280L	8
*S. aureus* ATCC 29213 Rny G240D	4

**Table 3:** **Cytotoxicity of 4*H*-pyran-4-ylidene derivatives against HepG2 cells and selectivity indices** (calculated as the ratio CC_50_/MIC_90_). ND: not determined, due to the lack of activity of the compounds against specific strains; CC: cytotoxic concentration. Selectivity indices above 10 are shown in blue. CCs were determined in two independent experiments with technical triplicates.

	CC_50_ and CC_90_ (µM) of Compound				
Compound	02	11	18	19	27
CC_50_	167	370	57	58	26
CC_90_	250	>250	100	100	50
**Strain**	**Selectivity index (CC_50_/MIC_90_)**				
*S. aureus* (MSSA)	6.68	7.4	4.56	ND	ND
*S. epidermidis*	13.36	29.6	18.24	ND	ND
*S. agalactiae*	13.36	29.6	9.12	18.56	16.64
*E. faecalis*	6.68	7.4	36.48	74.26	8.32
*C. glutamicum*	26.72	14.8	9.12	18.56	4.16
*C. diphtheriae*	26.72	29.6	36.48	74.26	16.64

**Table 4:** **Antimicrobial activity of Compounds 40–47.** Concentrations are expressed in µM. MIC and MBC values ≤ 50 µM are color-coded: the darkest shade of blue represents the lowest values (6.25 µM), and the lightest shade represents the highest values (12.5–50 µM). MICs and MBCs were determined in two independent experiments using technical duplicates. MSSA: Methicillin-susceptible *Staphylococcus aureus*.

	*C. diphtheriae*		*C. glutamicum*		*E. faecalis*		MSSA		*S. epidermidis*		*S. agalactiae*	
Compound	MIC	MBC	MIC	MBC	MIC	MBC	MIC	MBC	MIC	MBC	MIC	MBC
**40**	>50	>50	>50	>50	>50	>50	>50	>50	>50	>50	>50	>50
**41**	>50	>50	>50	>50	>50	>50	>50	>50	>50	>50	>50	>50
**42**	12.5	25	>50	>50	>50	>50	>50	>50	>50	>50	>50	>50
**43**	12.5	12.5	12.5	12.5	25	50	50	>50	12.5	25	12.5	12.5
**44**	6.25	6.25	12.5	12.5	50	50	50	50	25–50	25–50	25	25
**45**	12.5	12.5	25	25	25	25	25	50	12.5	12.5	12.5	12.5
**46**	50	50	>50	>50	>50	>50	>50	>50	>50	>50	25	25
**47**	12.5	12.5	12.5	12.5	25	50	50	50	12.5	25	25	25

## Data Availability

WGS data were submitted to SRA under accession number PRJNA1240371. All other relevant data are available upon request.

## Supplementary Materials

The following supporting information can be downloaded at https://www.mdpi.com/article/10.3390/antibiotics15030321/s1. Figure S1: Drop-dilution Compound **18** susceptibility testing of nineteen *S. aureus* mutants.; Table S1: Bacterial strains used in the screening of the photoactive molecular material compound collection; Table S2: Single-shot assay of the 4*H*-pyran-4-ylidene derivatives; Table S3: Summary of 4*H*-pyran-4-ylidene derivatives used in this study; Table S4: Mutations identified in *S. aureus* resistant mutants. Table S5. Absorbance of the compounds used in this study relative to the positive growth control for each strain. Table S6. UV spectra of the compounds used in this study. Figures S2–S70: Spectral Characterization Data For New Synthesized Compounds. Refs [17,21,22,50,51,52,53,54,55,56,57,58,59,60,61,62,63,64,65,66,67,68,69,70,71,72] are cited in the supplementary materials.

## Abbreviations

The following abbreviations are used in this manuscript:

AMR	Antimicrobial Resistance
HTS	High-Throughput Screening
MRSA	Methicillin-Resistant *S. aureus*
MSSA	Methicillin-Susceptible *S. aureus*
PMM	Photoactive Molecular Materials
MIC	Minimum Inhibitory Concentration
MBC	Minimum Bactericidal Concentration
ATCC	American Type Culture Collection
WHO	World Health Organization
SAR	Structure–Activity Relationship
BHI	Brain Heart Infusion
ADC	Albumin–Dextrose–Catalase supplement
DMSO	Dimethyl Sulfoxide
MTT	[3-(4,5-dimethylthiazol-2-yl)-2,5-diphenyltetrazolium bromide]
LB	Luria–Bertani
EDTA	Ethylenediaminetetraacetic Acid
TKA	Time-Kill Kinetic Assay
SNP	Single Nucleotide Polymorphism
TSA	Tryptic Soy Agar
TSB	Tryptic Soy Broth
DMEM	Dulbecco Modified Eagle Medium
LLOQ	Lower Limit of Quantification
WGS	Whole Genome Sequencing
