# Supplementary material for: New Chemical Scaffold with Antimicrobial Activity Identified in a Screening of Industrial Photoactive Compounds
Source: Antibiotics (Basel). 2026 Mar 20;15(3):321. doi: 10.3390/antibiotics15030321 (PMC13024089; doi:10.3390/antibiotics15030321)
Supplement: Supplementary file 1 [file antibiotics-15-00321-s001.zip › Supp/20260316_Ezquerra-Aznarez et al_supp.pdf]

Supporting information

New chemical scaffold with antimicrobial activity identified in a screening of industrial photoactive compounds

José Manuel Ezquerra-Aznárez[1,*], Raquel Alonso-Román[1,$], Ainhoa Lucía[1,2], Raquel Andreu[3], Santiago Franco[3,*], José A. Aínsa[1,2], Santiago Ramón-García[1,2,4]

1. Department of Microbiology, Pediatrics, Radiology and Public Health, and BIFI, University of Zaragoza, Zaragoza, Spain
2. Spanish Network for Research on Respiratory Diseases (CIBERES), Carlos III Health Institute, Madrid, Spain
3. Department of Organic Chemistry, Faculty of Science, University of Zaragoza & Institute of Nanoscience and Materials of Aragon (INMA), Faculty of Science, CSIC-University of Zaragoza, Zaragoza, Spain
4. Research and Development Agency of Aragon (ARAID) Foundation, Zaragoza, Spain

[$]Present address: Leibniz Institute for Natural Product Research and Infection Biology - Hans-Knöll-Institute, Jena, Germany

*Correspondence: jmezquerraa@unizar.es (J.M.E-A.), sfranco@unizar.es (S.F.)

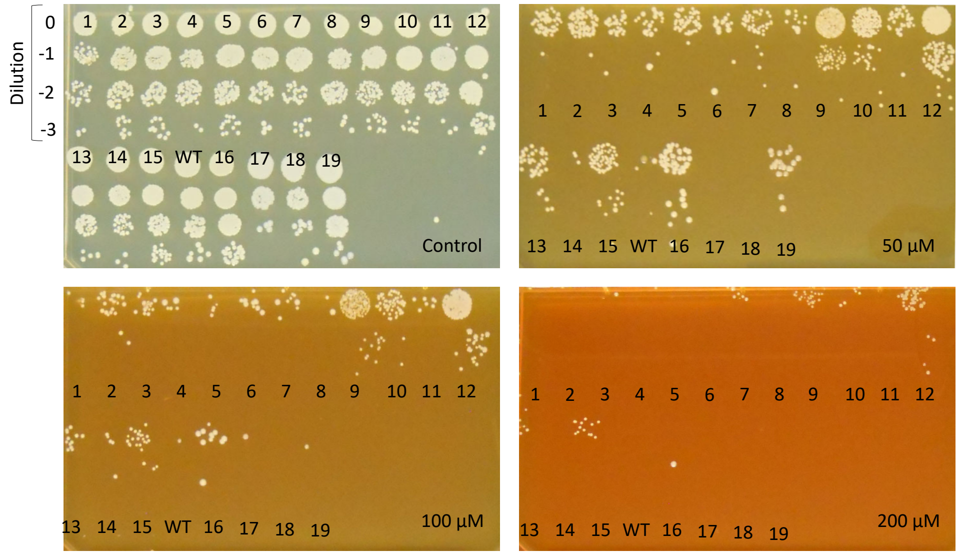

Figure S1. Drop dilution Compound 18 susceptibility testing of nineteen *S. aureus* mutants. Compound18 concentrations are indicated in µM; a Compound 18-free plate was included as a control. Mutants 17 and 18 were discarded for subsequent WGS given that they behaved as the parental wild-type (WT) *S. aureus* ATCC 29213.

Table S2. Bacterial strains used in the screening of the Photoactive Molecular Materials compound collection.

Strain	Type	Incubation time	Culture medium
Corynebacterium diphteriae ATCC 39255	Gram-positive	24 h	BHI
Corynebacterium glutamicum ATCC 13032	Gram-positive	48 h	Müller-Hinton II
Enterococcus faecalis ATCC 19433	Gram-positive	24 h	Müller-Hinton II
Staphylococcus aureus ATCC 29213 (MSSA)	Gram-positive	24 h	Müller-Hinton II
Staphylococcus aureus ATCC 43300 (MRSA)	Gram-positive	24 h	Müller-Hinton II
Staphylococcus epidermidis CECT 231	Gram-positive	24 h	Müller-Hinton II
Klebsiella pneumoniae ATCC 13883	Gram-negative	24 h	Müller-Hinton II
Pseudomonas aeruginosa ATCC 15442	Gram-negative	24 h	Müller-Hinton II
Salmonella typhimurium ATCC 14028	Gram-negative	24 h	Müller-Hinton II
Mycobacteroides abscessus subsp. abscessus ATCC 19977	mycobacteria	72 h	7H9-0.2% glycerol-ADC
M. abscessus subsp. bolletii CCUG 50184	mycobacteria	72 h	7H9-0.2% glycerol-ADC
M. abscessus subsp. massiliense CCUG 48898	mycobacteria	72 h	7H9-0.2% glycerol-ADC
Mycobacterium avium ATCC 25291	mycobacteria	72 h	7H9-0.2% glycerol-ADC
Mycolicibacterium smegmatis mc^{2}155	mycobacteria	72 h	7H9-0.2% glycerol-ADC
Mycobacterium tuberculosis H37Rv	mycobacteria	144 h	7H9-0.2% glycerol-ADC
Escherichia coli efflux pump deficient strains			
E. coli BW25113	Gram-negative	24 h	Müller-Hinton II
E. coli BW25113 Δ*smr*	Gram-negative	24 h	Müller-Hinton II
E. coli BW25113 Δ*emrE*	Gram-negative	24 h	Müller-Hinton II
E. coli BW25113 Δ*mdrA*	Gram-negative	24 h	Müller-Hinton II
E. coli BW25113 Δ*acrB*	Gram-negative	24 h	Müller-Hinton II
E. coli BW25113 Δ*emrE* Δ*mdrA*	Gram-negative	24 h	Müller-Hinton II
E. coli BW25113 Δ*emrE* Δ*acrB*	Gram-negative	24 h	Müller-Hinton II
E. coli BW25113 *emrE* Δ*mdrA* Δ*acrB*	Gram-negative	24 h	Müller-Hinton II

Table S3. Single shot assay of the 4H-pyran-4-ylidene derivatives. Only derivatives active at 50 μM against at least one Gram-positive bacterial species are shown. Compounds active in two assay replicates are shown in black; grey squares indicate compounds were active in only one of the replicates, which were also selected for further analysis. All 4H-pyran-4-ylidene derivatives were inactive against Gram-negative bacteria and mycobacteria (**Table S1**).

	Compound												
	02	04	05	07	11	13	15	18	19	27	28	36	39
C. diphtheriae	■	▒	■		■	■		■	■	■	■	▒	■
C. glutamicum	■				■	▒		■	■	■			
E. faecalis	■		▒		■			■	■	■			
S. aureus (MSSA)	■			■	■			■					
S. epidermidis	■				■			■	■				
S. agalactiae	■		■		■		▒	■	■	■			

Table S4. Mutations identified in *S. aureus* resistant mutants. Mutants 1-15 were isolated from liquid cultures. Mutants 16 and 19 were isolated from agar plates. No SNPs, or short insertions/deletions were identified in mutants 2, 3, 10, 12, 13, and 19.

Mutant	C18 MIC (μM)	Mutation
Wild-type	25	-
1	>200	*rny* P280L
2	200	-
3	>200	-
4	>200	*rny* P280L
5	>200	*rny* P280L
6	>200	*rny* P280L
7	>200	*rny* P280L
8	>200	*rny* P280L
9	>200	*rpsJ* A122V
10	>200	-
11	200	*rny* P280L
12	>200	-
13	200	-
14	>200	*rny* P280L
15	>200	*rny* P280L
16	50	*rny* G240D, LNEJMEBC_01294 I21N
19	100	-

Compound synthesis and characterization

All compounds evaluated in the biological assays were purified by chromatographic methods and characterized by NMR spectroscopy and HRMS. Based on these orthogonal analytical data, the purity of the compounds was estimated to be >95%, which was considered sufficient for biological evaluation.

Scheme synthesis of compound (03): 2-cyano-3-(7-((2,6-diphenyl-4*H*-pyran-4-ylidene)methyl)-2,3-dihydrothieno[3,4-b][1,4]dioxin-5-yl)acrylic acid

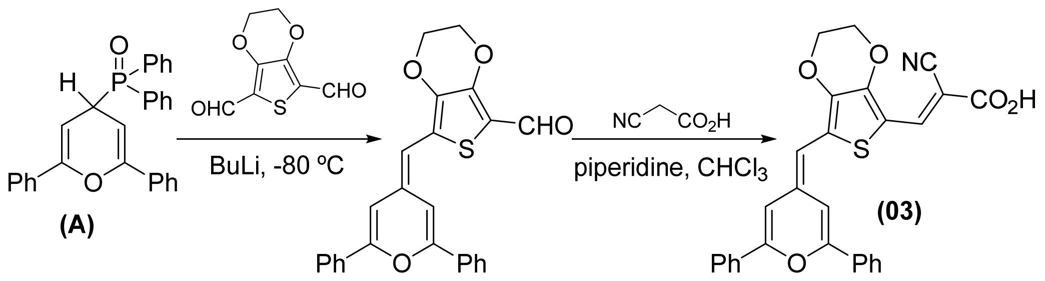

Step 1*:* A solution of (2,6-diphenyl-4*H*-pyran-4-yl)diphenylphosphine oxide (**A**)[16] (1.11 g, 2.55 mmol) in anhydrous THF (30 mL) was cooled to −80 °C under an Ar atmosphere. Subsequently, *n*-BuLi (1.6 M in hexanes, 1.73 mL, 2.77 mmol) was added, and the mixture was stirred for 15 min at −78 °C. After this, a solution of 3,4-ethylenedioxythiophene-2,5-dicarboxaldehyde (commercially available) (502 mg, 2.53 mmol) in anhydrous THF (20 mL) was added, allowing the temperature to gradually return to room temperature over 18 hours. The reaction was then quenched with 5 mL of saturated NH_4Cl and stirred for an additional 30 minutes. The product was extracted with AcOEt (3 × 20 mL), the organic phase was dried over anhydrous $MgSO_4$, and the solvent was removed under reduced pressure. Purification by column chromatography using hexane/CH_2Cl_2 (98:2) as the eluent yielded the desired aldehyde as an orange solid (616 mg, yield: 59%).

Step 2: To a solution of the aldehyde prepared in step 1 (151 mg, 0,33 mmol) and 2-cyanoacetic acid (47 mg, 0.56 mmol) in anhydrous chloroform (7 mL) piperidine (240 μL) was added. The mixture was refluxed for 24 hours under an Ar atmosphere, then cooled to room temperature and acidified to a pH ≈ 2 of with a solution of HCl (1 N). The organic phase was washed with water, and the solvent was evaporated. The residue

was washed with a hexane/CH_2Cl_2 (90:10) mixture and dried to yield a dark violet solid (143 mg, 91%).

M.p.: 255-262 °C. IR (KBr) cm^{-1}: 3675-3135 (COOH), 2204 (C≡N), 1682 (C=C).

^{1}H NMR (300 MHz, dmso-d_6) δ (ppm): 8,11 (s, 1H, **H**C=C(CN)COOH), 8,00−7,80 (m, 4H, H's Ar.), 7,61−7,44 (m, 6H, H's Ar.), 7,23 (br s, 1H, H pyranylidene), 7,15 (brs, 1H, H pyranylidene), 6,26 (s, 1H, H pyranylidene exocyclic), 4,52−4,42 (m, 2H, OCH_2), 4,41−4,32 (m, 2H, CH_2O).

HRMS (ESI$^+$) m/z: Calculated $C_{28}H_{19}NO_5S$: 481.0978. Found: 481.0964

Scheme synthesis of Compound (06): 2-((5-((2,6-diphenyl-4*H*-pyran-4-ylidene)methyl)thiophen-2-yl) methylene)malononitrile

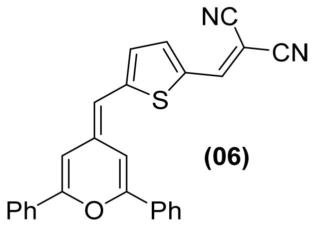

(06)

The starting aldehyde was synthesized in high yield according to a previously described procedure[1]. Piperidine (100 μL) was added to a solution containing the aldehyde precursor (73 mg, 0.20 mmol) and malononitrile (15.9 mg, 0.24 mmol) in 10 mL of ethanol. The reaction mixture was refluxed for 6 hours under an Ar atmosphere and then cooled in an ice bath. The resulting solid was filtered, washed with cold ethanol, and subsequently with a hexane/dichloromethane mixture (95:5). The residue was purified by flash chromatography (using hexane/CH_2Cl_2, 4:6 as the eluent) to afford an intense bluish-green solid (65 mg, 85%).

M.p.: 258-260 °C. IR (Nujol) cm^{-1}: 2210 (C≡N), 1650, 1571 and 1531(C=C, Ar.).

^{1}H NMR (400 MHz, $CDCl_3$) d (ppm): 7.93-7.91 (m, 2H, H's Ar.), 7.82-7.80 (m, 2H, H's Ar.), 7.67 (s, 1H, CH=C(CN)$_2$), 7.58 (d, J = 4.1 Hz, 1H, H thiophene), 7.56-7.46 (m, 6H, H's Ar.), 7.39 (d, J = 1.8 Hz, 1H, H pyranylidene), 6.99 (d, J = 4.1 Hz, 1H, H thiophene), 6.61 (d, J = 1.8 Hz, 1H, H pyranylidene), 6.22 (s, 1H, H pyranylidene exocyclic).

^{13}C NMR (100 MHz, DMSO-d6) δ (ppm): 174.4, 174.1, 159.3, 158.1, 108.4, 102.7, 98.5, 47.1, 38.7, 37.1, 36.7, 27.9, 27.7

HRMS (ESI+) m/z: Calculated $C_{26}H_{17}N_2OS$: 405.1056. Found: 405.1045 $[M+H]^+$.

Scheme synthesis of Compound (07): ethyl hydrogen-(1-cyano-2-(5-((2,6-diphenyl-4*H*-pyran-4-ylidene)methyl)thiophen-2-yl)vinyl)phosphonate

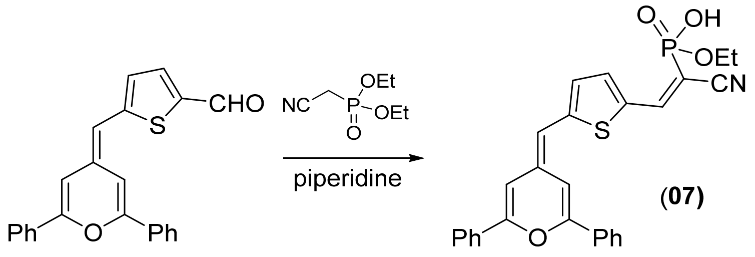

The starting aldehyde was synthesized in high yield according to a previously described procedure[1]. Piperidine (400 µL) was added to a solution of aldehyde precursor (206 mg, 0.578 mmol) and diethyl cyanomethylphosphonate (114 µL, 0.69 mmol) in 10 mL of anhydrous acetonitrile. The reaction mixture was heated to reflux under an Ar atmosphere for 24 hours. After solvent removal, the residue was purified by reversed-phase C18 chromatography ($CH_3CN/AcONH_4$ 20 mM 7:3 as eluent) to give a maroon solid (153 mg, 54%) as a Z/E mixture.

M.p.: 200-203 °C.

^{1}H NMR (400 MHz, DMSO-d_6) δ (ppm): 8.11-7.84 (m, 5H, H's Ar. + **H**C=C(CN)P), 7.81 (d, *J* = 4.1 Hz, 1H, H thiophene), 7.63-7.44 (m, 6H, H's Ar.), 7.28 (br s, 1H, H pyranylidene), 7.22 (d, *J* = 4.1 Hz, 1H, H thiophene), 7.02 (br s, 1H, H pyranylidene), 6.36 (s, 1H, H pyranylidene exocyclic), 4.04-3.89 (m, 2H, OCH_2), 1.25 (t, *J* = 7.0 Hz, 3H, CH_3).

^{13}C NMR (100 MHz, DMSO-d_6) δ (ppm): 154.2, 151.6, 149.7, 147.5, 147.4, 138.5 (x2), 133.4, 133.2, 131.8, 131.5, 130.5, 129.8, 129.1, 128.9, 126.3, 124.9, 124.5, 117.9, 117.8, 108.6, 107.3, 102.3, 95.3, 93.4, 61.5, 61.4, 16.3, 16.2.

HRMS (ESI$^+$) m/z: Calculated $C_{27}H_{23}NO_4PS$: 488.1079. Found: 488.1080

Scheme synthesis of Compound (10): (1-cyano-2-(2-((2,6-di-tert-butyl-4*H*-pyran-4-ylidene)methyl) thiazol-5-yl)vinyl)phosphonic acid

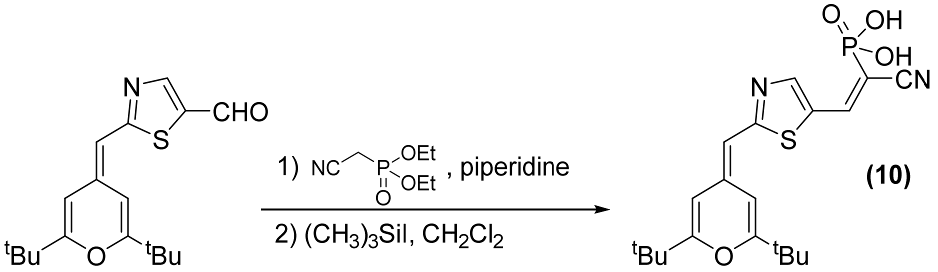

The starting aldehyde was synthesized in high yield according to a procedure previously described[15]. Piperidine (250 μL) was added to a solution of aldehyde precursor (131 mg, 0.356 mmol) and diethyl cyanomethylphosphonate (71 mL, 0.428 mmol) in 8 mL of anhydrous acetonitrile. The reaction mixture was heated to reflux for 24 hours under an Ar atmosphere. After removing the solvent, the residue was purified by flash chromatography (CH_2Cl_2/AcOEt, 10:1 as eluent) to give a red oil (142 mg, 85%).

Subsequently, trimethylsilyl iodide (206 μL, 1.397 mmol) was added to a solution of the intermediate obtained in the previous step (108 mg, 0.226 mmol) in anhydrous dichloromethane (15 mL). The resulting mixture was refluxed for 2 hours. Subsequently, 10 mL of methanol was added, and the mixture was stirred at room temperature for 1 hour. After solvent evaporation, the residue was washed with a hexane/CH_2Cl_2 mixture (9:1) until the washing solution became colorless. The resulting residue was dried under vacuum to yield a maroon solid (85 mg, 89%).

M.p.: 193-196 °C.

^{1}H NMR (400 MHz, DMSO-d_6) δ (ppm): 8.28 (s, 1H, **H**C=C(CN)P), 7.98 (s, 1H, OH), 7.92 (s, 1H, H thiazole), 7.52 (br s, 1H, H pyranylidene), 6.14 (br s, 1H, H pyranylidene), 6.07 (s, 1H, H pyranylidene exocyclic), 1.22 (s, 9H, $(CH_3)_3$), 1.19 (s, 9H, $(CH_3)_3$).

^{13}C NMR: The product is poorly soluble. Its ^{13}C-NMR spectrum could not be determined

HRMS (ESI$^+$) m/z: Calculated $C_{20}H_{25}N_2NaO_4PS$: 443.1170. Found: 443.1159

Scheme synthesis of Compound (13): 5-(2-(2,6-di-*tert*-butyl-4*H*-pyran-4-ylidene)ethylidene)-4-(4-methoxyphenyl)-2-oxo-2,5-dihydrofuran-3-carbonitrile

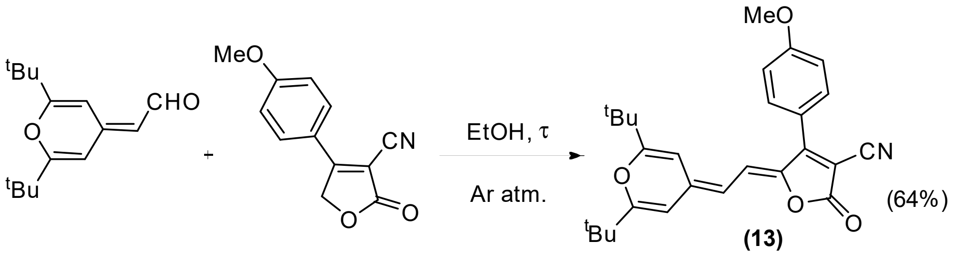

To a solution of 2,6-di-*tert*-butyl-4-formylmethylene-4*H*-pyran[17] (141 mg, 0.60 mmol) in absolute ethanol (5 mL), 4-(4-methoxyphenyl)-2-oxo-2,5-dihydrofuran-3-carbonitrile[18] (129 mg, 0.60 mmol) was added. The mixture was refluxed under Ar with exclusion of light for 16.5 hours. After cooling, the resulting solid was isolated by filtration, washed with cold ethanol, and then with mixture of cold pentane/CH_2Cl_2 9.5:0.5, affording a dark blue solid (166,3 mg; yield: 64%).

M.p.: 197−200 °C. **IR** (nujol): $\bar{\nu}$ (cm^{-1}) 2212 (C≡N), 1739 (C=O), 1654 (C=C), 1609 and 1583 (C=C, Ar).

^{1}H NMR (400 MHz, $CDCl_3$) δ (ppm): 7.60−7.55 (m, 2H, H's Ar.), 7.09−7.04 (m, 2H, H's Ar.), 6.67 (d, *J* = 13.0 Hz, 1H, CH=acceptor), 6.18 (d, *J* = 1.8 Hz, 1H, H pyranylidene), 6.11 (d, *J* = 13.0 Hz, 1H, CH=pyranylidene), 6.04 (d, *J* = 1.8 Hz, 1H, H pyranylidene), 3.90 (s, 3H, OCH_3), 1.25 (s, 9H, $C(CH_3)_3$), 1.24 (s, 9H, $C(CH_3)_3$).

^{13}C NMR (100 MHz, $CDCl_3$): δ (ppm) 169.3, 168.9, 165.8, 162.1, 157.5, 144.6, 141.5, 130.6, 121.4, 120.4, 114.7, 114.0, 108.3, 107.3, 100.1, 90.3, 55.6, 36.3, 36.0, 27.8, 27.5.

Scheme synthesis of compound (14): 2-cyano-3-(7-(4-((2,6-diphenyl-4*H*-pyran-4-ylidene)methyl)phenyl) benzo[c][1,2,5]thiadiazol-4-yl)acrylic acid

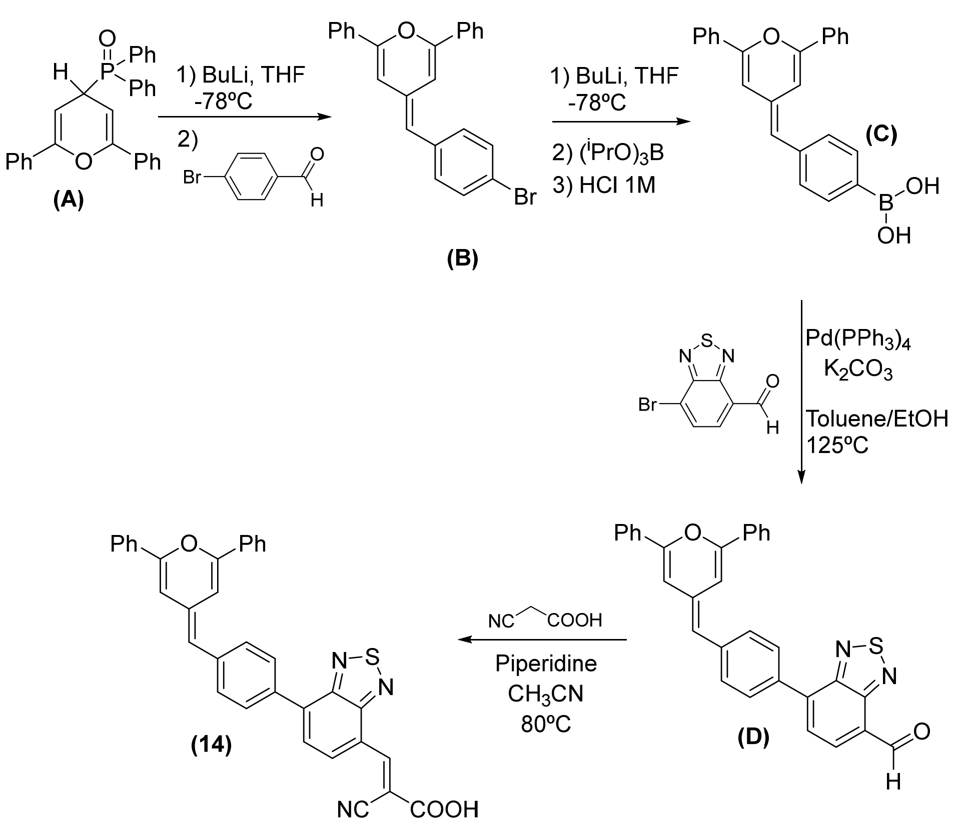

Compound (B): 4-(4-bromobenzylidene)-2,6-diphenyl-4*H*-pyran

A solution of (2,6-diphenyl-4*H*-pyran-4-yl)diphenylphosphine oxide **A**[16] (547 mg, 1.27 mmol) in anhydrous THF (10 mL) was cooled to −78°C under an Ar atmosphere. Subsequently, n-BuLi (1.6 M in hexanes, 0.86 mL, 1.4 mmol) was added, and the solution was stirred for 20 min at −78 °C. Afterward, a solution of *p*-bromobenzaldehyde (commercially available) (234.8 mg, 1.27 mmol) in anhydrous THF (10 mL) was added, allowing the temperature to gradually return to room temperature over 18 hours. The reaction was quenched with 5 mL of a saturated NH_4Cl solution and stirred for an additional 30 minutes. The product was extracted with CH_2Cl_2 (2 × 15 mL), the organic phase was dried over anhydrous $MgSO_4$, and the solvent was removed under reduced pressure. The product was purified by column chromatography with hexane/CH_2Cl_2 (9:1) as the eluent, yielding the desired product as an orange solid (367 mg, yield: 74%).

M.p.: (°C): 194−196. IR (KBr): $\bar{\nu}$ (cm^{-1}) 1657, 1600, 1579 and 1545 (C=C, Ar), 1075 (Csp^2-Br).

^{1}H NMR (300 MHz, CD_2Cl_2) δ (ppm): 7.82−7.76 (m, 4H, H's Ar.), 7.50−7.41 (m, 8H, H's Ar.), 7.32−7.26 (m, 2H, H's Ar.), 6.95 (dd, J_1 = 1.8 Hz, J_2 = 0.6 Hz, 1H, H pyranylidene), 6.45 (d, J = 1.8 Hz, 1H, H pyranylidene), 5.88 (s, 1H, H pyranylidene exocyclic).

^{13}C NMR (100 MHz, CD_2Cl_2): δ (ppm): 153.6, 151.5, 138.1, 133.8, 133.6, 132.0, 130.5, 130.0, 129.9, 129.7, 129.2, 125.4, 125.0, 119.2, 113.3, 108.8, 102.1.

HRMS (ESI$^+$): m/z calculated for $C_{24}H_{18}BrO$ ([M+H]$^+$) 401.0536, found 401.0546.

Compound (C): 4-((2,6-diphenyl-4*H*-pyran-4-ylidene)methyl)phenylboronic acid

A solution of compound **B** (243 mg, 0.67 mmol) in anhydrous THF (8 mL) was cooled at −78°C under an Ar atmosphere. Subsequently, *n*-BuLi (1.6 M in hexanes, 0.54 mL, 0.73 mmol) was added, and the solution was stirred for 1h to −78 °C. Afterwards, triisopropyl borate (0.21 mL, 0.92 mmol) was added, and the temperature was allowed to gradually return to room temperature over 22 hours. Next, 6 mL of HCl (1 N) were added and the mixture was stirred for 30 min. The product was extracted with CH_2Cl_2 (2 × 20 mL), the organic phase was dried over anhydrous $MgSO_4$, and the solvent was removed under reduced pressure. To the crude oil, 10 mL of ethyl acetate were added, resulting in a yellow solid, which was then filtered and washed with cold ethyl acetate, yielding a yellow solid (68.4 mg, yield: 28%).

M.p.: (°C): 217−220. IR (KBr): $\bar{\nu}$ (cm^{-1}) 3243 (BO-H), 1658 (C=C, Ar), 1591 and 1572 (C=C, Ar), 1376 (B-O).

^{1}H NMR (400 MHz, THF-d_8) δ (ppm): 7.84−7.77 (m, 4H, H's Ar.), 7.75 (d, J = 8.1 Hz, 2H, H's Ar.), 7.45−7.33 (m, 8H, H's Ar.), 7.08 (d, J = 1.8 Hz, 1H, H pyranylidene), 7.04 (s, 2H, $(OH)_2$), 6.59 (d, J = 1.8 Hz, 1H, H pyranylidene), 5.97 (s, 1H, H pyranylidene exocyclic).

^{13}C NMR (100 MHz, THF-d_8) δ (ppm): 154.0, 152.0, 141.6, 135.6, 135.0, 134.8, 130.8, 130.7, 130.6, 130.3, 130.0, 129.9, 128.1, 126.2, 125.8, 116.4, 110.0, 103.5.

HRMS (ESI$^+$): m/z calculated for $C_{24}H_{19}BO_3$ ($[M]^{+\cdot}$) 366.1426, found 366.1449.

Compound (D): 7-(4-((2,6-diphenyl-4*H*-pyran-4-ylidene)methyl)phenyl)benzo[*c*] [1,2,5]thiadiazole-4-carbaldehyde

A solution of boronic acid **C** (69 mg, 0.189 mmol), 7-bromobenzo[c][1,2,5]thiadiazole-4-carbaldehyde[19]. (33.5 mg, 0.164 mmol), K_2CO_3 (239 mg, 1.64 mmol) and tetrakis (triphenylphosphine)palladium(0) (19.1 mg, 0.016 mmol) in 25 mL of toluene and 5 mL of ethanol, previously deoxygenated, was heated at 125 °C for 45 min. After cooling, the solvent was evaporated and the product was purified by column chromatography using hexane/CH_2Cl_2 (1:9) as eluent, yielding a dark red-purple solid (55 mg, yield: 83%).

M.p.: (°C): 188−190. IR (KBr): $\bar{\nu}$ (cm-1) 2921 and 2848 (C−H), 1680 (C=O), 1656 (C=N), 1583 and 1535 (C=C, Ar).

^{1}H NMR (400 MHz, CD_2Cl_2) δ (ppm): 10.78 (s, 1H, CHO), 8.30 (d, J = 7.4 Hz, 1H, H benzothiadiazole), 8.09−8.06 (m, 2H, H's Ar.), 7.94 (d, J = 7.4 Hz, 1H, H benzothiadiazole), 7.85−7.80 (m, 4H, H's Ar.), 7.62−7.60 (m, 2H, H's Ar.), 7.51−7.40 (m, 6H, H's Ar.), 7.15 (d, J = 1.9 Hz, 1H, H pyranylidene), 6.53 (d, J = 1.9 Hz, 1H, H pyranylidene), 6.04 (s, 1H, H pyranylidene exocyclic).

HRMS (ESI$^+$): m/z calculated for $C_{31}H_{20}N_2NaO_2S$ ($[M+Na]^+$) 507.1138, found 507.1149.

Compound (14) from Compound (D): 2-cyano-3-(7-(4-((2,6-diphenyl-4*H*-pyran-4-ylidene)methyl)phenyl)benzo[c][1,2,5]thiadiazole-4-yl)acrylic acid

A solution of aldehyde **D** (46 mg, 0.095 mmol), cyanoacetic acid (12 mg, 0.142 mmol), and piperidine (62 μL; 0.627 mmol) in dried acetonitrile (6 mL) was heated at 85 °C (TLC monitoring; eluent: hexane/CH_2Cl_2 (1:9)) for 24 hours. After cooling, the resulting solid was isolated by centrifugation at 40,000 rpm. The product was then purified by washing with a mixture of hexane/CH_2Cl_2 (9:1). The solid was recovered by centrifugation (40,000 rpm), yielding a dark red solid. (46 mg, yield: 89%).

M.p.: (°C): 219−221. IR (KBr): $\bar{\nu}$ (cm^{-1}) 3433 (O-H, broad), 2213 (C≡N), 1655 (C=O), 1638 (C=N), 1577 (C=C, Ar), 1529 (C=C, Ar), 1337 (C-O).

^{1}H NMR (400 MHz, DMSO-d_6) δ (ppm): 8.74 (s, 1H, CH(CN)COOH), 8.59 (d, *J* = 7.5 Hz, 1H, H benzothiadiazole), 8.13 (d, *J* = 8.3 Hz, 2H, H's *para*-system), 8.10 (d, *J* = 7.5 Hz, 1H, H benzothiadiazole), 7.92–7.88 (m, 2H, H's Ar.), 7.64 (d, *J* = 8.3 Hz, 2H, H's *para*-system), 7.56–7.44 (m, 6H, H's Ar), 7,16 (br s, 1H, H pyranylidene), 6.88 (d, *J* = 1.2 Hz, 1H, H pyranylidene), 6.12 (s, 1H, H pyranylidene exocyclic).

^{13}C NMR (100 MHz, DMSO-d_6) δ (ppm): 162.4, 154.7, 152.9, 152.7, 150.3, 140.6, 138.9, 134.8, 133.5, 132.8, 132.6, 130.1, 129.9, 129.8, 129.7, 129.3, 129.2, 128.3, 128.1, 127.6, 125.2, 125.1, 124.6, 119.6, 114.5, 109.2, 102.0. HRMS (ESI$^-$): m/z calculated for $C_{33}H_{20}N_3OS$ ($[M\text{-}COOH]^-$) 506.1333, found 506.1328.

Scheme synthesis of Compound (20) and Compound (21)

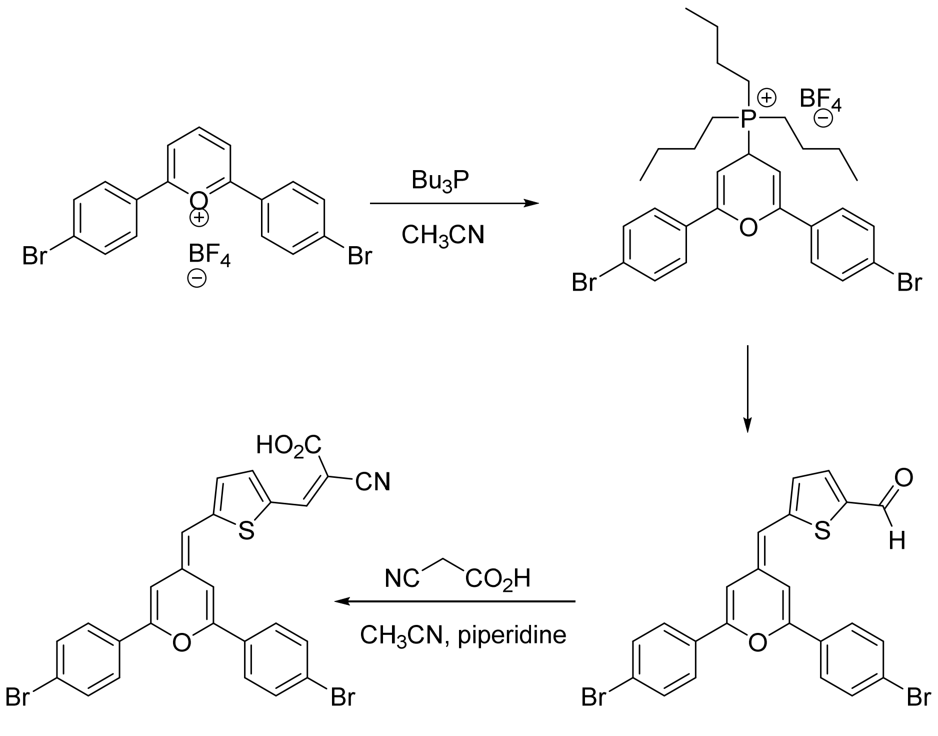

(21) (20)

4'-Bromo-2,6-diphenylpyrylium tetrafluoroborate

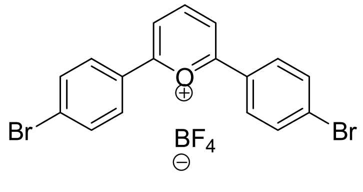

To a solution of ethyl orthoformate (5 mL, 30 mmol) and 2-bromo-1-phenylethan-1-one (1.998 g, 10 mmol), HBF_4 (0.5 mL) and acetic anhydride (5 mL) were added at 0 °C. Then, the mixture was stirred for 90 minutes at room temperature and then to 50 °C for 30 minutes. The reaction was cooled to room temperature and the yellow solid filtered and washed with cold ethyl ether. Yield: 1.077 g, 45%

M.p.: 225 – 230 °C. **IR** (KBr) cm^{-1}: 3098,51 (Csp^2-H), 1504,33 (C=C).

^{1}H NMR (300 MHz, acetone-d6) δ (ppm): 9.34 (t, J_1 = 8.3 Hz, 1H), 8.99 (d, J = 8.3 Hz, 2H), 8.50 (dt, J_1 = 8.8 Hz, J_2 = 2.3 Hz, 4H, H's *para*-system), 8.01 (dt, J_1 = 8.8 Hz, J_2 = 2.3 Hz, 4H, H's *para*-system)

^{13}C NMR: The product is unstable in deuterated solvents and poorly soluble. Its ^{13}C-NMR spectrum could not be determined.

Tetrafluoroborate of tributyl(2,6-bis(4-bromophenyl)-4*H*-pyran-4-yl) phosphonium

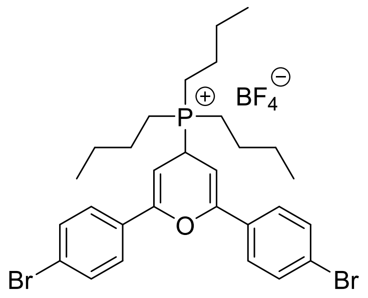

To a solution of pyrilium salt obtained in step 1 (0.573 g, 1.20 mmol) in anhydrous acetonitrile (3.5 mL) *n*-tributylphosphine (0.31 mL, 1.24 mmol) was added and the mixture was stirred for 2 h at room temperature. Then, 150 mL of cold ethyl ether were added and the solid was filtered and washed with cold ethyl ether. Yield: 749 mg, 92%, as an ochre-colored solid.

M.p.: 192 – 200 °C. **IR** (KBr) cm^{-1}: 2958,54 (Csp^3-H), 1488,65 (C=C).

^{1}H NMR (400 MHz, Acetone-d_6) δ (ppm): 7.81 (dt, J_1 = 8.4 Hz, J_2 = 1.8 Hz, 4H, H's Ar.), 7.67 (dt, J_1 = 8.8 Hz, J_2 = 2.2 Hz, 4H, H's Ar.), 5,97 (dd, J_1 = 2.90 Hz, J_2 = 4.9 Hz, 2H, H pyranylidene), 4.7 (dt, J_1 = 14.4 Hz, J_2 = 5.2 Hz, 1H, H-P), 2.61 (m, 6H, 3×CH_2-P), 1.80 (m, 6H, 3×CH_2), 1.51 (m, 6H, 3×CH_2), 0.92 (t, J = 7.3 Hz, 9H, 3×CH_3).

^{13}**C NMR** (75 MHz, Acetone-d6) δ (ppm): 154.4, 154.3, 133.6, 128.8, 125.3, 92.6, 92.5, 32.0, 31.4, 25.7, 25.5, 25.2, 25.1, 18.4, 17.8, 14.6.

HRMS (ESI$^+$) m/z: Calculated for $C_{29}H_{38}Br_2OP$ [M]: 591.1022. Found: 591.1052.

Scheme synthesis of Compound (20): 5-((2,6-Bis(4-bromophenyl)-4*H*-pyran-4-ylidene)methyl)thiophene-2-carbaldehyde

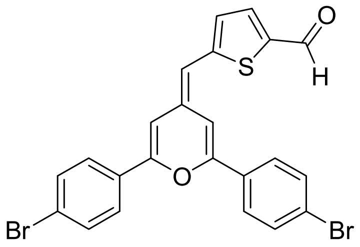

To a solution of the phosphonium salt (250 mg, 0.368 mmol) and potassium tert-butoxide (49.1 mg, 0.438 mmol) in anhydrous THF (15 mL) and the mixture was stirred for 15 minutes. Then thiophene-2,5-dicarbaldehyde (61 mg, 0.436 mmol) in THF (5 mL) was added and the mixture of reaction was stirred for 24 h. The reaction was quenched with a saturated solution of NH_4Cl and extracted with ethyl acetate. The organic layer was dried over $MgSO_4$ and purified by silica gel column chromatography (20% ethyl acetate in hexanes). Yield: 144 mg, 76% as a reddish solid.

M.p.: 209 – 214 ^{0}C. **IR** (KBr) cm^{-1}: 1652,42 (C=O), 1558,45 (C=C).

^{1}**H NMR** (400 MHz, CH_2Cl_2) δ (ppm): 9.85 (s, 1H, CHO), 7.78 (dt, J_1 = 8.8 Hz, J_2 = 2.2 Hz, 2H, H's Ar.), 7.67 (m, 7H, H's Ar. + H thiophene), 7.25 (dd, J_1 = 0.65 Hz, J_2 = 1.97 Hz, 1H, H pyranylidene), 7.07 (dd, J_1 = 0.58 Hz, J_2 = 4.05 Hz, 1H, H thiophene), 6.58 (d, J = 1.62, 1H, H pyranylidene), 6.25 (s, 1H, H pyranylidene exocyclic).

^{13}**C NMR** (100 MHz, CH_2Cl_2): 182.5, 154.4, 152.0, 151.9, 140.4, 137.8, 132.7, 132.5, 132.3, 132.0, 131.9, 127.4, 127.3, 126.7, 125.0, 124.4, 109.5, 108.5, 103.5.

HRMS (ESI$^+$) m/z: Calculated for $C_{23}H_{15}Br_2O_2S$ $[M+H]^+$: 512.9154. Found: 512.9165.

Compound (21): 3-(5-((2,6-Bis(4-bromophenyl)-4*H*-pyran-4-ylidene)methyl)thiophen-2-yl)-2-cyanoacrylic acid

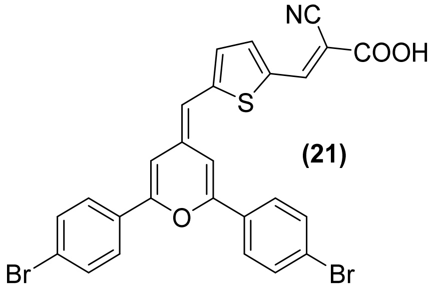

(21)

To a solution of the aldehyde prepared in the previous step (106.2 mg, 0.207 mmol) and cyanoacetic acid (35 mg, 0.411 mmol) in anhydrous acetonitrile (15 mL) under an argon atmosphere, piperidine (150 μL, 1.4 mmol) was added. The mixture was refluxed for 72 h and then cooled in an ice bath. Acetic acid (5 mL) was then added, and the resulting solid was filtered and washed with cold acetonitrile and hexane. Yield: 109.3 mg, 91%, as a dark purple solid.

M.p.: 220 – 222 ^{0}C. **IR** (KBr) cm^{-1}: 2209 C≡N), 1650 (C=O), 1540 (C=C).

^{1}**H-NMR** (400 MHz, THF-d$_8$) δ (ppm): 8.30 (s, 1H, **H**C=C(CN)COOH), 7.87 (d, J = 8.7 Hz, 2H, H's Ar.), 7.80 (d, J = 8.7 Hz, 2H, H's Ar.), 7.74 (d, J = 4.2 Hz, 1H, H thiophene), 7.66 (m, 4H), 7.48 (d, J = 1.6 Hz, 1H, H pyranylidene), 7.11 (d, J = 4.2 Hz, 1H, H thiophene), 6,85 (s, 1H, H pyranylidene), 6.33 (s, 1H, H pyranylidene exocyclic).

^{13}**C-NMR** (100 MHz, THF) δ (ppm): 164.7, 155.1, 152.8, 152.7, 146.1, 139.6, 134.2, 133.2, 133.0, 132.7, 132.6, 127.9, 127.5, 125.4, 124.7, 117.8, 110.3, 109.3, 104.3, 97.4.

HRMS (ESI$^-$) m/z: Calculated for $C_{25}H_{14}Br_2NOS$ [M-CO$_2$-H]$^-$: 533.9168. Found: 533.9172. Calculated for $C_{52}H_{29}Br_4N_2O_6S_2$ [2M-H]$^-$: 1156.8206. Found: 1156.8179.

Scheme synthesis of Compound (26)

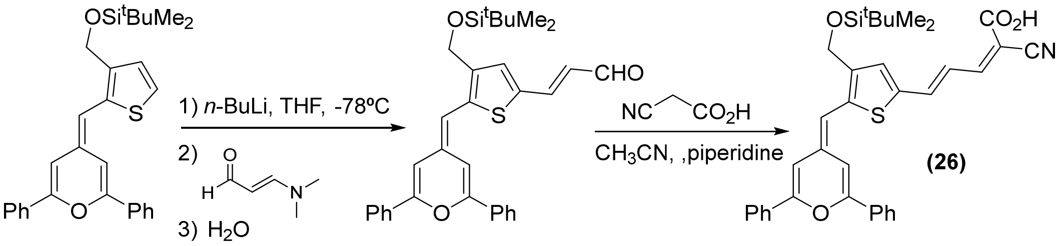

***tert*-butyl((2-((2,6-diphenyl-4*H*-pyran-4-ylidene)methyl)thiophen-3-yl)methoxy) dimethylsilane**

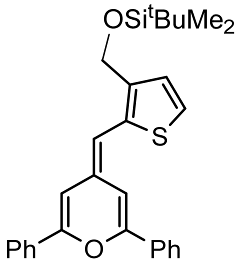

A solution of (2,6-diphenyl-4*H*-pyran-4-yl)diphenylphosphine oxide **A**[16] (680 mg, 1.56 mmol) in anhydrous THF (12 mL) was cooled to −78°C under an Ar atmosphere. Subsequently, *n*-BuLi (1.6 M in hexanes, 1.2 mL, 1.92 mmol) was added, and the solution was stirred for 20 min at −78 °C. Then, a solution of 3-*tert*-butyldimethylsilyloxymethyl-2-thiophenecarboxaldehyde[20] (472 mg, 1.84 mmol) in anhydrous THF (5 mL) was added, allowing the temperature to gradually return to 0 °C in 3 h. Then, a saturated solution of NH_4Cl (15 mL) was added and the organic phase was extracted with ethyl acetate, dried over $MgSO_4$ and evaporated under reduced pressure. The product was purified by silica gel column chromatography (3% ethyl acetate in hexane). Yield: 676 mg, 91%, as a brown solid.

M.p.: 110−114 °C. **IR** (KBr): *cm*$^{-1}$ 1652 (C=C).

^{1}H NMR (400 MHz, $CDCl_3$): δ (ppm) 7.74−7.86 (m, 4H, H’s Ar.), 7.36−7.51 (m, 6H, H’s Ar.), 7.15 (dd, *J*=2.0 Hz, 1H, H pyranylidene), 7.13 (d, *J*=5.2 Hz, 1H, H thiophene), 7.08 (d, *J*=5.2 Hz, 1H, H thiophene), 6.44 (d, *J*=2.0 Hz, 1H, H pyranylidene), 6.07 (s, 1H, H pyranylidene exocyclic), 4.76 (s, 2H, OCH_2), 0.94 (s, 9H, $(CH_3)_3$), 0.10 (s, 6H, 2 × CH_3).

^{13}C NMR (100 MHz, $CDCl_3$): δ (ppm) 152.9, 150.6, 138.0, 135.7, 133.4, 133.2, 129.3, 129.0, 128.6, 128.2, 124.9, 124.4, 121.2, 108.5, 105.5, 102.6, 59.9, 26.0, –5.2.

HRMS (ESI$^+$): *m/z* Calculated for $[C_{29}H_{33}O_2SSi]^+$: 473.1965, found: 473.1947. Calculated for $[C_{29}H_{32}NaO_2SSi]^+$: 495.1784, found: 495.1774. Calculated for $[C_{29}H_{32}KO_2SSi]^+$: 511.1524, found: 511.1516.

3-(4-(((tert-butyldimethylsilyl)oxy)methyl)-5-((2,6-diphenyl-4*H*-pyran-4-ylidene)-methyl)thiophen-2-yl)acrylaldehyde

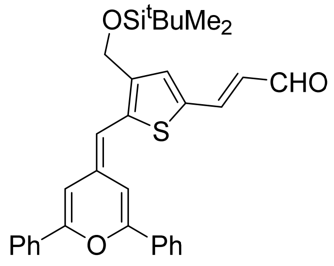

To a solution of the compound prepared in the previous step (1.04 g; 2,21 mmol) in anhydrous THF (5 mL) cooled to –78 °C under an argon atmosphere, *n*-BuLi (1.65 mL, 2.64 mmol) was added. The resulting mixture was stirred for 1 h to –78 °C and then 1 h at 0 °C. The reaction was cooled again to –78 °C and a solution of *N*,*N*-dimethylacrolein (0.66 mL, 5.96 mmol) was added. The mixture was stirred, and the temperature was allowed to slowly rise to room temperature over 3h. Then, H_2O (0.5 mL) was added, and the reaction was stirred overnight. The THF was evaporated, and the organic phase was extracted with ethyl acetate (3 × 50 mL), washed with saturated NaCl, and dried over $MgSO_4$. The solvent was evaporated under reduced pressure and the crude product was purified by silica gel column chromatography (5% ethyl acetate in hexanes). Yield: 590 mg, 51% of a red solid.

IR (KBr): *cm^{-1}* 1656 (C=O), 1114 (C−O).

^{1}H NMR (400 MHz, $CDCl_3$): δ (ppm) 9.60 (d, *J*=7.8 Hz, 1H, CHO), 7.88−7.73 (m, 8H, H's Ar.), 7.58−7.40 (m, 4H, H's Ar. + H thiophene + HC=), 7.21 (d, *J*=1.9 Hz, 1H, H pyranylidene), 6.50 (d, *J*=1.9 Hz, 1H, H pyranylidene), 6.46 (dd, J_1=15.3 Hz, J_2=7.8 Hz, 1H, =C**H**-CHO), 6.04 (s, 1H, H pyranylidene exocyclic), 4.72 (s, 2H, OCH_2), 0.96 (s, 9H, $(CH_3)_3$), 0.13 (s, 6H, 2 × CH_3).

^{13}C NMR (100 MHz, $CDCl_3$): δ (ppm) 192.7, 154.7, 152.1, 144.5, 142.0, 139.5, 134.2, 133.8, 132.8, 132.7, 131.1, 130.0, 129.5, 128.9, 128.7, 125.1, 125.0, 124.6, 109.0, 105.0, 102.9, 59.7, 25.9, 18.4, –5.2

HRMS (ESI$^+$): *m*/*z* Calculated for $C_{32}H_{35}O_3SSi$ $[M+H]^+$ 527.2071; found: 527.2054.

Compound (26): 5-{4-[(tert-Butyldimethylsilyloxy)methyl]-5-[(2,6-diphenyl-4*H*-pyran-4-ylidene)methyl]thiophen-2-yl}-2-cyanopenta-2,4-dienoic acid

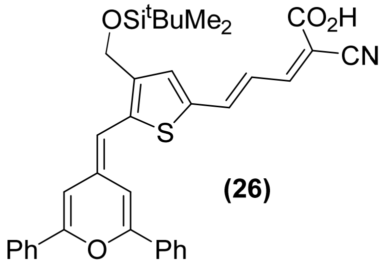

(26)

To a solution of the aldehyde prepared in the previous step (130 mg, 0.25 mmol) and cyanoacetic acid (33 mg, 0.39 mmol) in chloroform (5 mL) under an argon atmosphere, piperidine (162 μL, 1.62 mmol) was added. The mixture was refluxed for 2 h and then cooled in an ice bath. Then, acetic acid (5 mL) and H_2O (20 mL) were added, and the organic phase was separated, dried over $MgSO_4$, and the solvent evaporated under reduced pressure. The resulting solid was washed five times with a mixture of CH_2Cl_2/Hexane (6:96). Yield: 142 mg, 96%, as a brown solid.

IR (KBr): *cm*$^{-1}$ 3700–3000 (COO–H), 2216 (C≡N), 1675 (C=O), 1648 (C=C), 1234 (C-O).

^{1}H NMR (400 MHz, DMSO-d$_6$): δ (ppm) 7.94–7.80 (m, 5H, H's Ar. + **H**C=C(CN)COOH), 7.6 (d, *J*=14.7 Hz, 1H, thiophene-CH), 7.57–7.45 (m, 6H, H's Ar.), 7.37 (s, 1H, H thiophene), 7.16 (d, J = 1.0 Hz, 1H, H pyranylidene), 6.96 (d, J=1.0 Hz, 1H, H pyranylidene), 6.77 (dd, J_1=14.7 Hz, J_2=11.9 Hz, 1H, thiophene-CH=**CH**), 6.28 (s, 1H, H pyranylidene exocyclic), 4.70 (s, 2H, OCH_2), 0.89 (s, 9H, $(CH_3)_3$), 0.09 (s, 6H, 2 × CH_3).

^{13}C NMR (100 MHz, $CDCl_3$): δ (ppm) 154.1, 151.1, 140.1, 135.6, 133.2, 133.1, 132.3, 132.1, 130.6, 130.0, 129.9, 129.3, 129.2, 125.2, 124.6, 120.9, 117.4, 109.2, 106.1, 102.6, 59.1, 26.0, 18.2, -5.0.

EM (MALDI$^+$): *m/z* 593 $C_{35}H_{35}NO_4SSi$ $[M]^{+\cdot}$. HRMS (ESI$^+$): *m/z* Calculated for $C_{35}H_{36}NO_4SSi$ $[M+H]^+$ 594.061, found 594.2061.

Scheme synthesis of Compound (27): 3-(4-(((tert-butyldimethylsilyl)oxy)methyl)-5-((2,6-di-*tert*-butyl-4*H*-pyran-4-ylidene)methyl)thiophen-2-yl)-2-cyanoacrylic acid

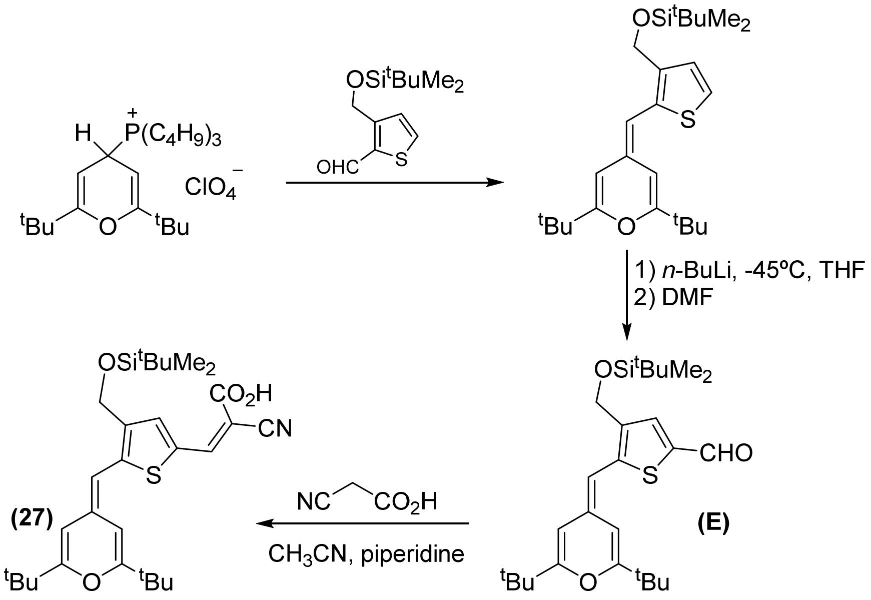

Step 1:

A solution of (2,6-di-*tert*-butyl-4*H*-pyran-4-yl)tributylphosphonium perchlorate[21] (400 mg; 0.808 mmol) in anhydrous THF (10 mL) was prepared, purged with argon, and cooled to –78 °C. To the solution, *n*-BuLi (1.6 M in hexanes) (0.55 mL; 0.888 mmol) was added dropwise and the resulting mixture was stirred for 15 minutes. Then 3-(*tert*-butyldimethylsilyloxymethyl)thiophene-2-carbaldehyde[20] (159 mg, 0.621 mmol) in anhydrous THF (5 mL) was added dropwise and the mixture was progressively heated to reach room temperature after 15 hours (TLC monitoring with 30% ethyl acetate in hexanes). A saturated solution of NH_4Cl (20 mL) was then added to quench the reaction and the mixture was stirred for 5 minutes at room temperature. The organic layer was dried over $MgSO_4$, and the solvent was evaporated under reduced pressure. The crude product was filtered through a short silica gel column chromatography (30% ethyl acetate in hexanes) and used in the next step without additional purification. Yield: yellow oil (240 mg, 89%).

Step 2: Synthesis of compound E

A solution of the compound prepared in step 1 (240 mg, 0.55 mmol) in anhydrous THF (10 mL), was prepared, purged with argon and cooled to –45 °C. To the solution, n-

BuLi (1.6 M in hexanes) (0.63 mL, 1.1 mmol) was added dropwise, and the resulting mixture was stirred for 1h. Then DMF was added (128 μL, 1.65 mmol) and the mixture was progressively heated to reach room temperature in 12 h. A saturated solution of NH_4Cl (20 mL) was added and the mixture was stirred for 5 minutes. The solution was extracted with CH_2Cl_2 (2 × 30 mL), dried over $MgSO_4$ and the solvent was evaporated under reduced pressure. Pure compound was obtained by silica gel column chromatography (10% ethyl acetate in hexanes). Yield: 174 mg, 68%, as orange oil.

^{1}H NMR (400 MHz, $CDCl_3$) δ 9.75 (s, 1H, CHO), 7.65 (s, 1H, H thiophene), 6.55 (d, J = 1.6 Hz, 1H, H pyranylidene), 5.81-5.78 (m, 2H, H pyranylidene + H pyranylidene exocyclic), 4.69 (s, 2H, OCH_2), 1.27 (s, 9H, $(CH_3)_3$), 1.22 (s, 9H, $(CH_3)_3$), 0.94 (s, 9H, silyl ether $(CH_3)_3$), 0.11 (s, 6H, 2 × CH_3)

^{13}C NMR (100 MHz, $CDCl_3$) δ 181.9, 167.3, 164.2, 147.6, 137.9, 137.8, 136.1, 134.4, 105.6, 101.9, 99.8, 59.8, 36.0, 35.5, 27.9, 27.8, 25.9, 18.4, –5.2.

Step 3: Synthesis of compound (27)

To a solution of the aldehyde prepared in step 2 (167 mg, 0.362 mmol) and 2-cyanoacetic acid (48 mg; 0.565 mmol) in chlorofom (30 mL) was added piperidine (243 μL; 2.4 mmol). The mixture was refluxed for 20 hours under argon atmosphere and then cooled down to room temperature. The solvent was then evaporated under reduced pressure. Pure compound was obtained by reverse C18 column chromatography (CH_3CN/NH_4AcO (20 mM), 1/1). Before evaporation of the solvent, a drop of diluted AcOH was added in all fractions. Yield: 120 mg, 62%, purple solid.

^{1}H NMR (400 MHz, $CDCl_3$) δ 8.20 (s, 1H), 7.65 (s, 1H), 6.75 (brs, 1H), 5.89 (m, 2H), 4.70 (s, 2H), 1.31 (s, 9H), 1.24 (s, 9H), 0.94 (s, 9H), 0.11 (s, 6H)

^{13}C NMR (100 MHz, $CDCl_3$) δ 169.8, 168.8, 165.5, 150.9, 146.5, 140.2, 138.5, 136.5, 129.1, 117.1, 106.2, 102.5, 100.8, 90.7, 59.6, 36.2, 35.7, 27.8, 25.9, 18.3, –5.2.

Scheme synthesis of Compound (30): (3-cyano-4-(2-(5-((2,6-diphenyl-4*H*-pyran-4-ylidene)methyl)-3,4-dihexylthiophen-2-yl)vinyl)-5,5-dimethylfuran-2(5H)-ylidene) malononitrile

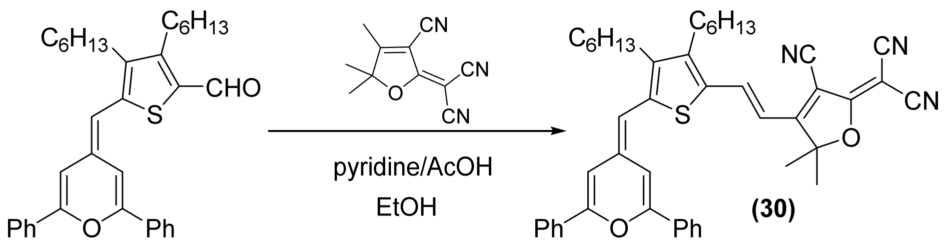

To a solution of the described aldehyde[1] (289.2 mg, 0.55 mmol) in ethanol (10 mL) 2-(3-cyano-4,5,5-trimethylfuran-2(5*H*)-ylidene)malononitrile[22] (100 mg, 0.55 mmol), pyridine (440 μL, 5.40 mmol) and acetic acid (217 μL, 3.78 mmol) were added and the mixture was refluxed under argon atmosphere for 24 h. The reaction was stirred at 0 °C for 1 h, resulting in the formation of a precipitate, which was filtered and washed with cold hexane and a mixture of hexane/CH_2Cl_2 (9:1). Yield: 172.3 mg, 44% of a dark red solid.

M.p.: 210-212 °C

^{1}H NMR (300 MHz, $CDCl_3$) δ 8.07 (d, *J* = 15.0 Hz, 1H, thiophene-C**H**=), 7.98 – 7.76 (m, 4H, H's Ar.), 7.64 – 7.43 (m, 6H, H's Ar.), 7.40 (d, *J* = 2.0 Hz, 1H, H pyranylidene), 6.68 (d, *J* = 2.0 Hz, 1H, H pyranylidene), 6.41 (d, *J* = 15.0 Hz, 1H, **H**C=acceptor), 6.18 (s, 1H, H pyranylidene exocyclic), 2.70 (t, J = 7.7 Hz, 2H, CH_2-thiophene), 2.62 (t, J = 8.8 Hz, 2H, CH_2-thiophene), 1.73 (s, 6H, 2 × CH_3), 1.65 – 1.20 (m, 16H, H's hexyl chain), 0.91 (t, *J* = 6.5 Hz, 6H, 2 × CH_3 hexyl chain).

^{13}C NMR (75 MHz, $CDCl_3$) *δ* 176.5, 172.3, 156.7, 154.0, 153.4, 148.7, 142.1, 136.9, 135.4, 132.3, 132.1, 132.0, 130.8, 130.2, 129.2, 129.0, 125.7, 125.0, 113.2, 112.5, 112.4, 110.1, 108.6, 106.5, 104.0, 96.1, 78.4, 31.9, 31.7, 31.6, 30.5, 29.6, 29.6, 28.0, 27.2, 26.8, 22.7, 22.6, 14.1, 14.1.

HRMS (ESI$^+$): m/z calculated for $C_{46}H_{48}N_3O_2S$ $[M+H]^{+\cdot}$ 706.3462; found 706.3413

Scheme synthesis of Compound (34): 3-(2,6-diphenyl-4*H*-pyran-4-ylidene)-2-phenylprop-1-ene-1,1,3-tricarbonitrile

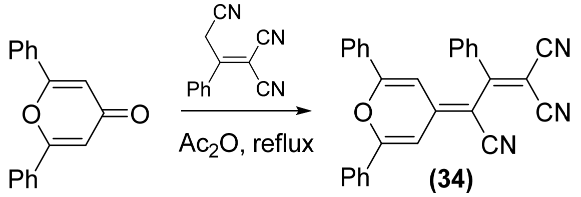

(34)

To a solution of 2,6-diphenyl-4H-pyran-4-one[23] (300 mg, 1.208 mmol) in acetic anhydride (1.50 mL) was added 2-phenylprop-1-ene-1,1,3-tricarbonitrile[24] (235 mg, 1.21 mmol) and the mixture was refluxed under an argon atmosphere for 1 h 30 min. The reaction was cooled to room temperature and then to 0 °C for 1 h. The precipitate was filtered a washed three times with cold ether. Yield: 357 mg, 70%, light pink solid.

^{1}H NMR (400 MHz, $CDCl_3$, 45 °C) δ (ppm): 7.99-7.85 (m, 2H, H's Ar.), 7.64-7.43 (m, 14H, H's Ar. + H pyranylidene), 6.53 (d, J = 2.0 Hz, 1H, H pyranylidene)

^{13}C NMR (100 MHz, $CDCl_3$, 45 °C) δ 167.3, 161.4, 160.2, 152.5, 135.1, 133.0, 132.5, 132.4, 130.8, 130.7, 130.4, 129.8, 129.7, 129.6, 126.4, 126.4, 117.3, 114.6, 113.8, 88.4, 79.0

Scheme synthesis of Compound (35): 2-phenyl-3-(1,2,6-trimethylpyridin-4(1*H*)-ylidene)prop-1-ene-1,1,3-tricarbonitrile

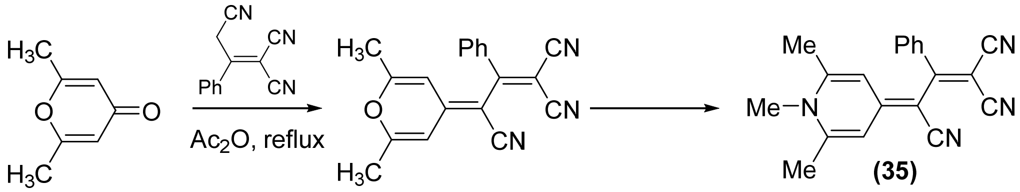

(35)

Step 1:

To a solution of 2,6-dimethyl-4*H*-pyran-4-one (commercially available) (117 mg, 0.941 mmol) in acetic anhydride (1.16 mL) was added 2-phenylprop-1-ene-1,1,3-tricarbonitrile[24] (181 mg, 0.937 mmol) and the mixture was refluxed under argon atmosphere for 1 h. The crude of the reaction was extracted with a mixture of CH_2Cl_2/H_2O and the organic phase washed with H_3PO_4 (2%, 30 mL), NaOH (10%, 30 mL), saturated NaCl (30 mL) and the solvent was evaporated under reduced pressure. Yield: 265 mg, 94%.

^{1}**H NMR** (400 MHz, $CDCl_3$, 45 °C) δ (ppm): 7.62-7.46 (m, 5H, H's Ar.), 6.79 (d, J = 2.2 Hz, 1H, H pyranylidene), 5.96 (d, J = 2.2 Hz, 1H, H pyranylidene), 2.36 (s, 3H, CH_3), 2.25 (s, 3H, CH_3)

Step 2:

To a solution of the product prepared in step 1 (260 mg, 0.869 mmol) in ethanol (2 mL) a solution of dimethylamine (870 μL of a 40% solution in H_2O) was added. The mixture of reaction was refluxed for 45 min (the solution is dark red) and then cooled to 0 °C. The red precipitate was filtered, washed with 0.5 mL of cold ethanol and dried. Yield: 111 mg, 41% of a red solid.

^{1}**H NMR** (400 MHz, $CDCl_3$) δ (ppm): 7.57-7.42 (m, 5H, H's Ar.), 6.91 (s, 2H, H pyranylidene), 3.72 (s, 3H, N-CH_3), 2.51 (s, 6H, 2 × CH_3)

^{13}**C NMR** (100 MHz, $CDCl_3$) δ (ppm) δ 167.2, 152.8, 149.3, 136.4, 131.6, 130.4, 129.0, 120.3, 120.1, 117.9, 116.9, 80.1, 62.6, 56.8, 37.2, 21.6.

MS (MALDI): m/z 313.149 $[M+H]^+$

Scheme synthesis of Compound (36): 2-cyano-3-(5'-((2,6-diphenyl-4*H*-pyran-4-ylidene)methyl)-[2,2'-bithiophen]-5-yl)acrylic acid

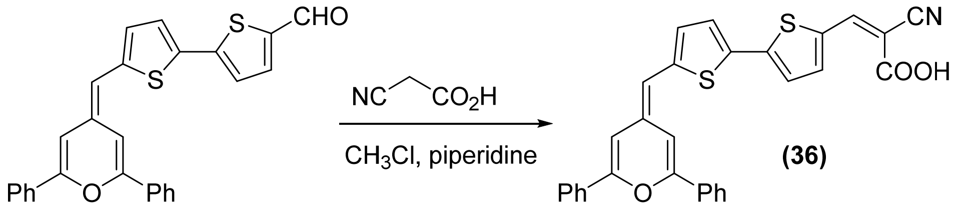

To a solution of 5′-[(2,6-diphenyl-4*H*-pyran-4-ylidene)methyl]-2,2′-bithiophene-5-carbaldehyde[12] (150 mg, 0.34 mmol) and 2-cyanoacetic acid (47 mg, 0.53 mmol) in chloroform (6.5 mL) was added dropwise piperidine (230 μL, 2.26 mmol), and the mixture was refluxed under argon atmosphere for 72 h. The reaction mixture was cooled to room temperature and then to 0 °C. The precipitate formed was filtered, washed with a hexane/CH_2Cl_2 mixture (98:2) and dried. Yield: 67 mg, 39% of a dark purple solid.

IR (KBr): cm^{-1} 2217 (C≡N), 1686 (C=O).

^{1}**H NMR (400 MHz, DMSO-d_6):** δ 8.38 (s, 1H, **H**C=C(CN)COOH), 8.06–7.81 (m, 5H, H's Ar.), 7.67–7.42 (m, 8H, H's Ar. + H's thiophene), 7.16 (d, *J* = 4.1 Hz, 1H, H

thiophene), 7.11 (s, 1H, H pyranylidene), 6.84 (s, 1H, H pyranylidene), 6.26 (s, 1H, H pyranylidene exocyclic).

^{13}C NMR (100 MHz, DMSO-d$_6$): Due to the extreme insolubility of the product, the ^{13}C NMR spectrum could not be recorded.

HRMS (ESI$^+$): *m/z* Calculated for $C_{30}H_{19}NO_3S_2$ $[M]^+$ 505.0801; found 505.0785.

Scheme synthesis of Compound (37), Compound (38) and Compound (39)

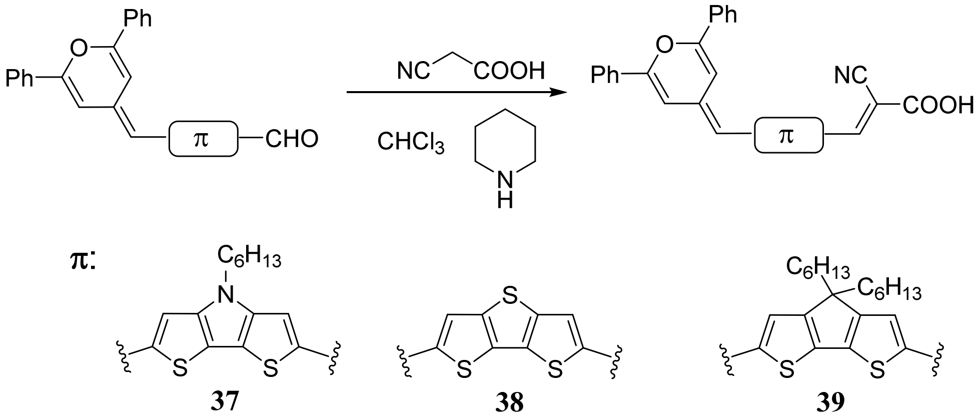

Compound (37): 2-cyano-3-(6-((2,6-diphenyl-4*H*-pyran-4-ylidene)methyl)-4-hexyl-4*H*-dithieno[3,2-*b*:2′,3′-*d*]pyrrol-2-yl)acrylic acid

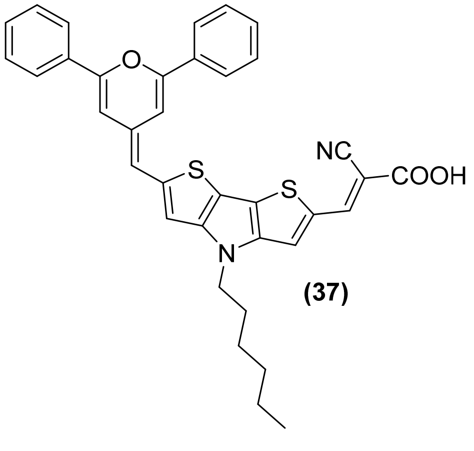

(37)

To a solution of *N*-hexyl-6-(2,6-diphenyl-4*H*-pyran-4-ylidenemethyl)dithieno[3,2-*b*:2′,3′-*d*]pyrrole-2-carbaldehyde[12] (0.069 g, 0.130 mmol) and cyanoacetic acid (0.016 g, 0.19 mmol) in chloroform (4 mL) under an argon atmosphere, piperidine (85 μL, 0.85 mmol) was added. The mixture was refluxed for 48 h and then cooled in an ice bath. The resulting solid was filtered, washed with cold hexane, and

subsequently washed with an aqueous solution of 0.1 N HCl and water. The product was obtained as a dark purple solid (0.058 g, 0.096 mmol, 75%).

M.p.: (°C): 174–176. **IR** (KBr): $\bar{\nu}$ (cm^{-1})3429 (O-H), 2209 (C≡N), 1652 (C=O), 1570 (C=C), 1527 (C=C).

^{1}H NMR (300 MHz, DMSO-d_6, 40 °C, presat. water) δ (ppm): 8.39 (s, 1H, **H**C=C(CN)COOH), 8.06 (s, 1H, H thiophene), 8.01–7.82 (m, 4H, H's Ar.), 7.69–7.41 (m, 6H, H's Ar.), 7.28 (s, 1H, H thiophene), 7.24 (s, 1H, H pyranylidene), 6.92 (s, 1H, H pyranylidene), 6.34 (s, 1H, H pyranylidene exocyclic), 4.38–4.24 (m, 2H, N-CH_2), 1.91–1.74 (m, 2H, N-CH_2-**CH_2**), 1.36–1.15 (m, 6H, 3 × CH_2 hexyl chain), 0.87–0.74 (m, 3H, CH_3).

^{13}C NMR: not registered due to the poor solubility of the product.

HRMS (ESI$^+$): m/z calculated for $C_{36}H_{30}N_2O_3S_3$ [$M^{+\cdot}$] 602.1692; found 602.1640.

Compound (38): 2-cyano-3-(6-((2,6-diphenyl-4*H*-pyran-4-ylidene)methyl)dithieno- [3,2-*b*:2′,3′-*d*]thiophen-2-yl)acrylic acid

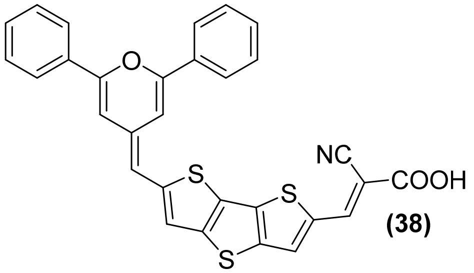

(38)

To a solution of 6-(2,6-diphenyl-4*H*-pyran-4-ylidenemethyl)dithieno[3,2-*b*:2′,3′-*d*]thiophene-2-carbaldehyde[25] (0.050 g, 0.14 mmol) and cyanoacetic acid (0.014 g, 0.17 mmol) in chloroform (3.5 mL) under an argon atmosphere, piperidine (80 µL, 0.80 mmol) was added. The mixture was refluxed for 48 h, then cooled in an ice bath. The resulting solid was collected by filtration, washed successively with a cold mixture of hexane/dichloromethane (8:2), an aqueous solution of 0.1 N HCl, and water. The product was obtained as a purple solid (0.032 g, 0.060 mmol, 56%).

M.p.: (°C): 215–217. **IR** (KBr): $\bar{\nu}$ (cm^{-1}) 3444 (O-H), 2213 (C≡N), 1652 (C=O), 1570 (C=C).

^{1}H NMR (500 MHz, DMSO-d_6, 77ºC, presat. water) δ (ppm): 8.51 (s, 1H, **H**C=C(CN)COOH), 8.33 (s, 1H, H thiophene), 8.03–7.95 (m, 2H, H's Ar.), 7.93–7.85 (m,

2H, H's Ar.), 7.64–7.45 (m, 7H, H's Ar.+ H thiophene), 7.18 (s, 1H, H pyranylidene), 6.92 (s, 1H, H pyranylidene), 6.34 (s, 1H, H pyranylidene exocyclic).

^{13}C NMR: not registered due to the poor solubility of the product.

HRMS (MALDI$^+$): m/z calculated for $C_{30}H_{17}NO_3S_3$ [$M^{+\cdot}$] 535.0365; found 535.0439.

Compound (39): 2-cyano-3-(6-((2,6-diphenyl-4*H*-pyran-4-ylidene)methyl)-4,4-dihexyl-4*H*cyclopenta[2,1-*b*:3,4-*b'*]dithiophen-2-yl)acrylic acid

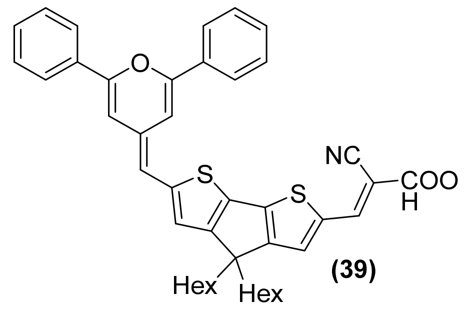

(39)

To a solution of 4,4-dihexyl-6-(2,6-diphenyl-4*H*-pyran-4-ylidenemethyl)-cyclopenta[2,1-*b*:3,4-*b'*]dithiophene-2-carbaldehyde[12] (0.061 g, 0.098 mmol) and cyanoacetic acid (0.013 g, 0.15 mmol) in chloroform (3 mL) under argon atmosphere, piperidine (65 μL, 0.65 mmol) was added. The mixture was refluxed for 87 h and then cooled in an ice bath. The resulting solid was filtered and washed with hexane, followed by an aqueous solution of 0.1 N HCl and water. The product was obtained as a dark blue solid (0.034 g, 0.049 mmol, 50%).

M.p.: (°C): 128–130. IR (KBr): $\bar{\nu}$ (cm^{-1}) 3433 (O-H), 2209 (C≡N), 1654 (C=O), 1570 (C=C).

^{1}H NMR (400 MHz, CD_2Cl_2) δ (ppm):

8.28 (s, 1H, **H**C=C(CN)COOH), 7.95–7.89 (m, 2H, H's Ar.), 7.84–7.78 (m, 2H, H's Ar.), 7.61–7.34 (m, 7H, H's Ar.+ H thiophene), 7.22 (d, *J* = 1.4 Hz, 1H, H pyranylidene), 6.86 (s, 1H, H thiophene), 6.54 (d, *J* = 1.4 Hz, 1H, H pyranylidene), 6.20 (s, 1H, H pyranylidene exocyclic), 1.97–1.81 (m, 4H, 2 × C-CH_2), 1.23–1.08 (m, 12H, CH_2 of hexyl chains), 1.03–0.92 (m, 4H, CH_2 of hexyl chains), 0.85–0.77 (m, 6H, 2 × CH_3 hexyl chain).

^{13}C NMR: not registered due to the poor solubility of the product.

HRMS (ESI⁺): m/z calculated for $C_{43}H_{43}NO_3S_2$ [M^{+}] 685.2684; found 685.2697; calculated for $C_{43}H_{44}NO_3S_2$ $[M+H]^+$: 686.2757; found 686.2689.

Scheme synthesis of Compound (42): 2-cyano-4-(2,6-di-tert-butyl-4*H*-pyran-4-ylidene)but-2-enoic acid

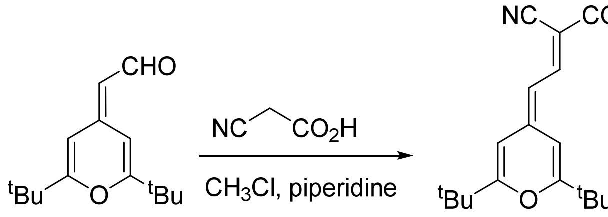

(42)

To a solution of 2-(2,6-di-tert-butyl-4*H*-pyran-4-ylidene)acetaldehyde[17] (112 mg, 0.48 mmol) and cyanoacetic acid (63.6 mg, 0.748 mmol) in chloroform (15 mL) under argon atmosphere, piperidine (317 μL, 3.18 mmol). The mixture was refluxed for 20 h and the solvent evaporated under reduced pressure. Pure compound was obtained by reverse C18 column chromatography (MeOH/NH_4AcO (20 mM), 3/7). Before evaporation of the solvent, a drop of 10% AcOH was added in all fractions. Yield: 116 mg, 80%, orange solid.

M.p: 203-204 °C

^{1}H NMR: (400 MHz, $CDCl_3$) δ 1.27 (s, 18H), 1.30 (s, 18H), 6,0 (d, *J* = 6 Hz, 1H), 6,3 (dd, *J* = 6Hz, *J* = 6.3Hz, 2H), 8.25 (d, *J* = 8.3Hz, 1H)

^{13}C NMR: (100 MHz, THF) δ 25.2, 25.3, 98.4, 104.6, 105.8, 146.9

HRMS (ESI⁺) m/z: Calculated for $C_{18}H_{23}O_3N$: 301.1672 found: 301.1152

Scheme synthesis of Compound (46): 3-(4-(((*tert*-butyldiphenylsilyl)oxy)methyl)-5-((2,6-di-tert-butyl-4*H*-pyran-4-ylidene)methyl)thiophen-2-yl)-2-cyanoacrylic acid

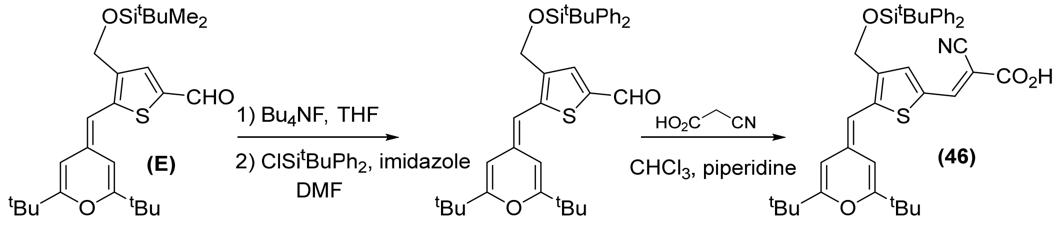

Step 1: To a solution of aldehyde **E** (see above synthesis of Compound 27) (188 mg, 0.41 mmol) in anhydrous THF (10 mL) was added Bu_4NF (1.0 M in THF) (0.815 mL,

0.815 mmol). The mixture was stirred at 0 °C under an argon atmosphere for 1h. Subsequently, the solvent was evaporated under reduced pressure, and the resulting crude product was used directly in step 2 without further purification.

The crude was dissolved in anhydrous DMF (8 mL) and imidazole (140.7 mg, 0.857 mmol) and $ClSi^tBuPh_2$ (0.129 mL, 0.49 mmol) were added. The mixture was stirred at room temperature overnight and then 30 mL of water was added. The solution was extracted with ethyl acetate (3 × 15 mL), washed with saturated NaCl and the organic phase dried over $MgSO_4$. The pure compound was obtained by silica gel column chromatography (10% ethyl acetate in hexanes). Yield: 141 mg, 60%).

^{1}H NMR (400 MHz, CD_2Cl_2) δ 9.71 (s, 1H, CHO), 7.75-7.62 (m, 4H, H's Ar.), 7.60 (s, 1H, H thiophene), 7.51-7.34 (m, 6H, H's Ar.), 6.51 (d, *J* = 1.9 Hz, 1H, H pyranylidene), 5.72-5.70 (m, 2H, H pyranylidene + H pyranylidene exocyclic), 4.73 (s, 2H, OCH_2), 1.27 (s, 9H, $(CH_3)_3$), 1.20 (s, 9H, $(CH_3)_3$), 1.07 (s, 9H, silyl ether $(CH_3)_3$)

^{13}C NMR (100 MHz, CD_2Cl_2) δ 182.0, 167.7, 164.6, 148.0, 138.3, 137.6, 136.6, 136.0, 134.8, 133.7, 130.2, 128.2, 105.9, 102.2, 100.1, 36.3, 35.8, 28.0, 27.9, 27.0, 19.5

Step 2: To a solution of the aldehyde obtained in step 1 (106 mg, 0.181 mmol) and cyanoacetic acid (24.2 mg, 0.284 mmol) in chloroform (15 mL) under an argon atmosphere, piperidine (132 μL, 1.20 mmol). The reaction mixture was refluxed for 20 h, after which the solvent was removed under reduced pressure. The pure compound was isolated by reverse C18 column chromatography (MeOH/NH_4AcO (20 mM), 1/1). Before solvent removal, a drop of 10% AcOH was added in all collected fractions. Yield: 71.5 mg, 60% as a purple solid.

^{1}H NMR (400 MHz, TFH-d8) δ 8.21 (s, 1H, **H**C=C(CN)COOH), 7.76-7.69 (m, 5H, H's Ar.+ H thiophene), 7.44-7.37 (m, 6H, H's Ar.), 6.74 (br s, 1H, H pyranylidene), 5.88-5.84 (m, 2H, H pyranylidene + H pyranylidene exocyclic), 4.78 (s, 2H, OCH_2), 1.30 (s, 9H, $(CH_3)_3$), 1.22 (s, 9H, $(CH_3)_3$), 1.08 (s, 9H, silyl ether $(CH_3)_3$)

^{13}C NMR (100 MHz, THF-d8) δ 168.5, 165.2, 165.1, 148.8, 146.0, 140.0, 138.6, 138.3, 136.6, 135.4, 134.3, 131.0, 130.8, 128.8, 126.1, 117.7, 107.0, 103.7, 101.3, 95.7, 61.3, 37.0, 36.4, 35.3, 28.3, 28.3, 27.4.

Spectral characterization data for new synthesized compounds

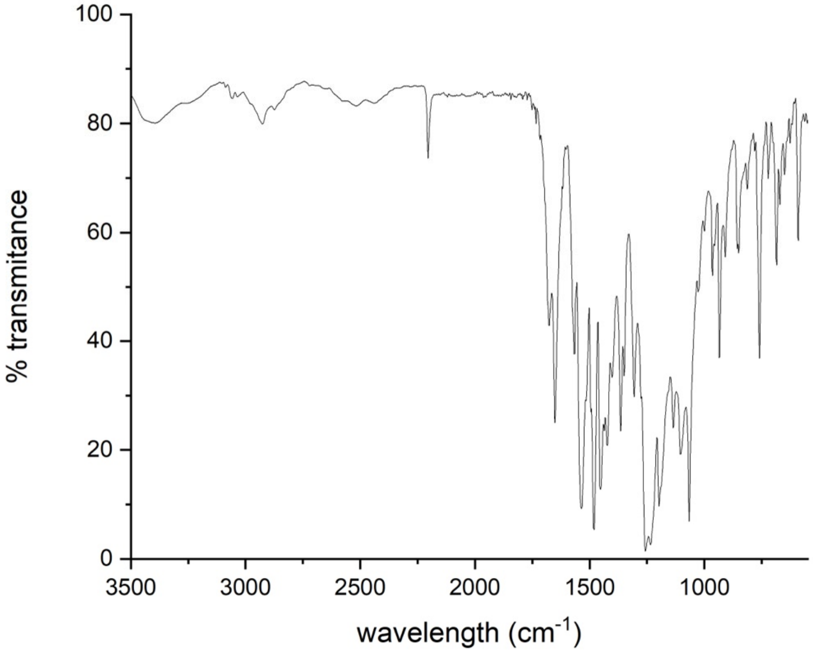

Figure S2. IR spectrum (KBr) of compound 3.

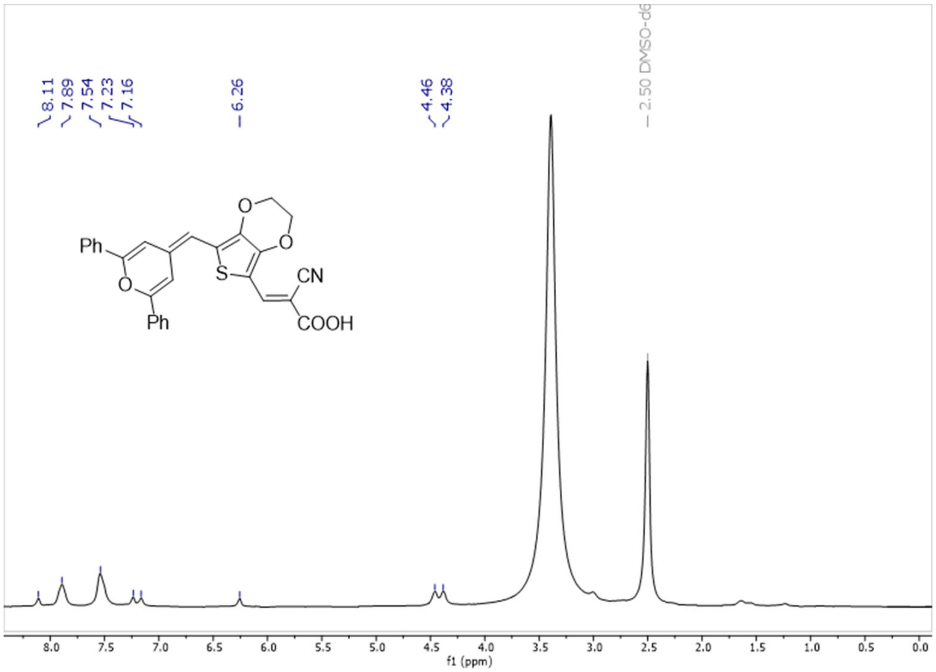

Figure S3. ¹H NMR spectrum of compound 3 (400 MHz, DMSO-d$_6$).

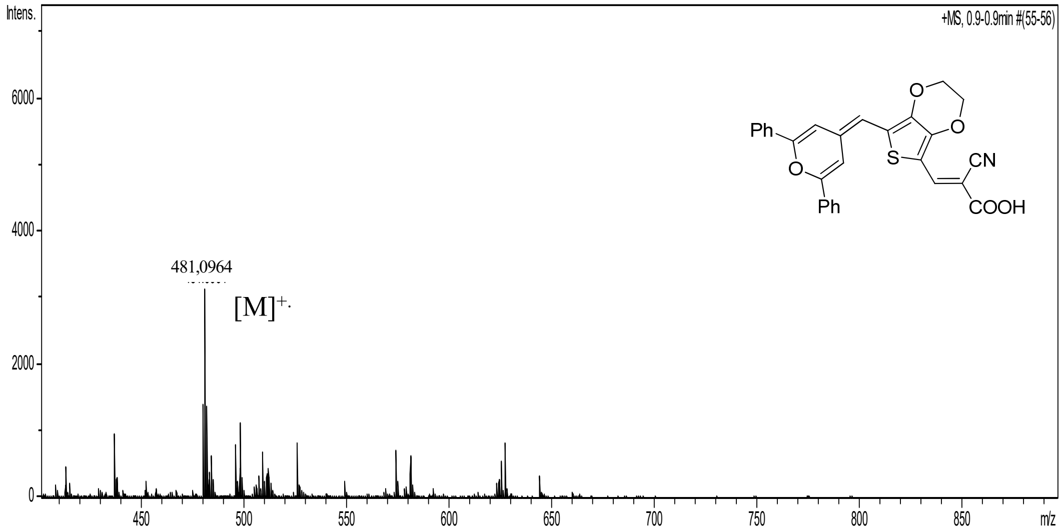

Fig. S4. HRMS (ESI$^+$) spectrum of compound 3.

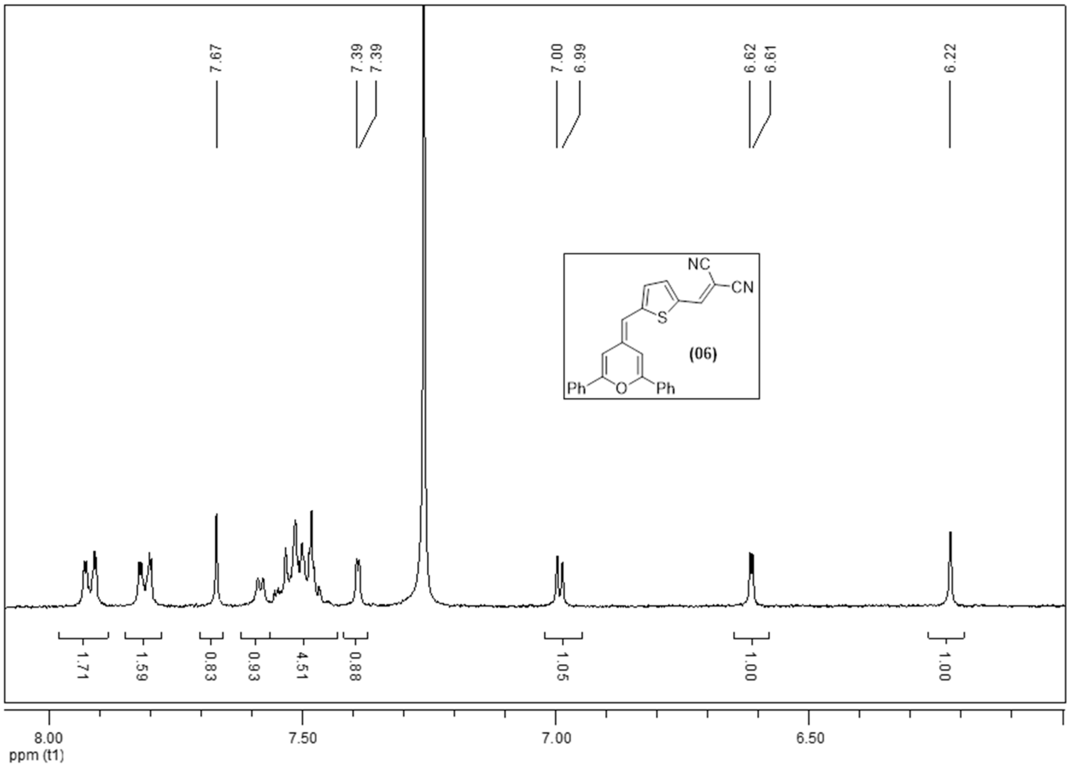

Figure S5. ^{1}H NMR spectrum of compound 6 (400 MHz, $CDCl_3$).

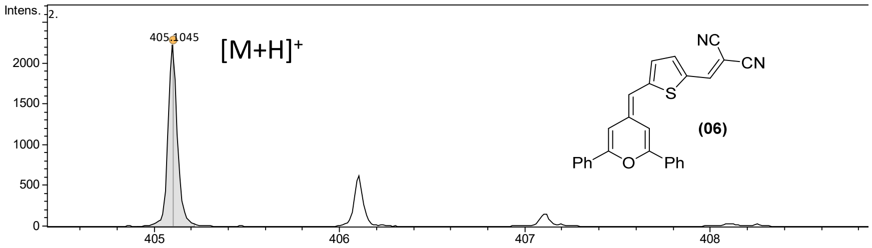

Figure S6. HRMS (ESI+) spectrum of compound 6.

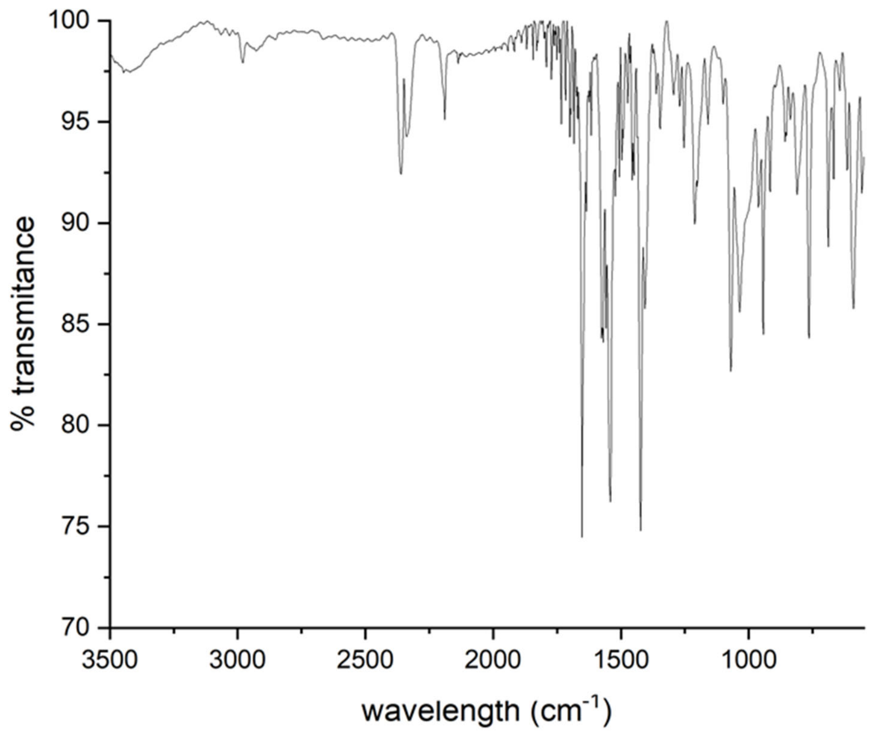

Figure S7. IR spectrum (KBr) of compound 7

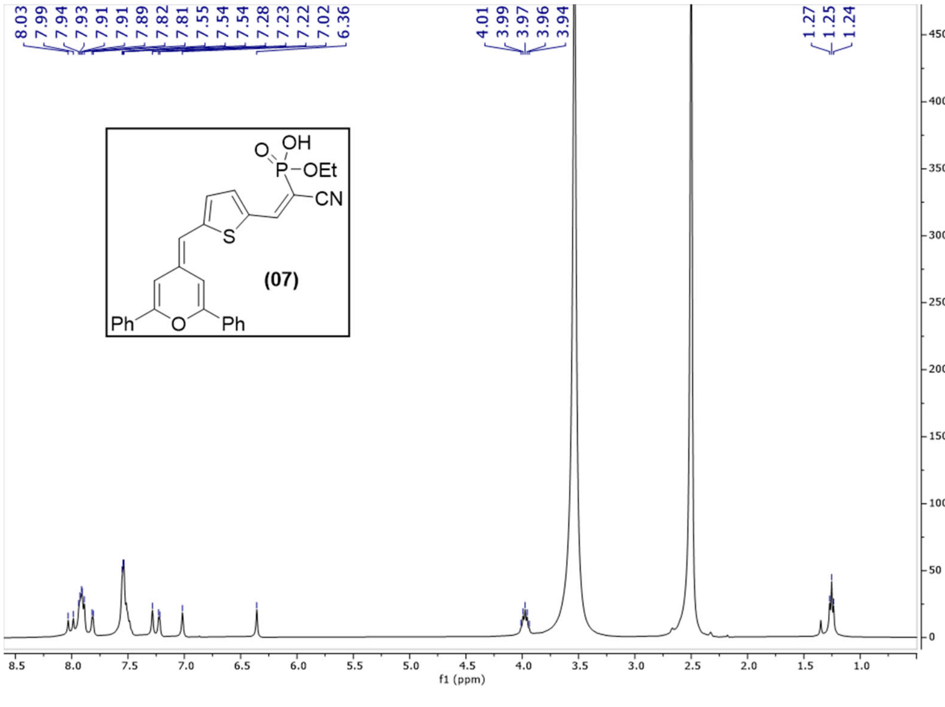

Figure S8. ^{1}H NMR spectrum of compound 7 (400 MHz, DMSO-d6).

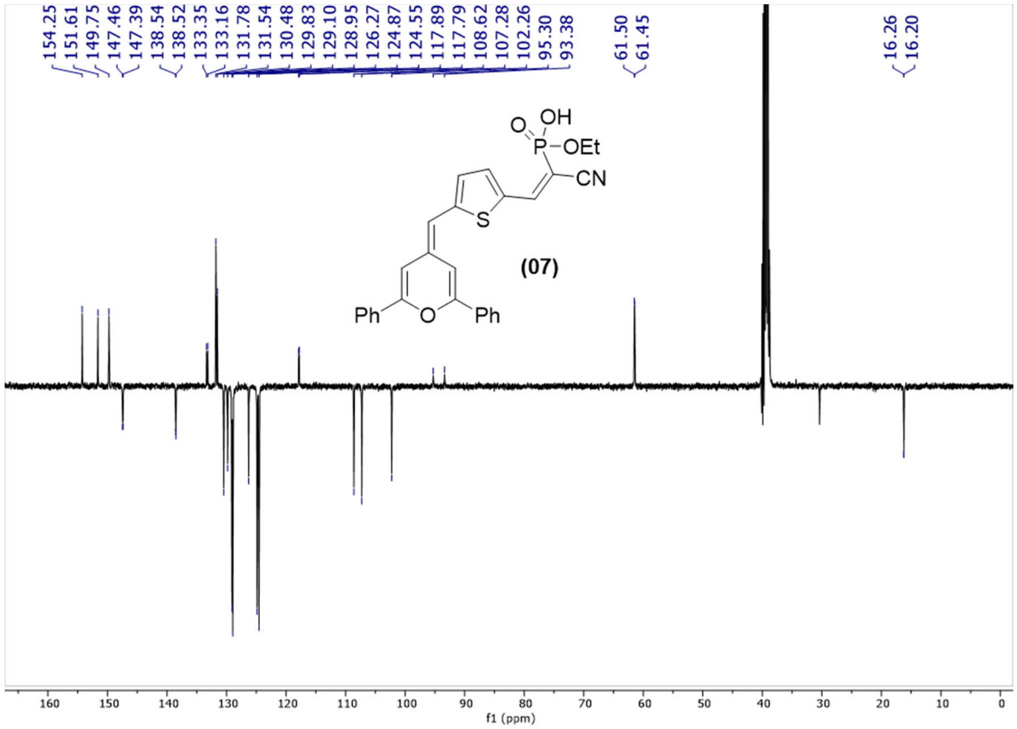

Figure S9. ^{13}**C NMR (APT) spectrum of compound 7 (100 MHz, DMSO-d6).**

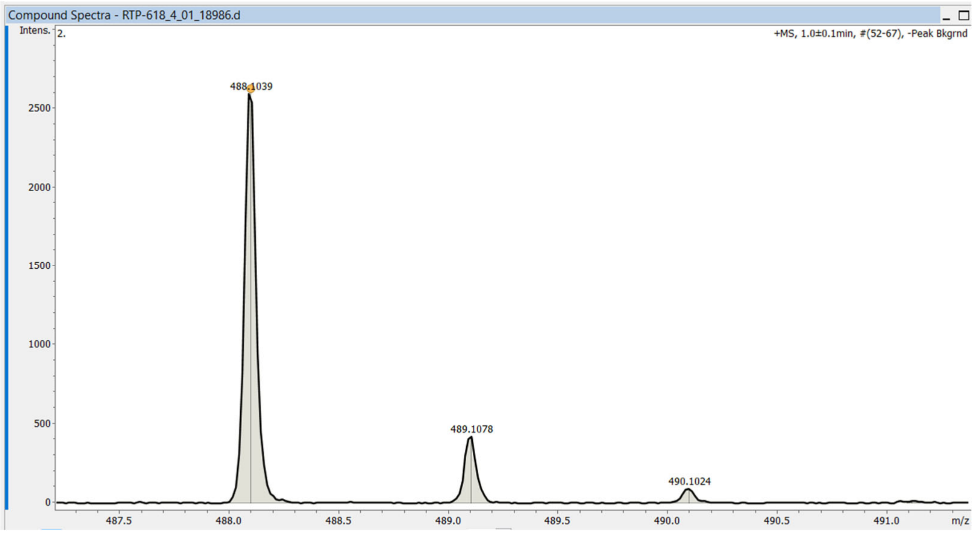

Fig. S10. HRMS (ESI$^+$) spectrum of compound 7

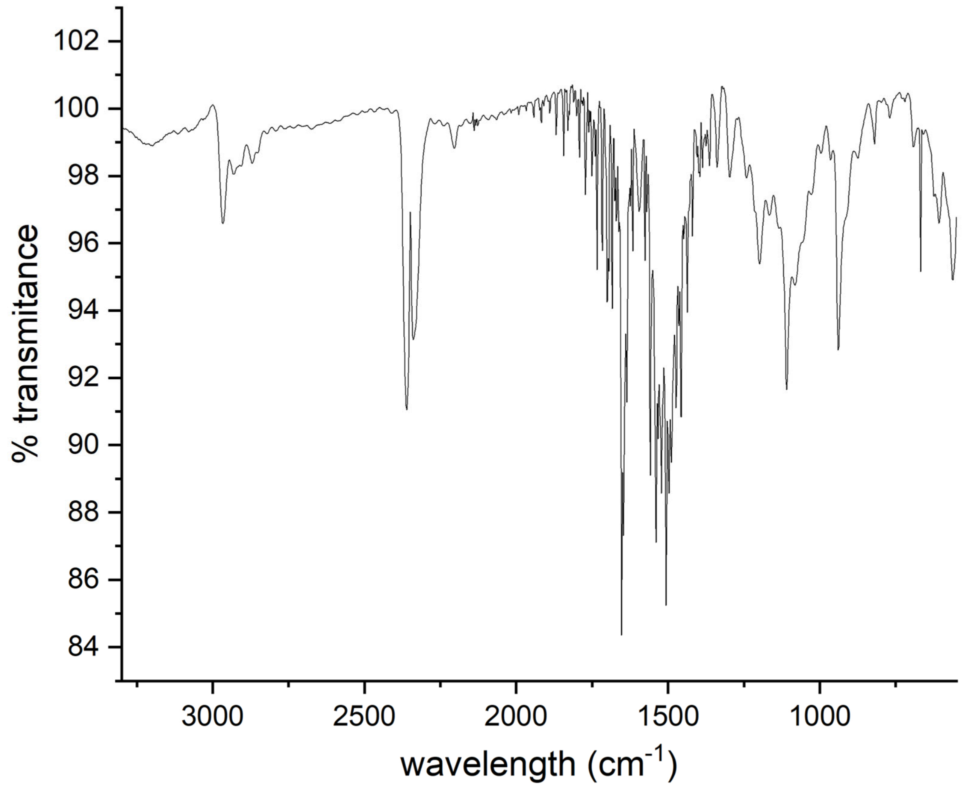

Figure S11. IR spectrum (KBr) of compound 10.

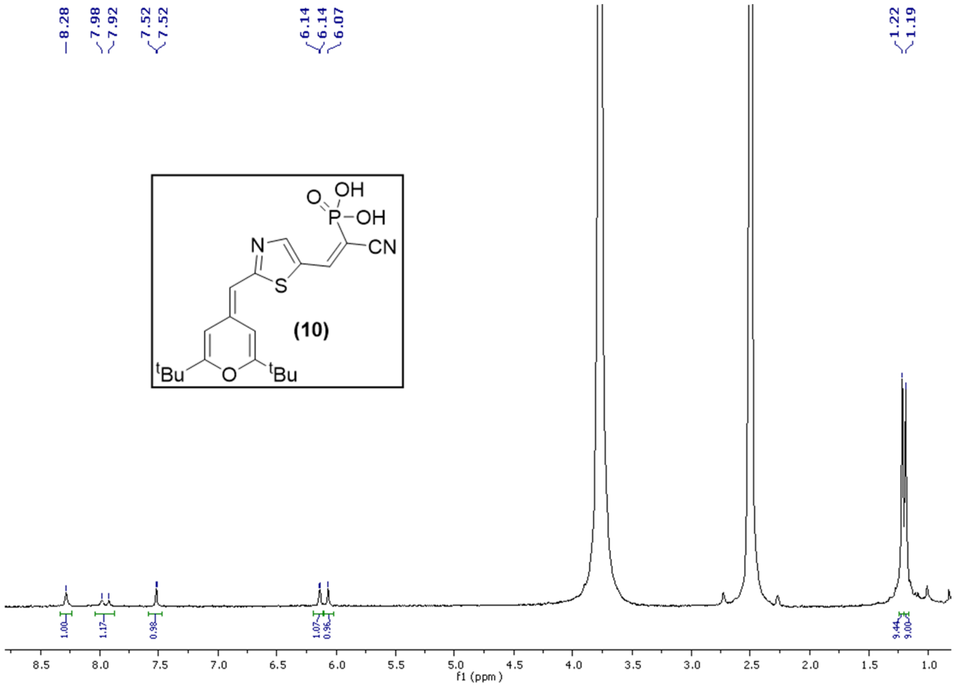

Figure S12. ^{1}H NMR spectrum of compound 10 (400 MHz, DMSO-d6).

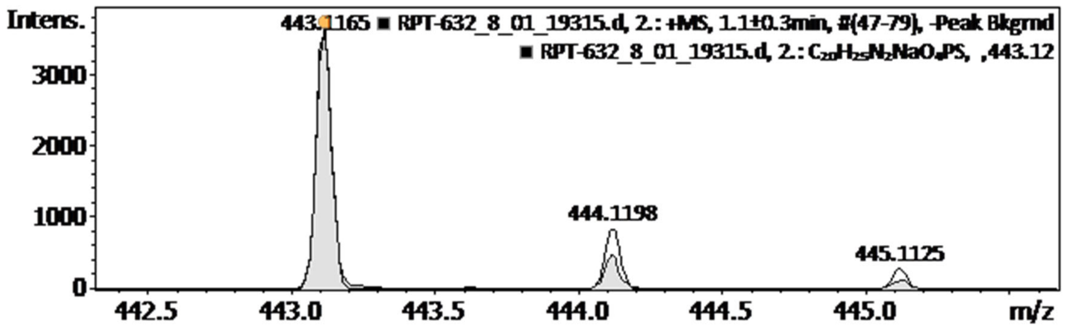

Fig. S13. HRMS (ESI+) spectrum of compound 10

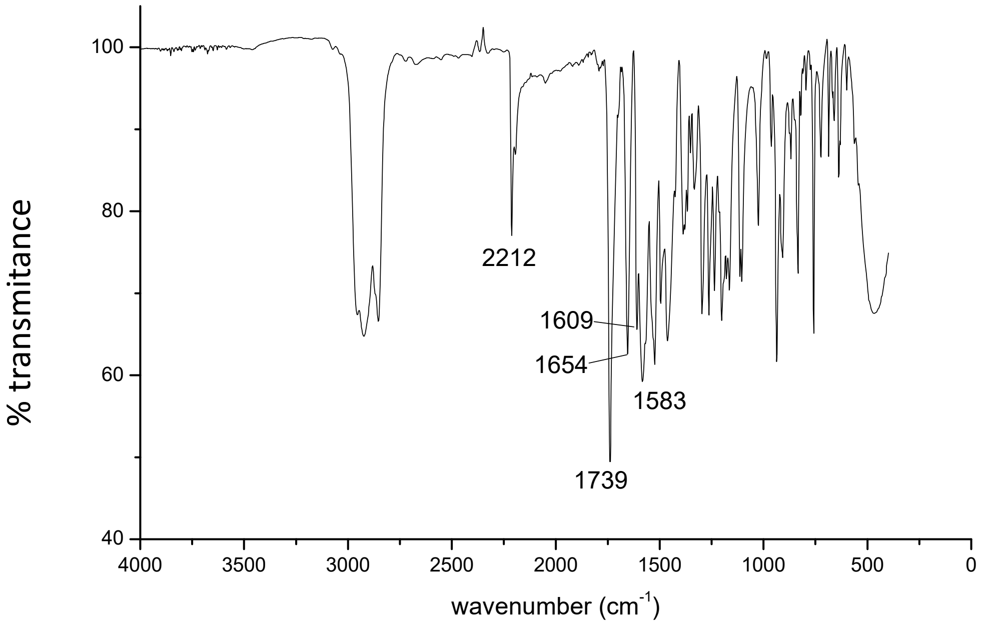

Figure S14. IR spectrum (nujol) of compound 13.

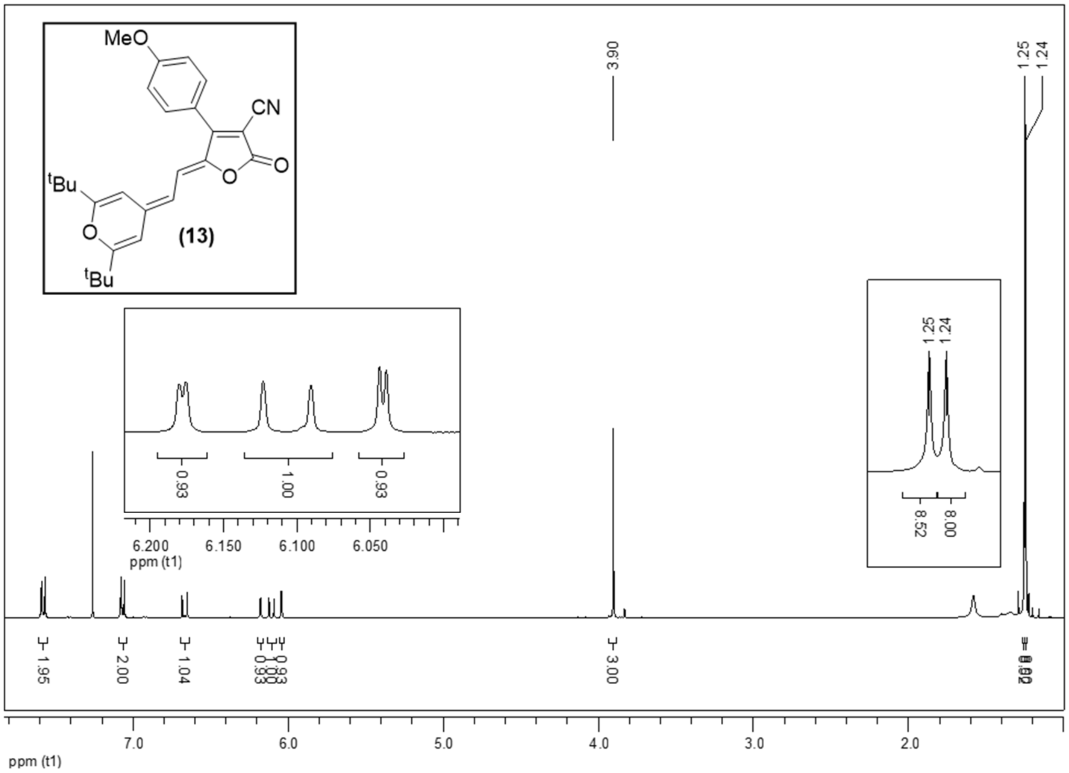

Figure S15. **^{1}H NMR spectrum of compound 13 (400 MHz, $CDCl_3$).**

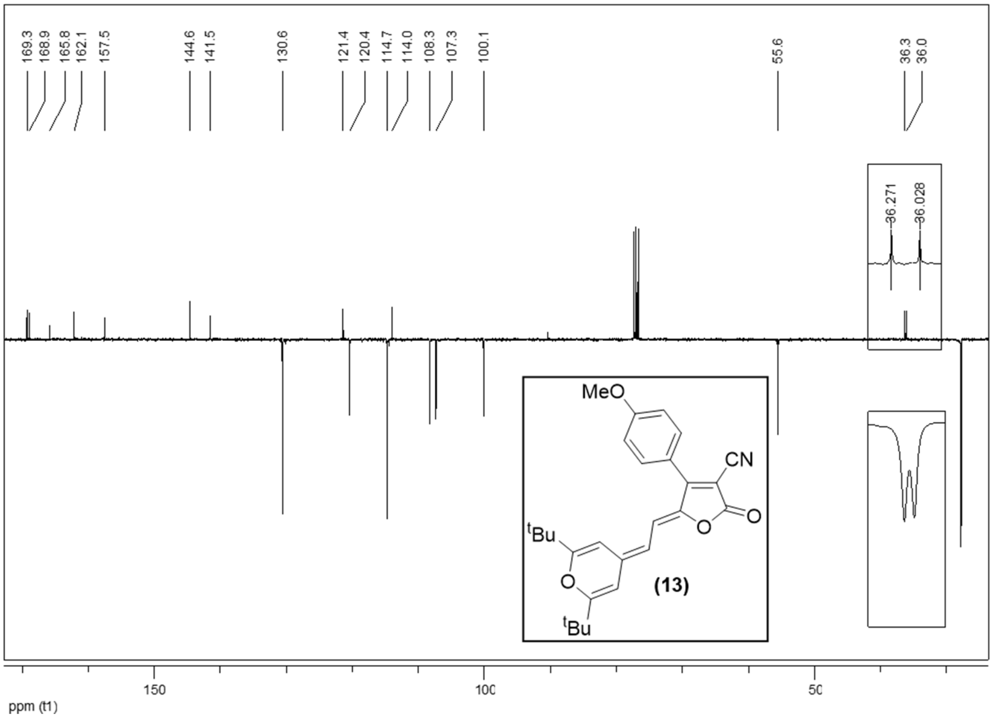

Figure S16. **^{13}C NMR (APT) spectrum of compound 13 (100 MHz, $CDCl_3$).**

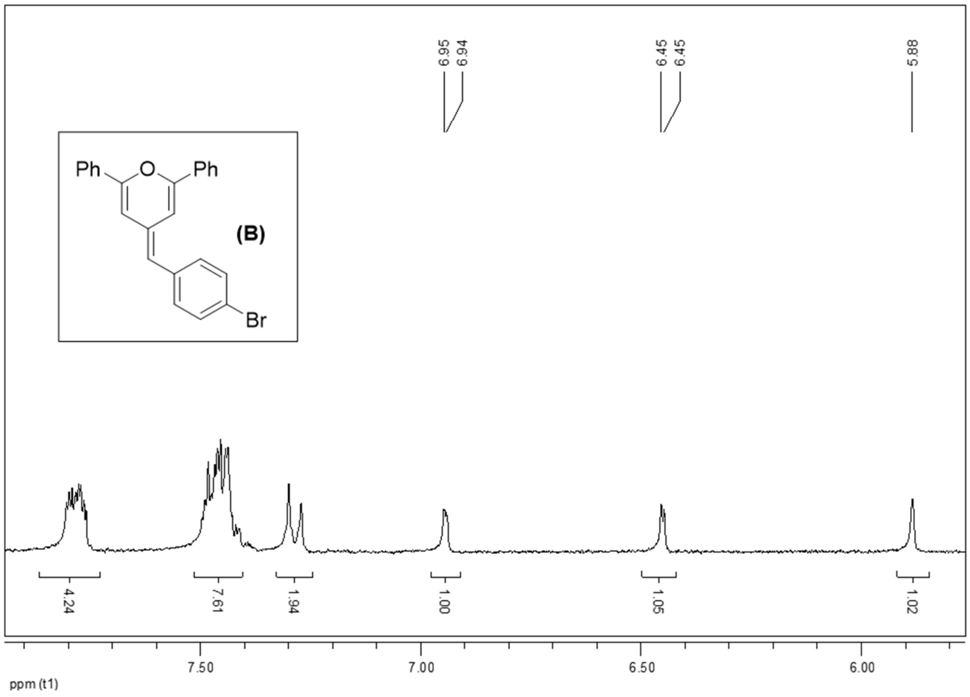

Figure S17. ^{1}H NMR spectrum of compound B (300 MHz, CH_2Cl_2).

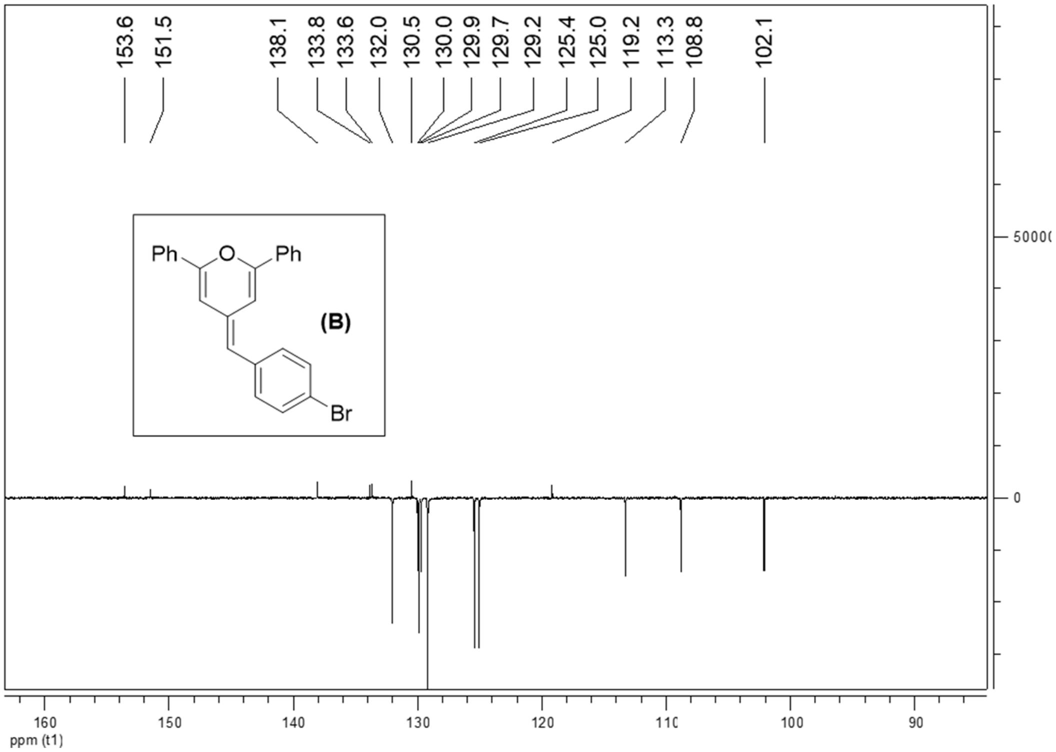

Figure S18. ^{13}C NMR (APT) spectrum of compound B (100 MHz, CH_2Cl_2).

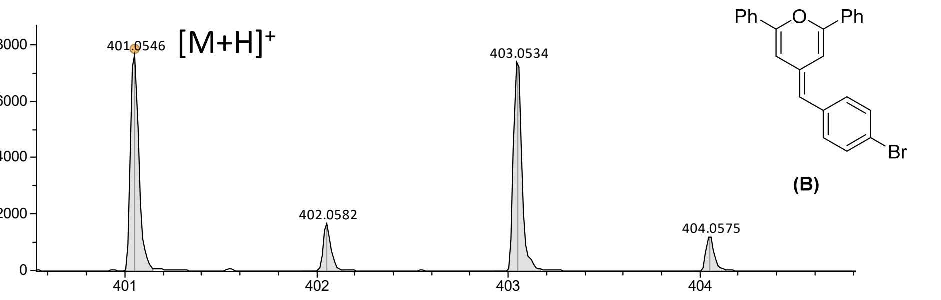

Figure S19. HRMS (ESI+) spectrum of compound B.

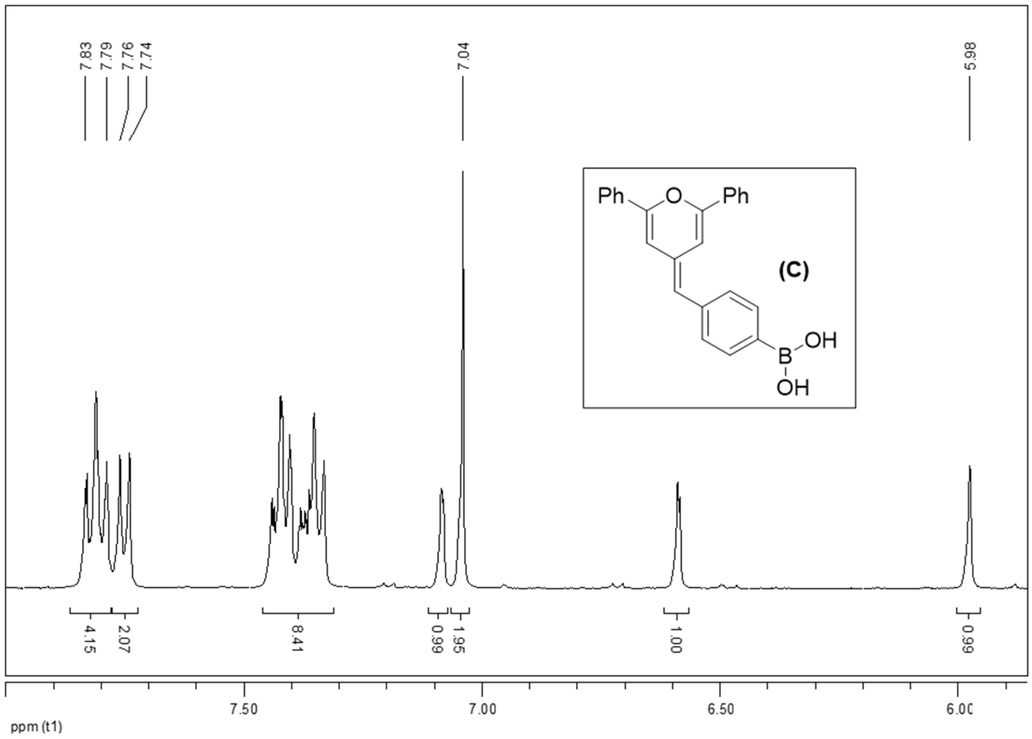

Figure S20. 1H NMR spectrum of compound C (400 MHz, TFH-d8).

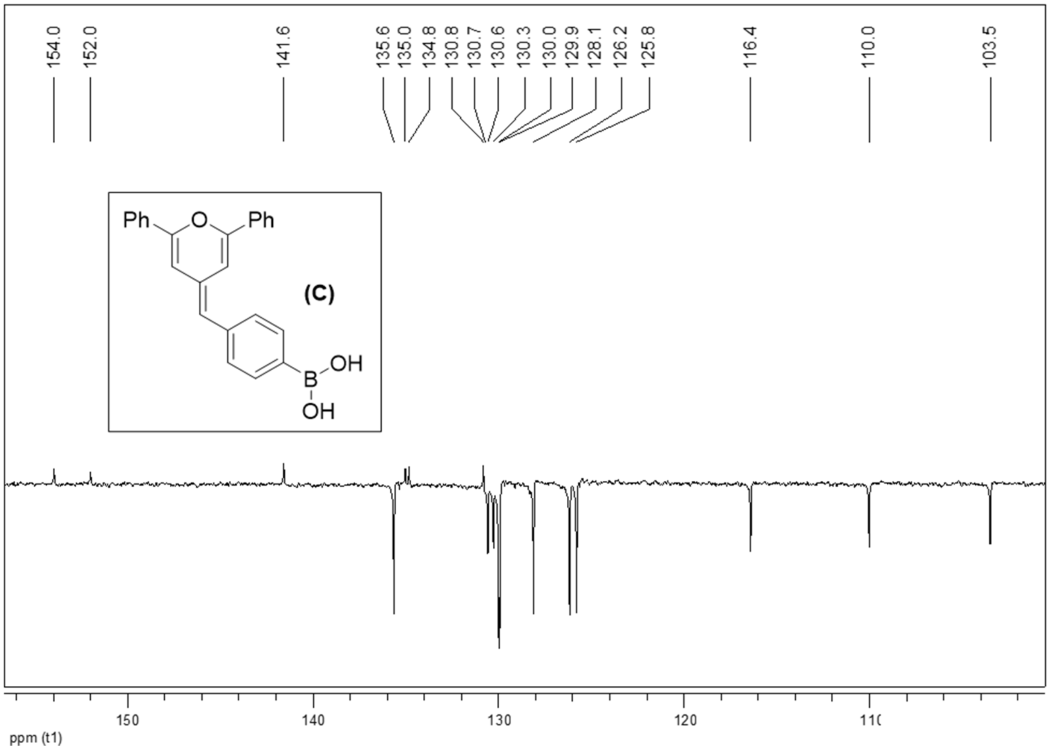

Figure S21. ^{13}**C NMR (APT) spectrum of compound C (100 MHz, THF-d**$_8$**).**

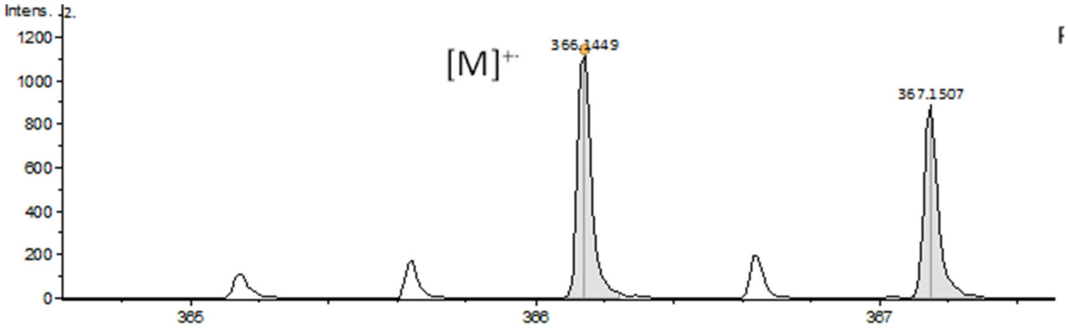

Figure S22. HRMS (ESI$^+$**) spectrum of compound C.**

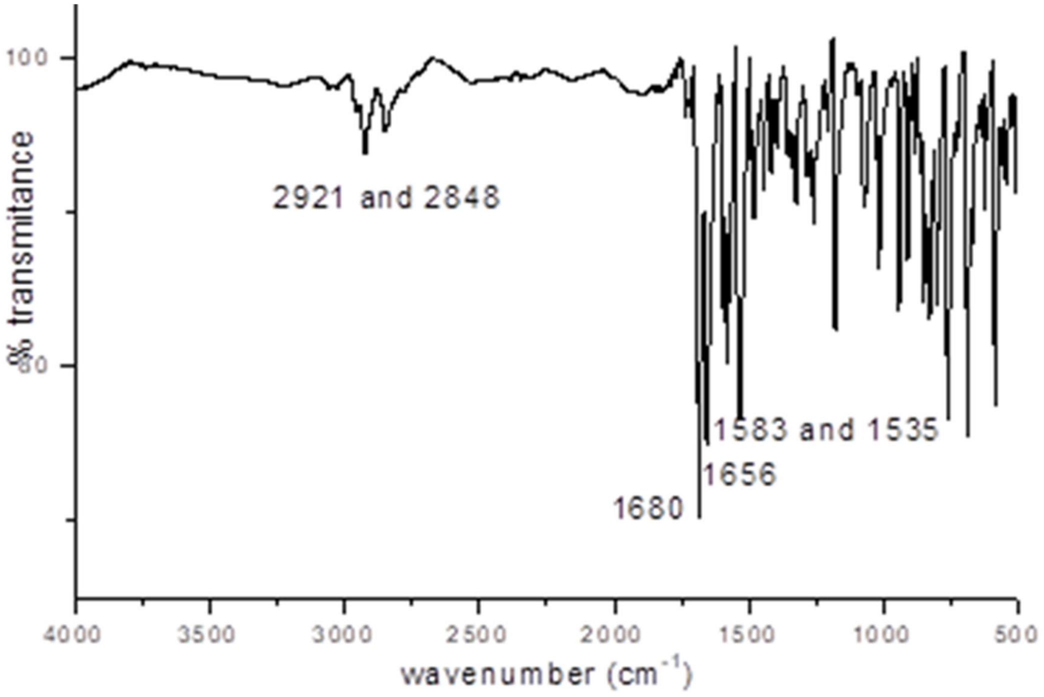

Figure S23. IR spectrum (KBr) of compound D.

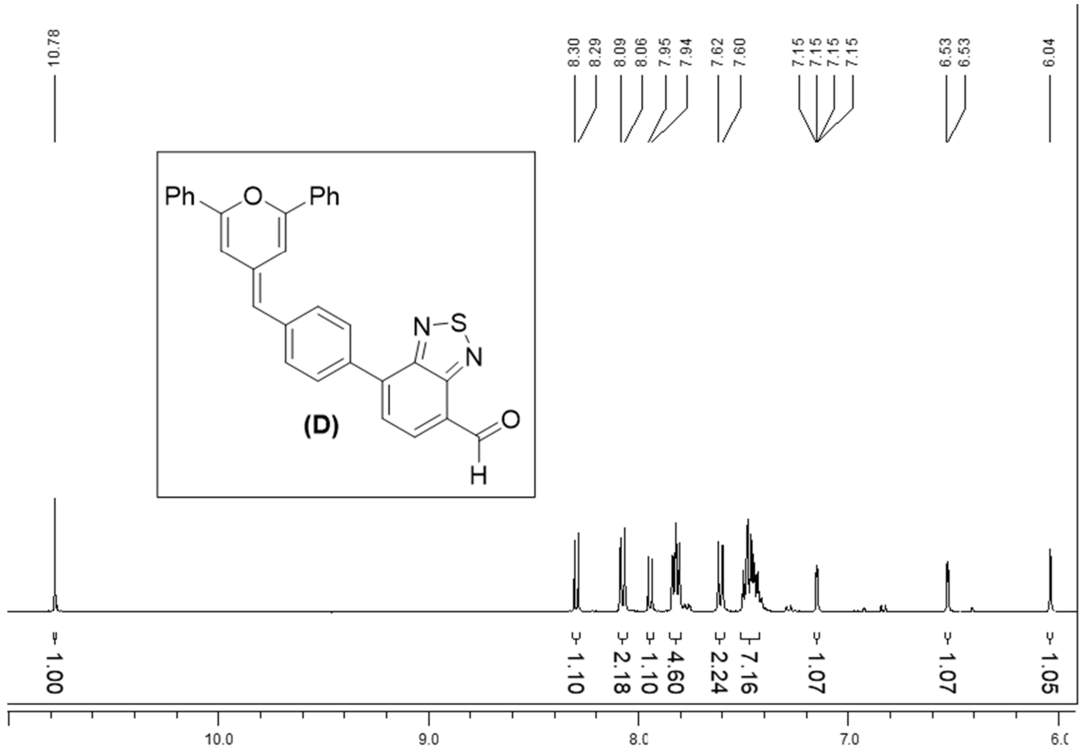

Figure S24. ^{1}H NMR spectrum of compound D (400 MHz, CH_2Cl_2).

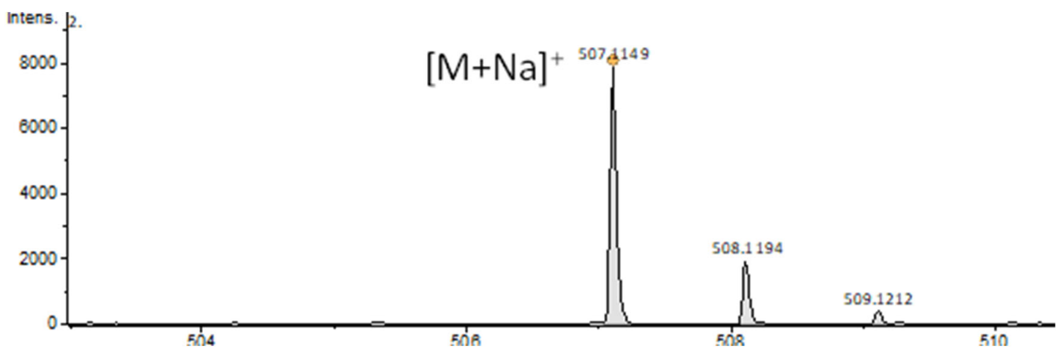

Figure S25. HRMS (ESI+) spectrum of compound D.

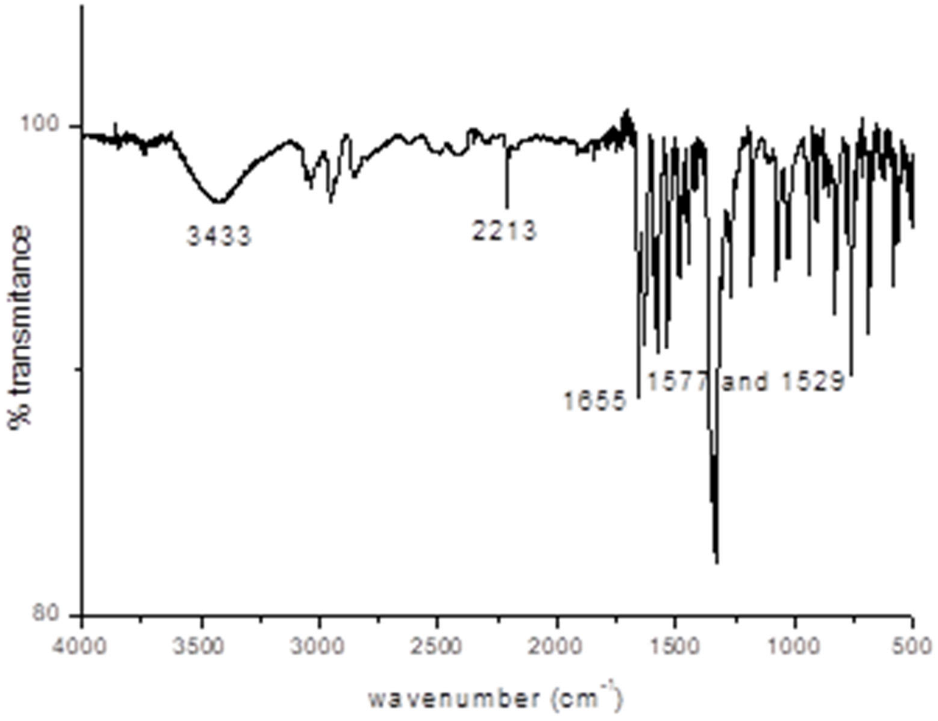

Figure S26. IR spectrum (KBr) of compound 14.

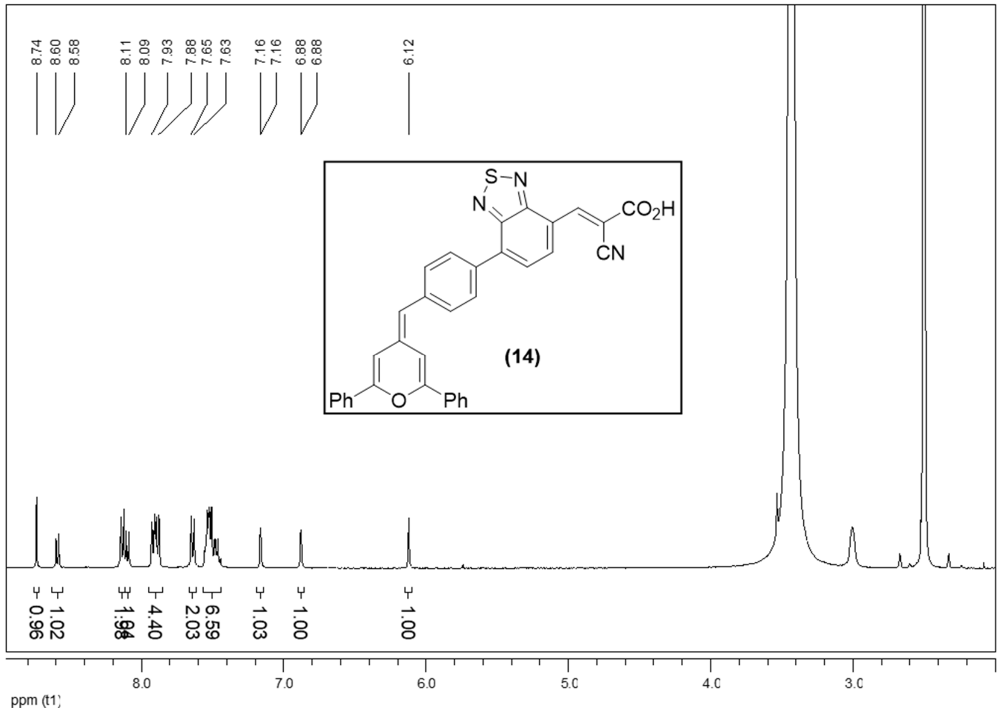

Figure S27. ^{1}H NMR spectrum of compound 14 (400 MHz, DMSO-d6).

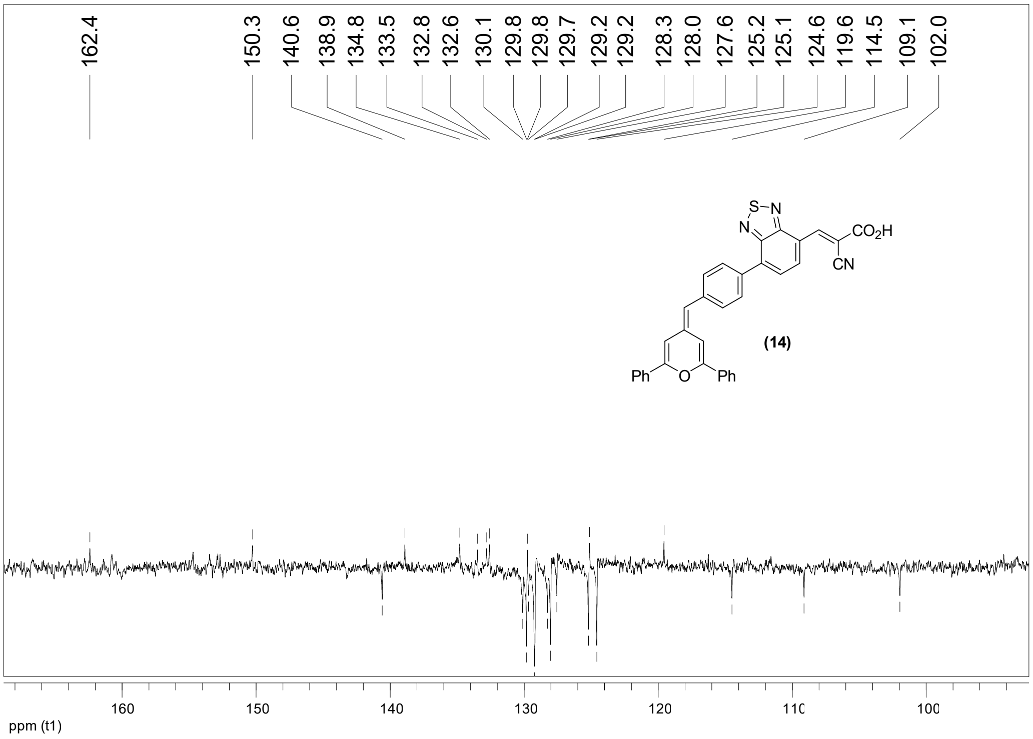

Figure S28. ^{13}C NMR (APT) spectrum of compound 14 (100 MHz, DMSO-d6).

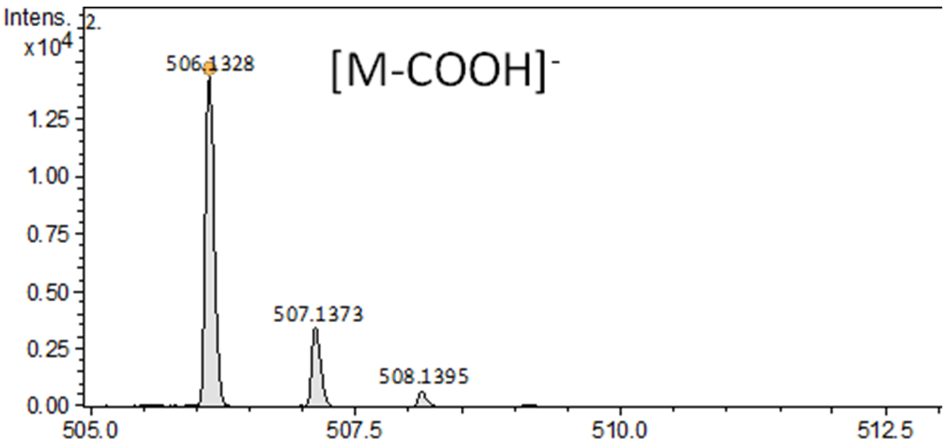

Figure S29. HRMS (ESI-) spectrum of compound 14.

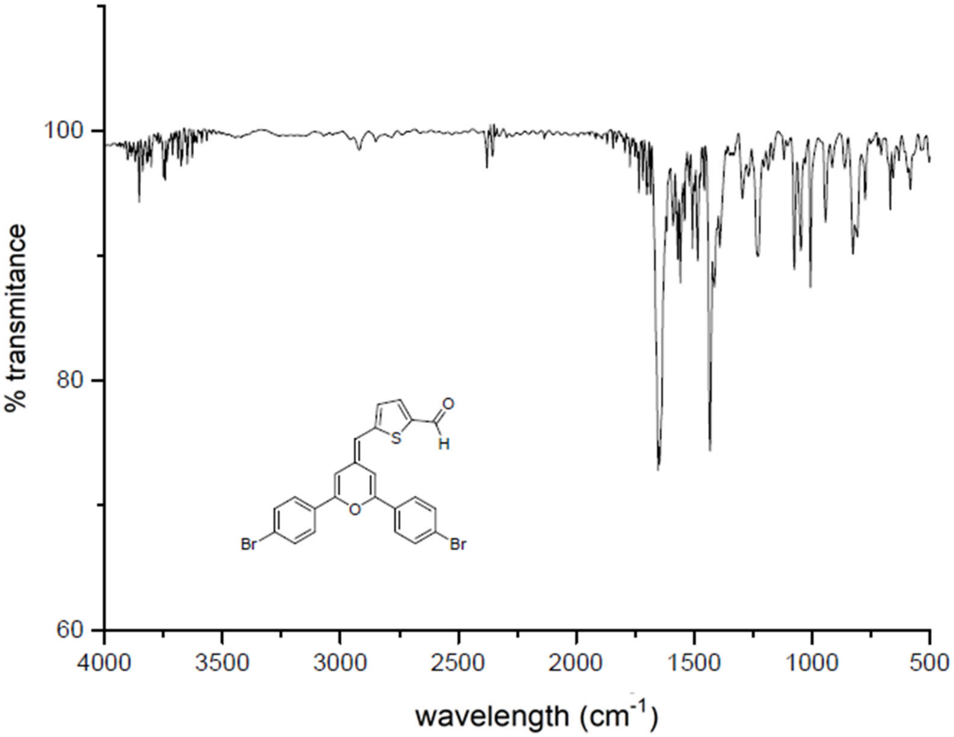

Figure S30. IR spectrum (KBr) of compound 20

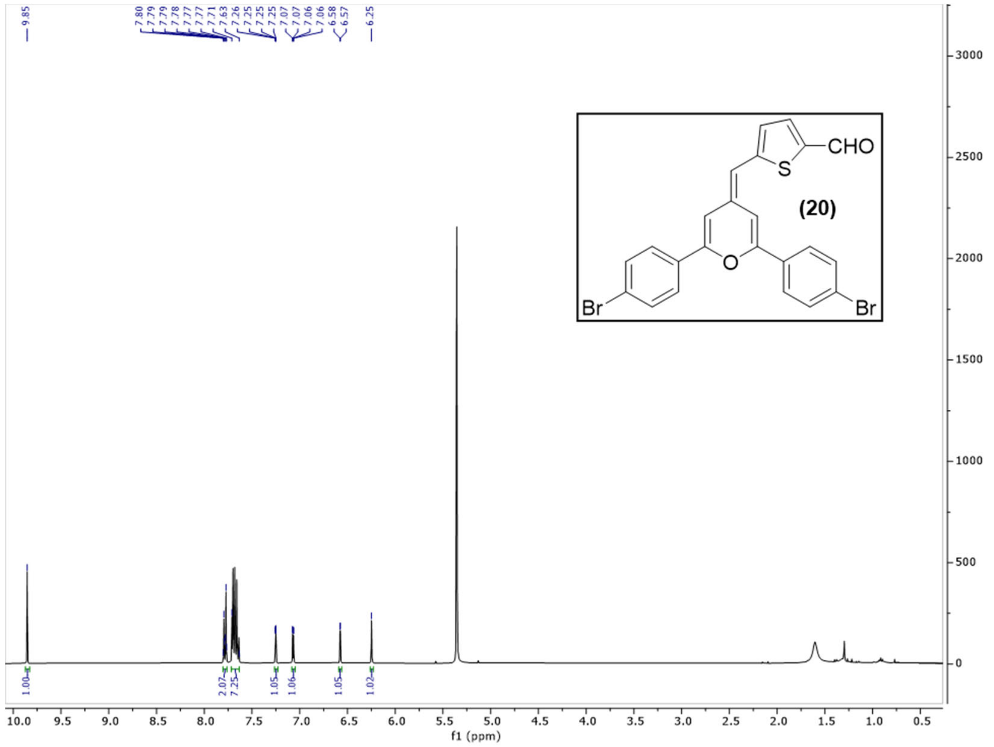

Figure S31. ^{1}H NMR spectrum of compound 20 (400 MHz, CD_2Cl_2).

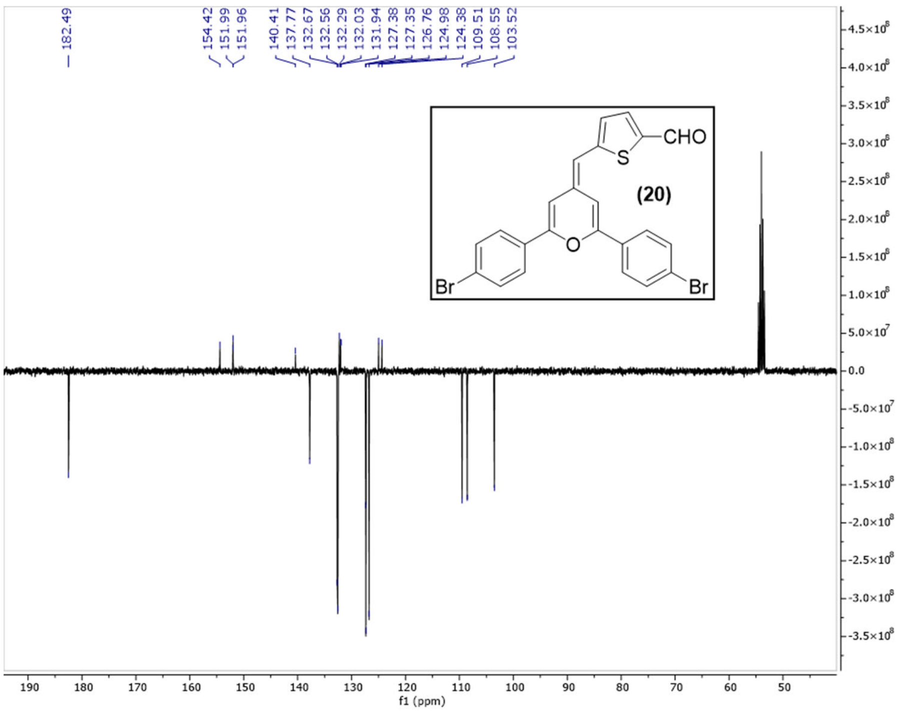

Figure S32. ^{13}C NMR (APT) spectrum of compound 20 (100 MHz, $CDCl_3$).

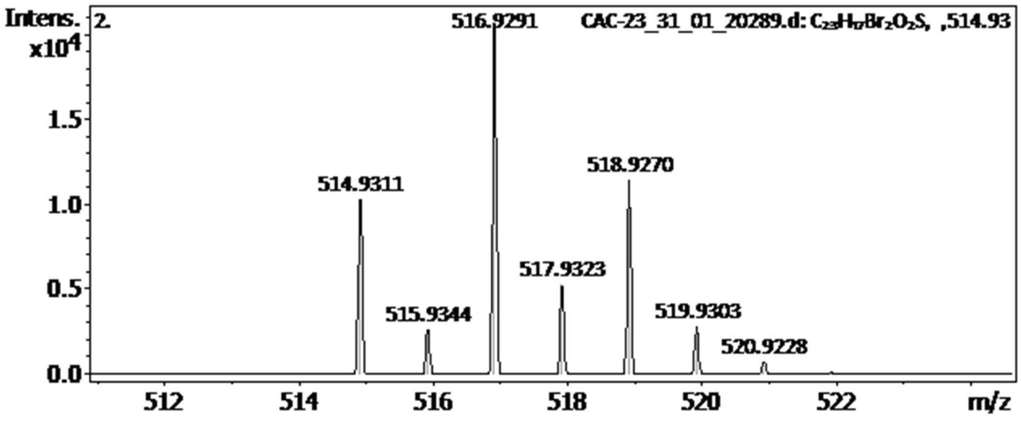

Fig. S33. HRMS (ESI+) spectrum of compound 20

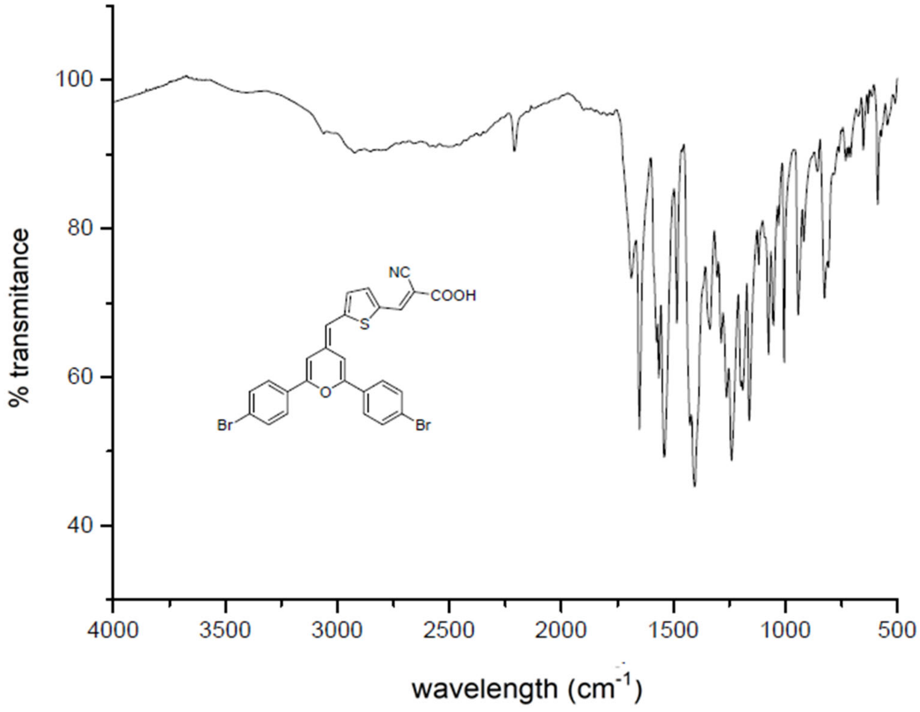

Figure S34. IR spectrum (KBr) of compound 21

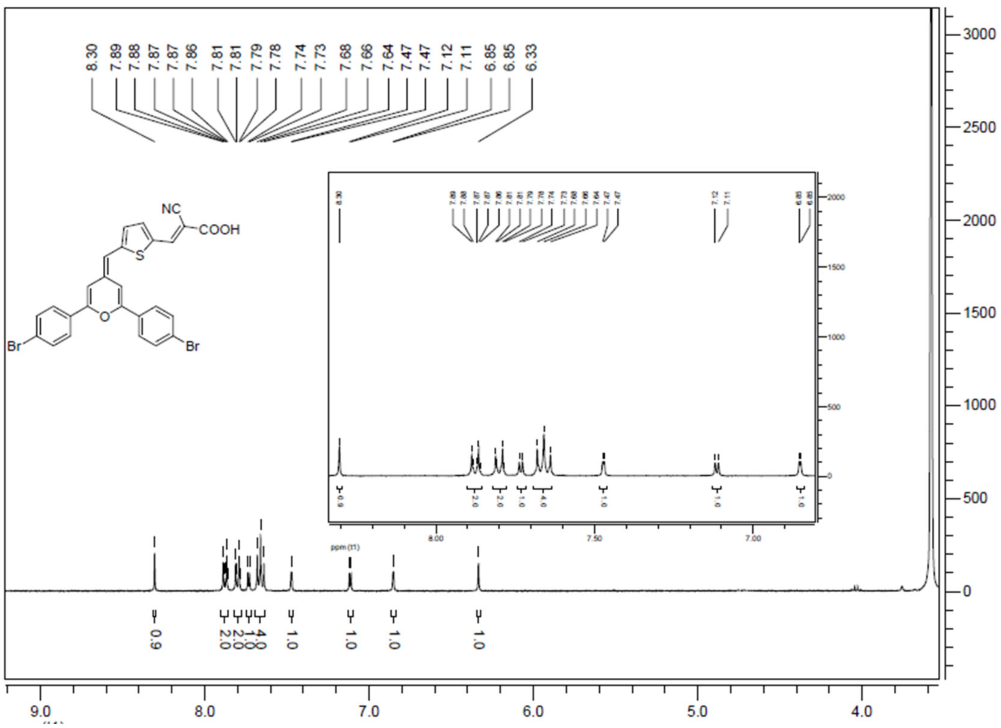

Figure S35. ¹H NMR spectrum of compound 21 (400 MHz, THF-d₈).

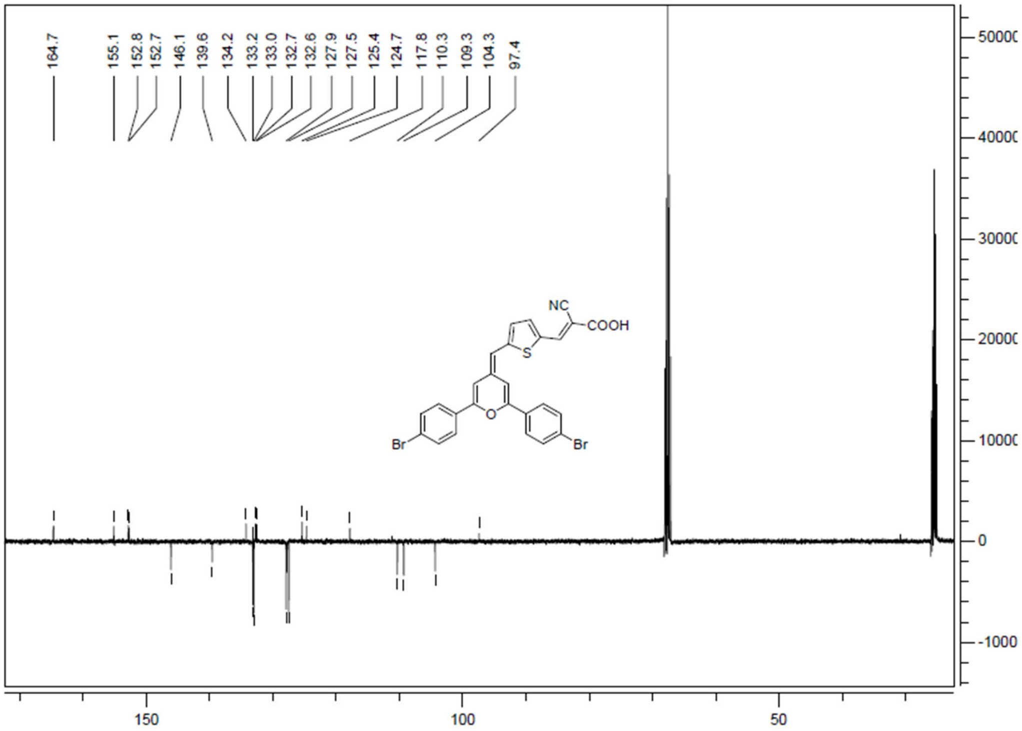

Figure S36. ¹³C NMR (APT) spectrum of compound 21 (100 MHz, THF-d₈).

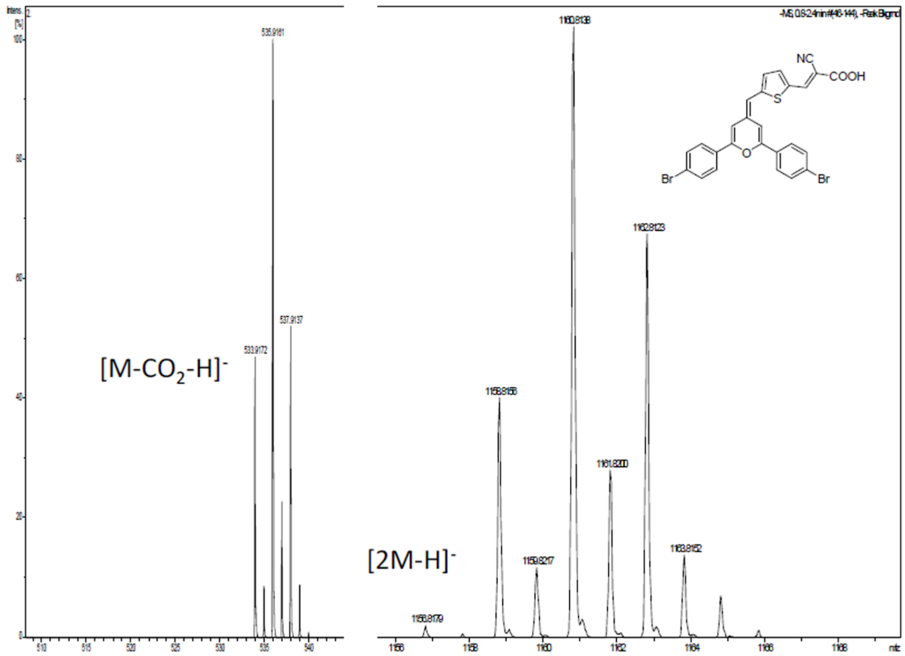

Fig. S37. HRMS (ESI+) spectrum of compound 21

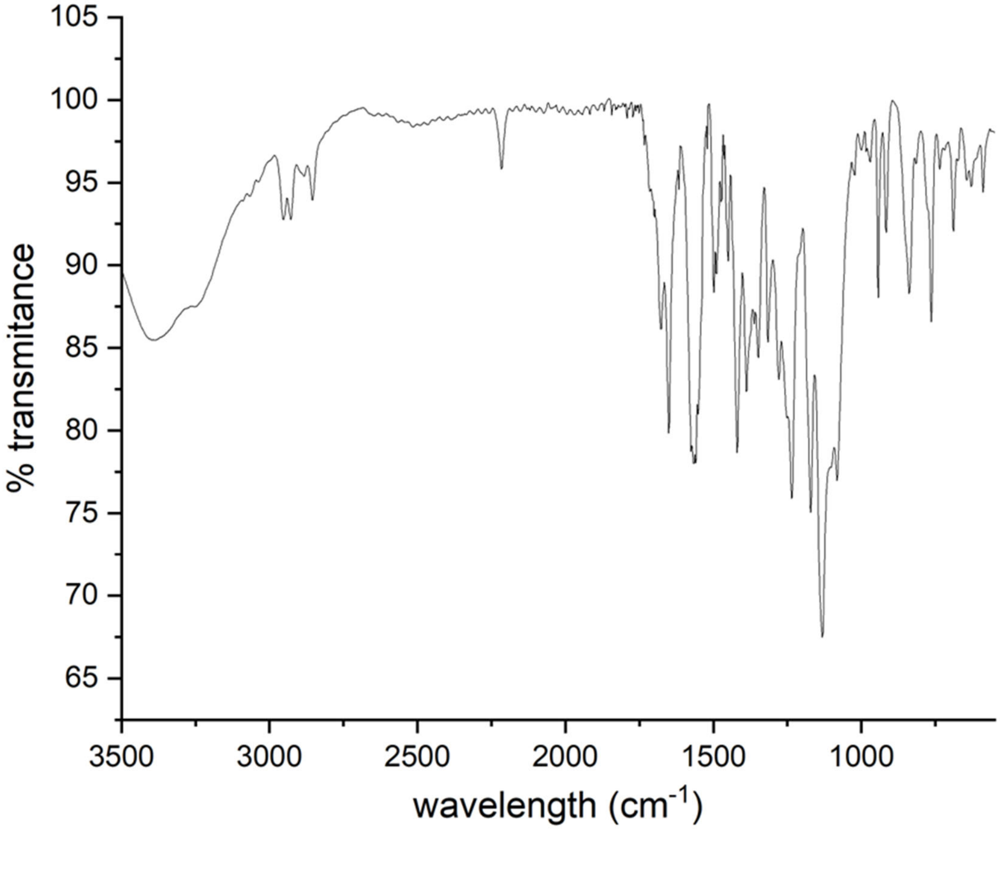

Figure S38. IR spectrum (KBr) of compound 26

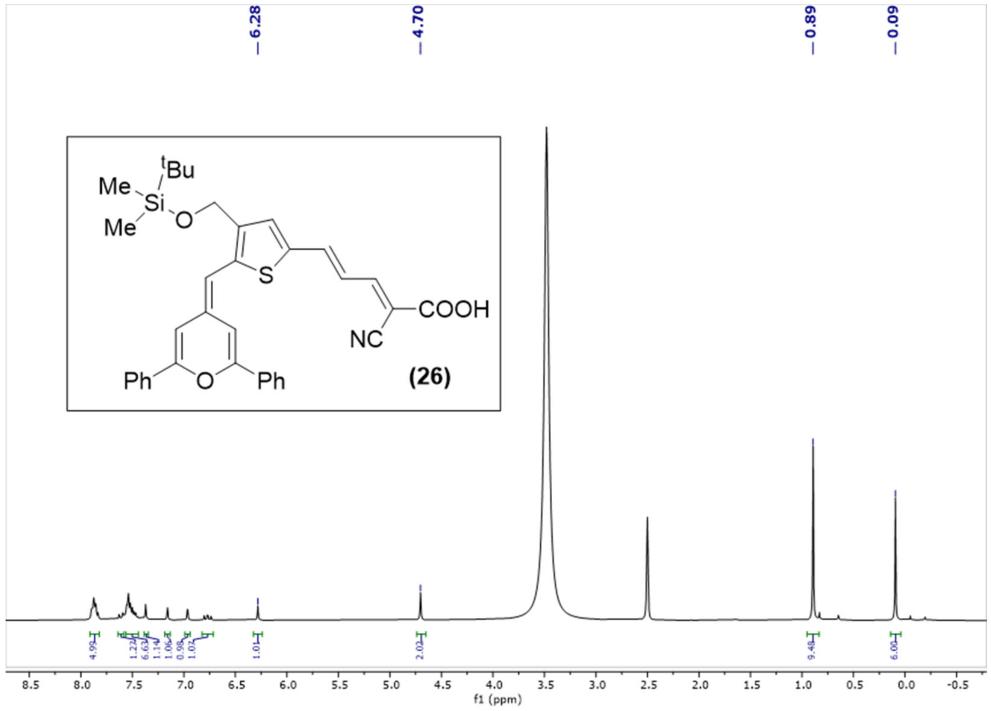

Figure S39. ^{1}H NMR spectrum of compound 26 (400 MHz, DMSO-d_6).

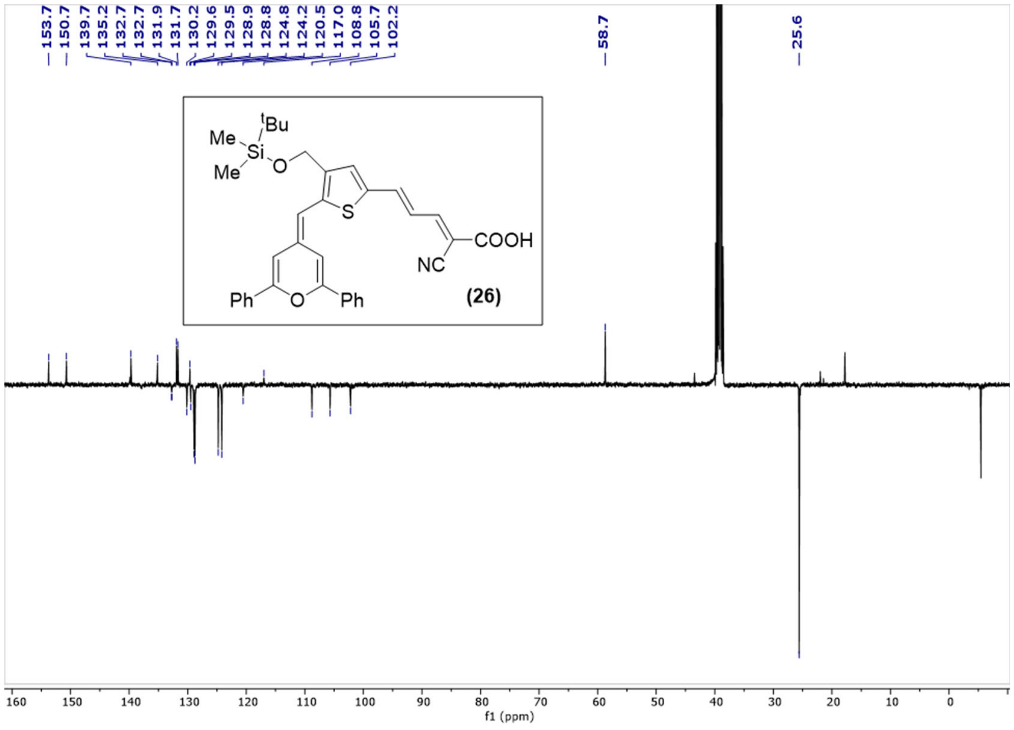

Figure S40. ^{13}C NMR (APT) spectrum of compound 26 (100 MHz, DMSO-d_6).

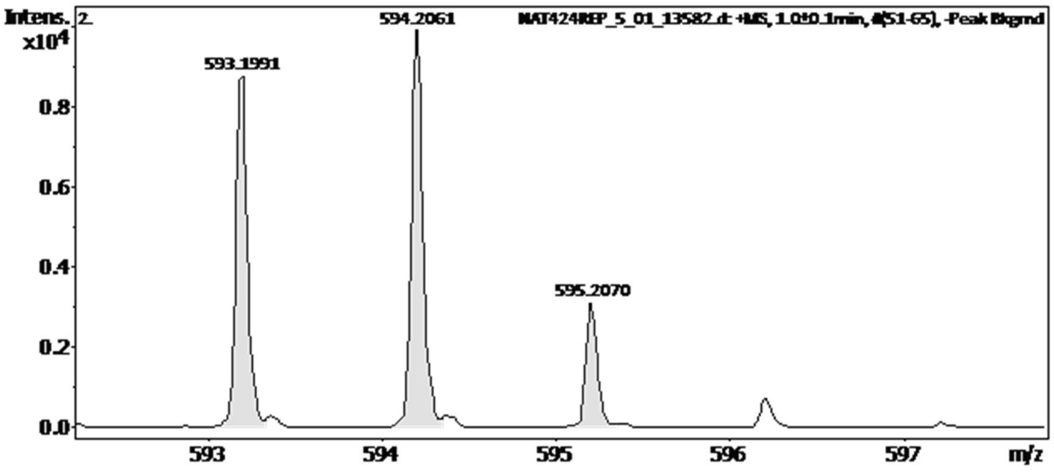

Fig. S41. HRMS (ESI+) spectrum of compound 26

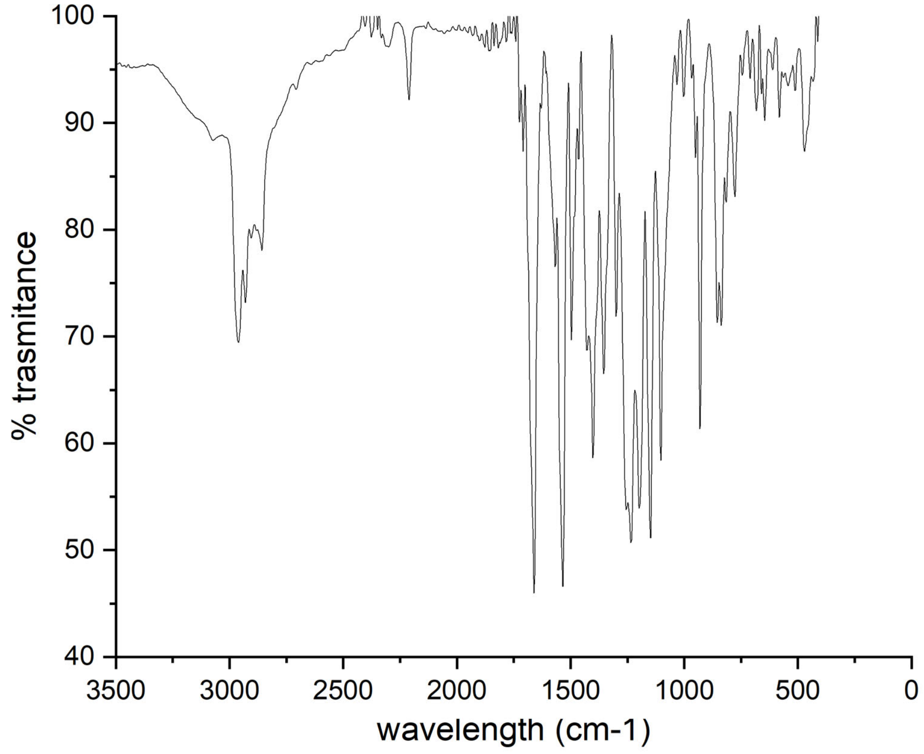

Figure S42. IR spectrum (KBr) of compound 27

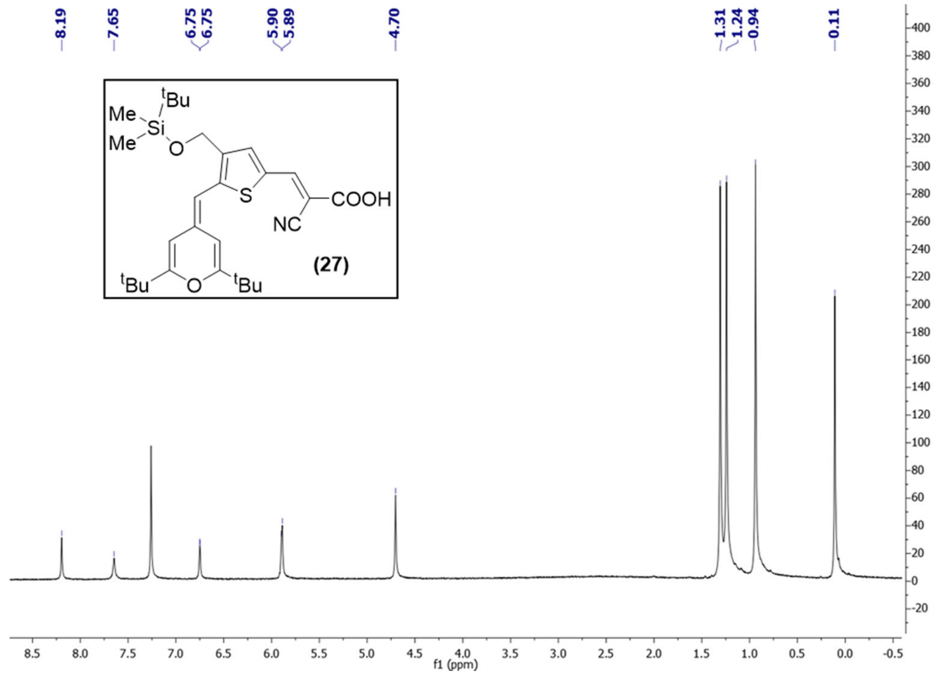

Figure S43. **^{1}H NMR spectrum of compound 27 (400 MHz, $CDCl_3$).**

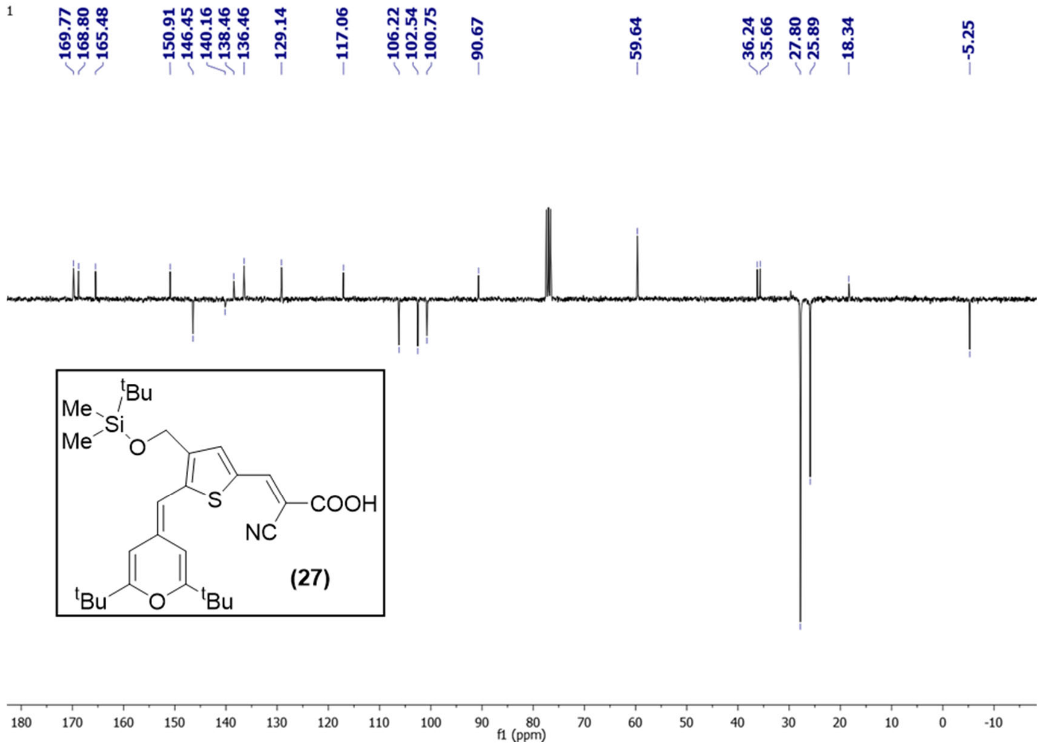

Figure S44. **^{13}C NMR (APT) spectrum of compound 27 (100 MHz, $CDCl_3$).**

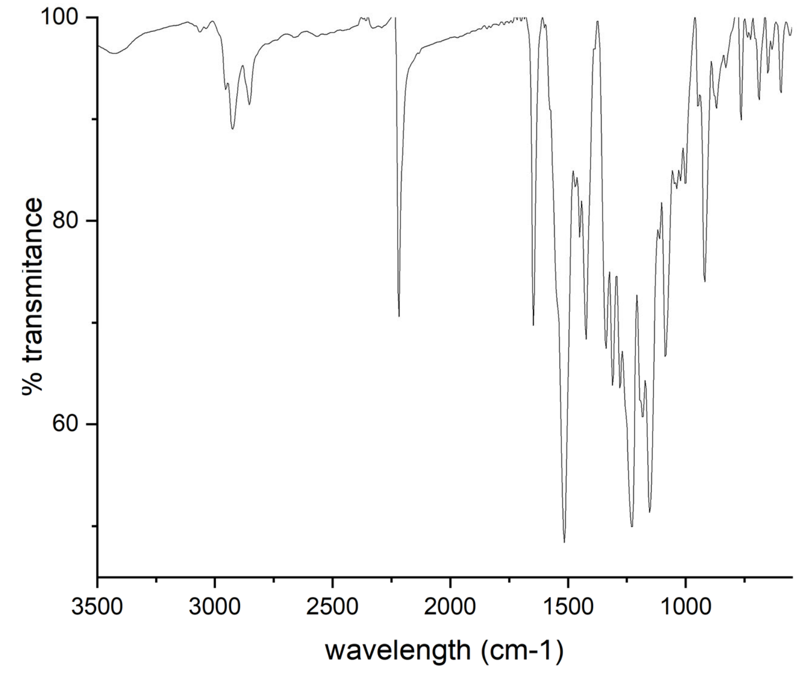

Figure S45. IR spectrum (KBr) of compound 30

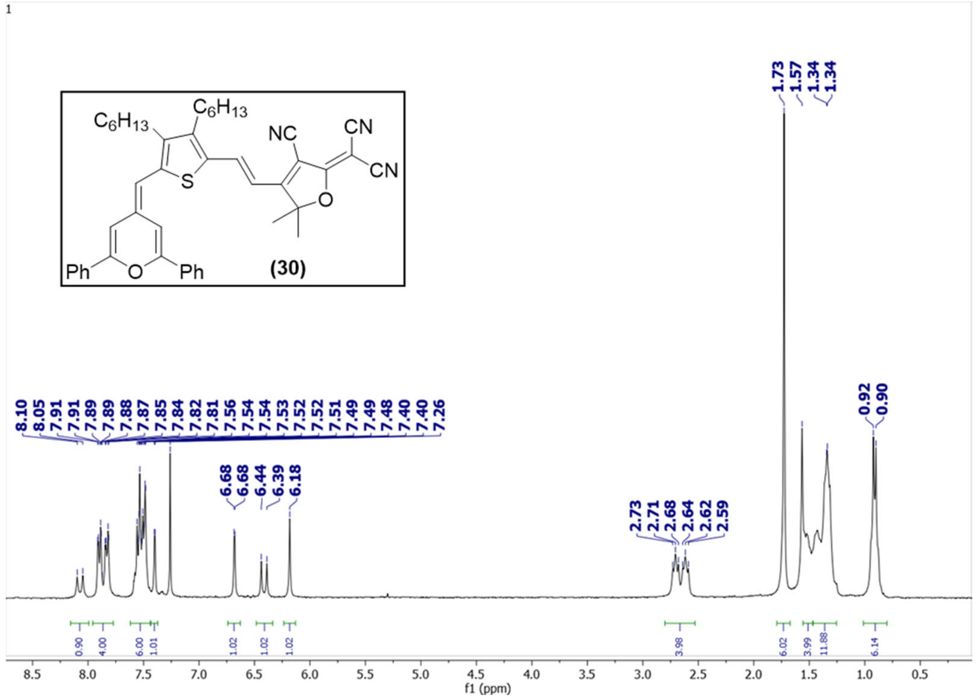

Figure S46. ¹H NMR spectrum of compound 30 (400 MHz, CDCl₃).

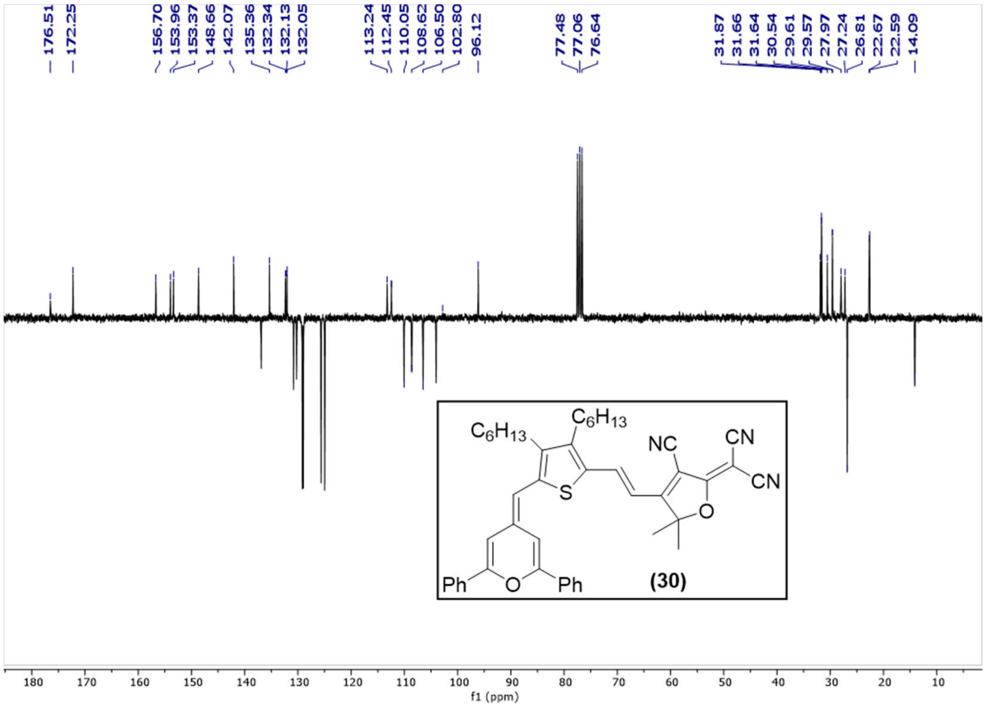

Figure S47. **^{13}C NMR (APT) spectrum of compound 30 (100 MHz, $CDCl_3$).**

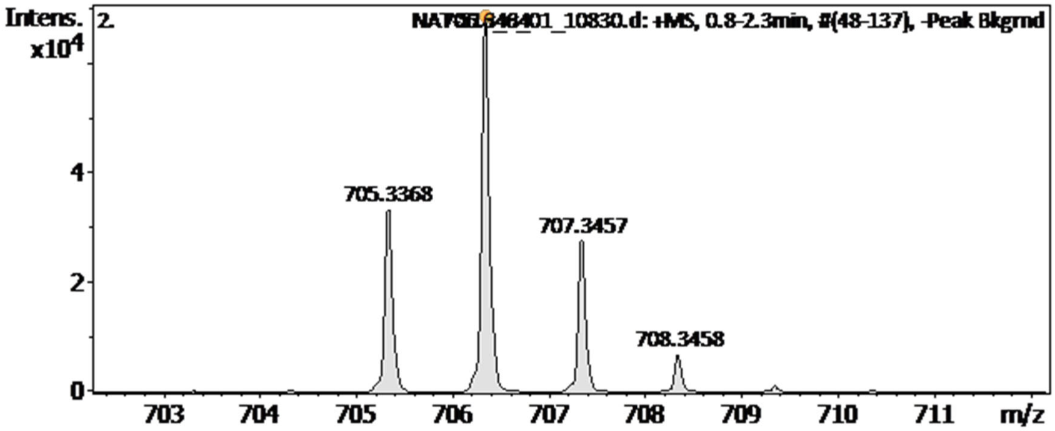

Fig. S48. HRMS (ESI^+) spectrum of compound 30

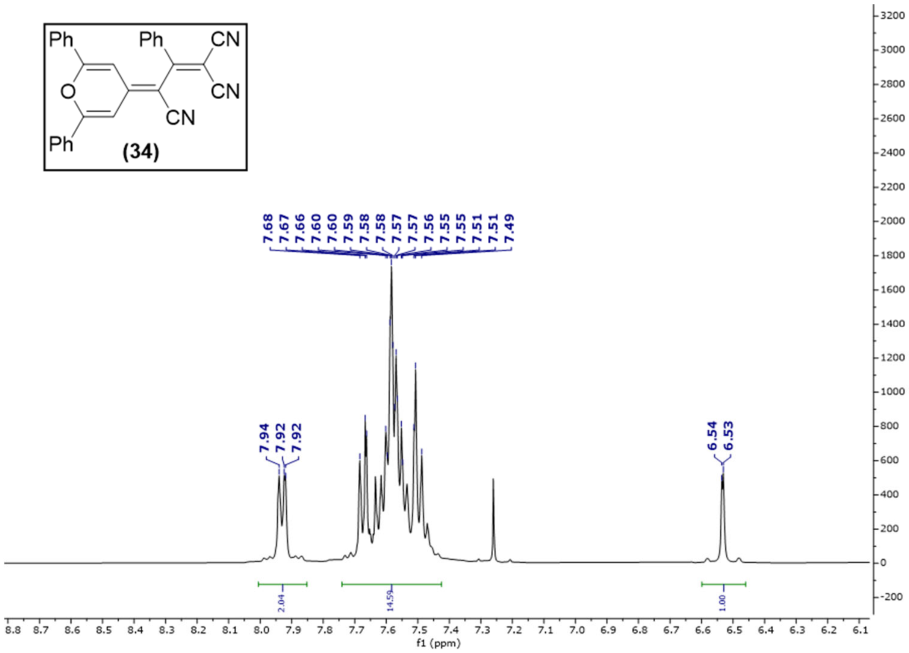

Figure S49. ^{1}H NMR spectrum of compound 34 (400 MHz, $CDCl_3$).

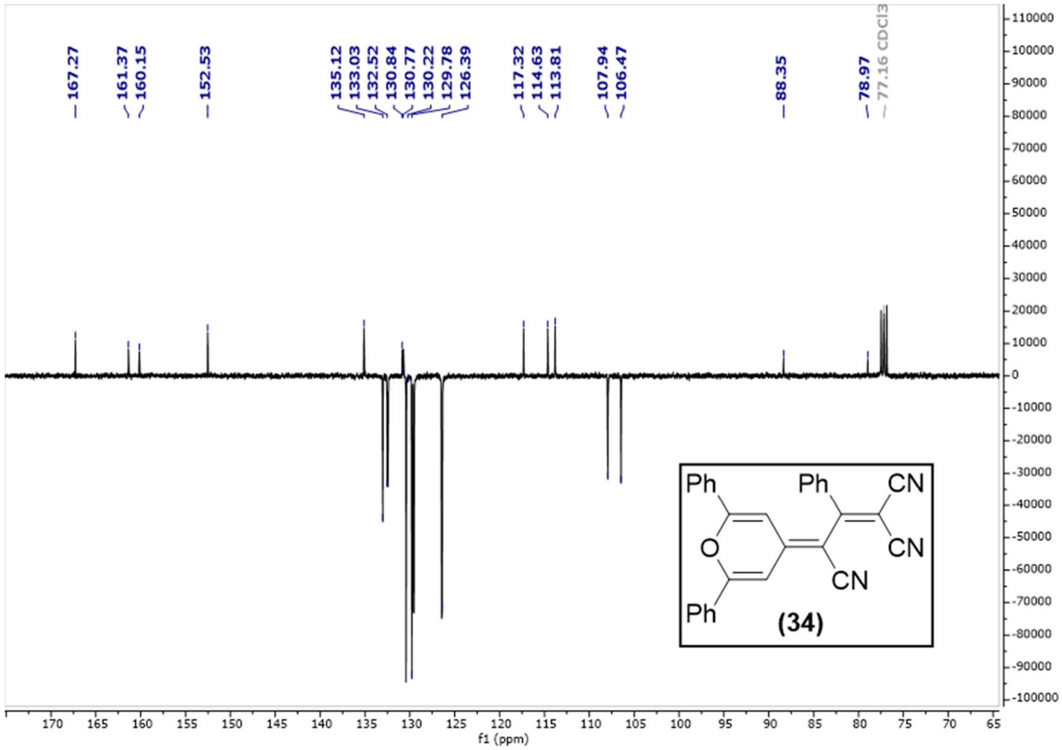

Figure S50. ^{13}C NMR (APT) spectrum of compound 34 (100 MHz, $CDCl_3$).

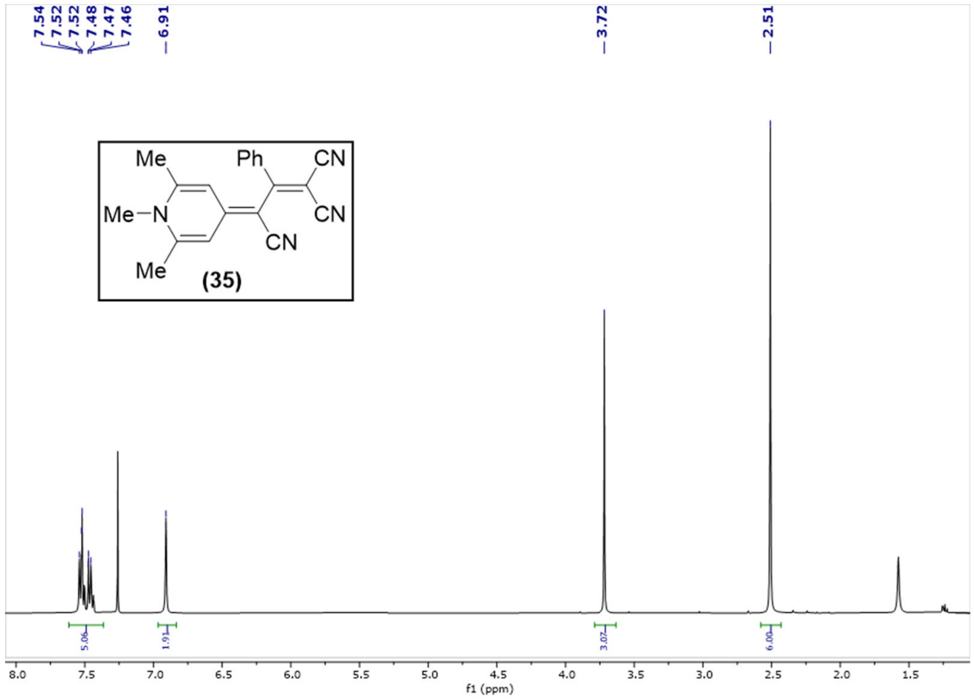

Figure S51. **^{1}H NMR spectrum of compound 35 (400 MHz, $CDCl_3$).**

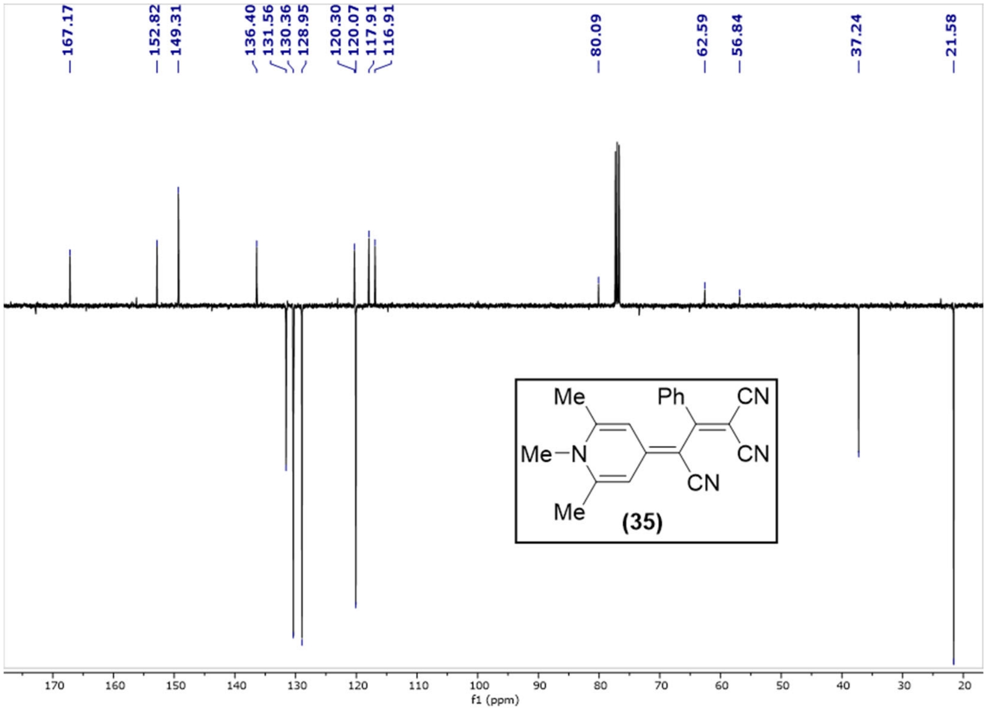

Figure S52. **^{13}C NMR (APT) spectrum of compound 35 (100 MHz, $CDCl_3$)**

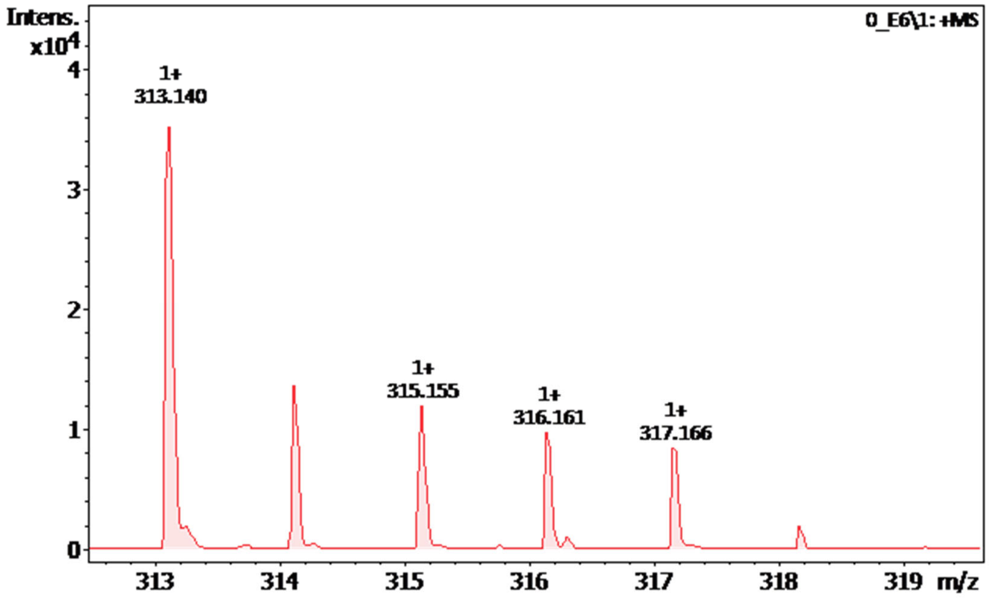

Fig. S53. MS (MALDI⁺) spectrum of compound 35

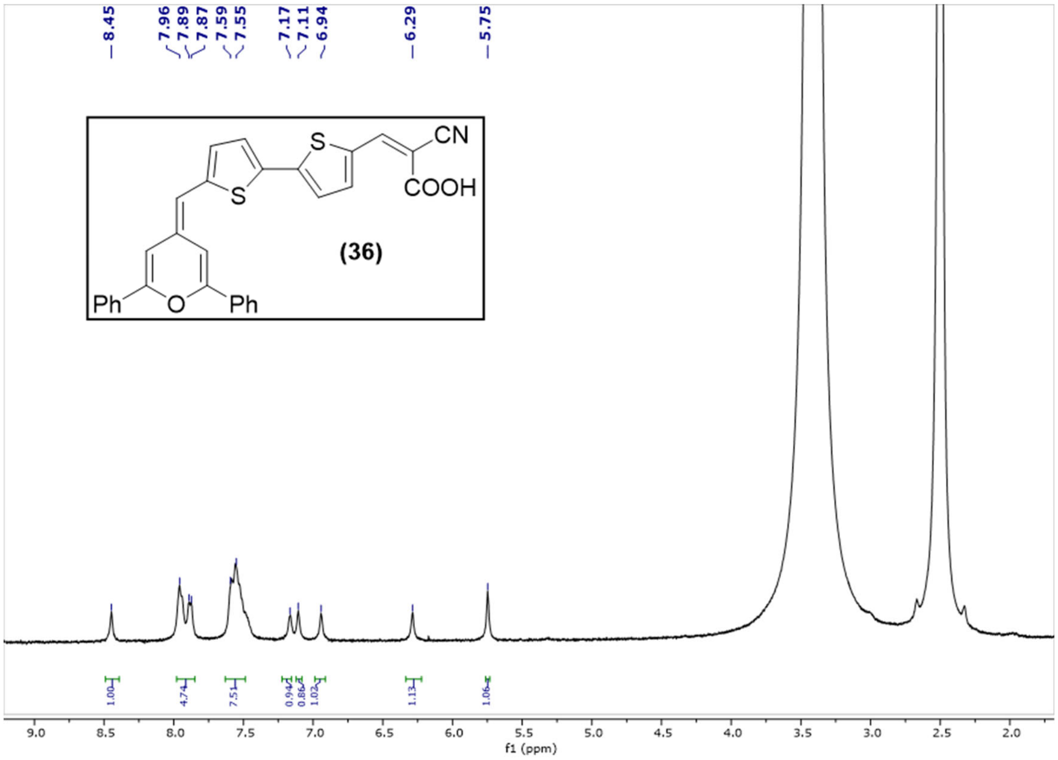

Figure S54. ¹H NMR spectrum of compound 36 (400 MHz, $CDCl_3$).

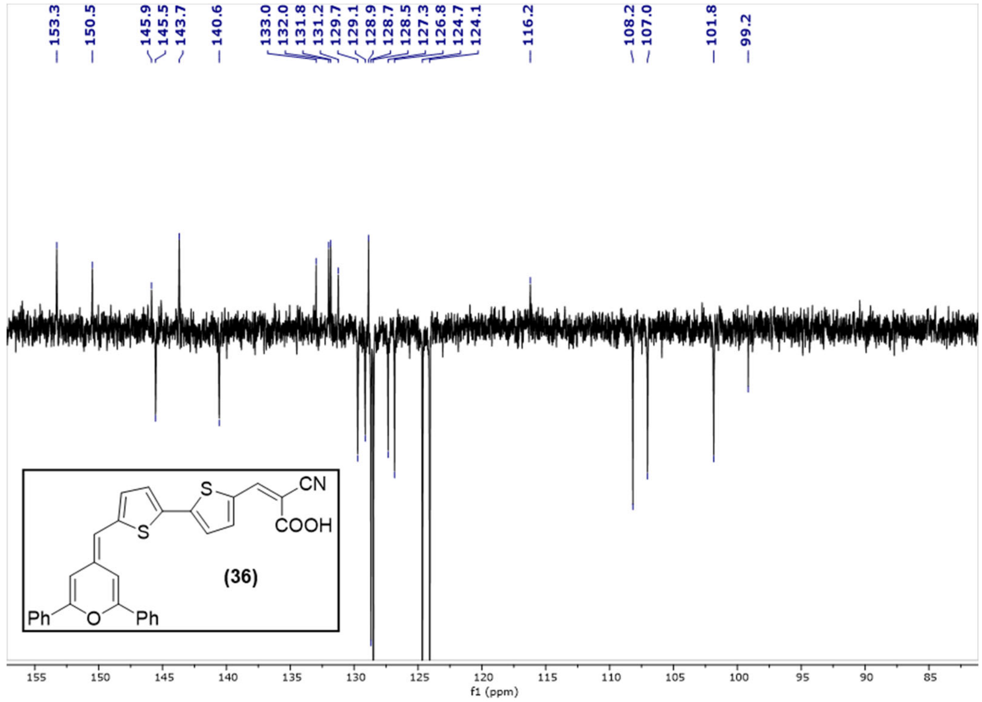

Figure S55. ^{13}C **NMR (APT) spectrum of compound 36 (100 MHz,** $CDCl_3$**).**

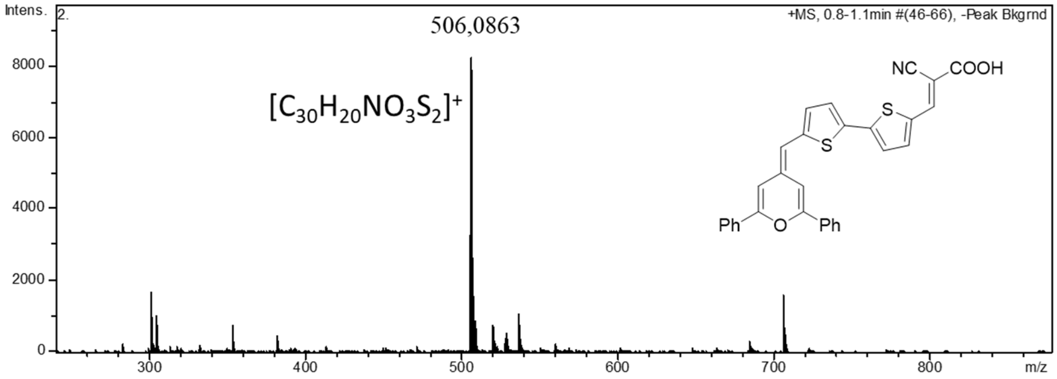

Fig. S56. HRMS (ESI+) spectrum of compound 36

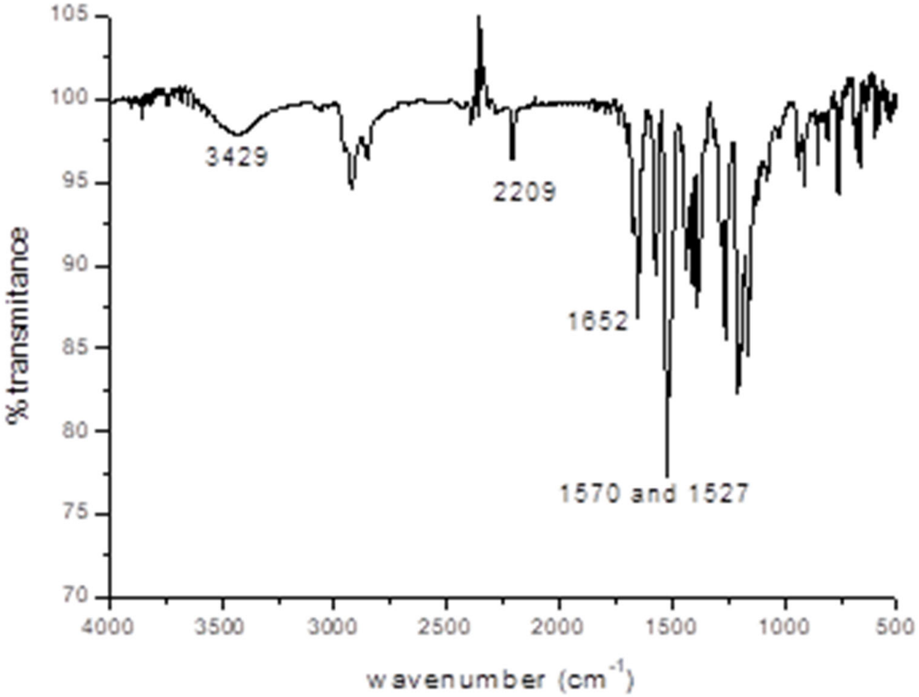

Figure S57. IR spectrum (KBr) of compound 37

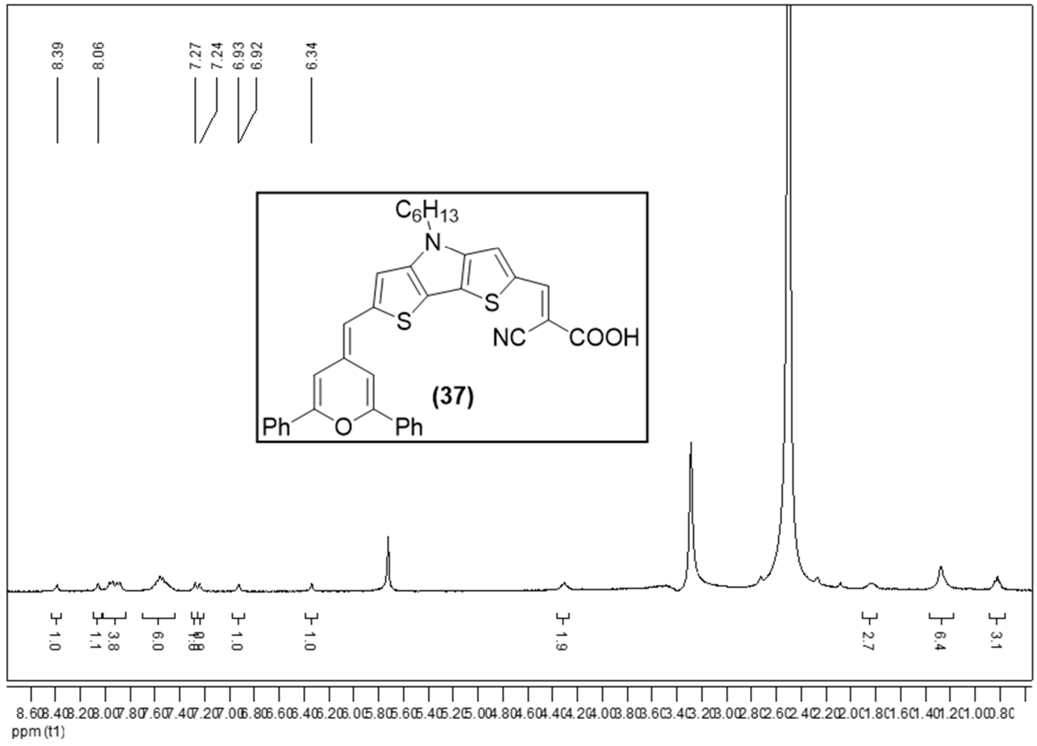

Figure S58. ^{1}H NMR spectrum of compound 37 (300 MHz, 40 °C, DMSO-d_6).

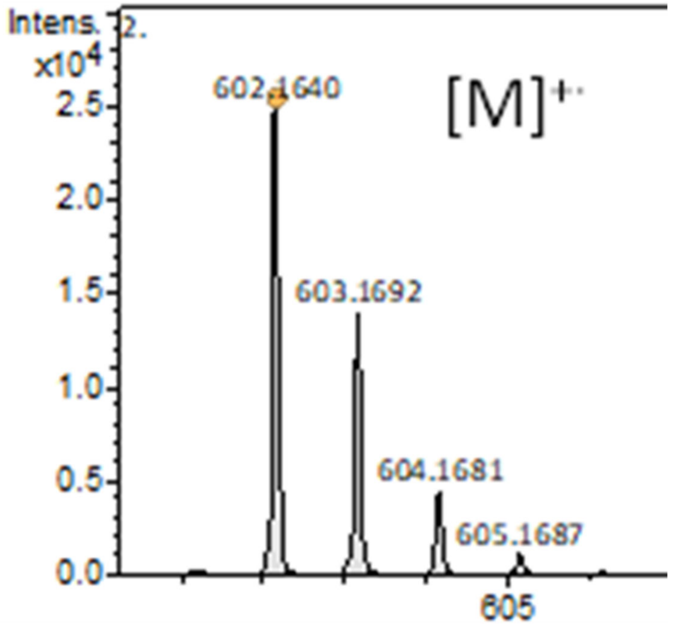

Fig. S59. HRMS (ESI+) spectrum of compound 37

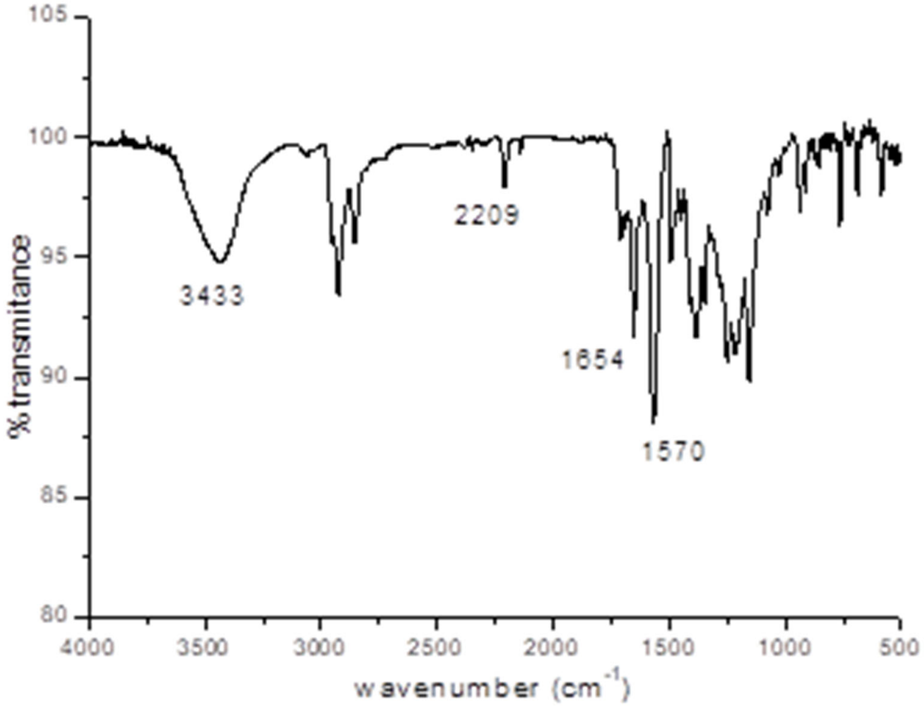

Figure S60. IR spectrum (KBr) of compound 38

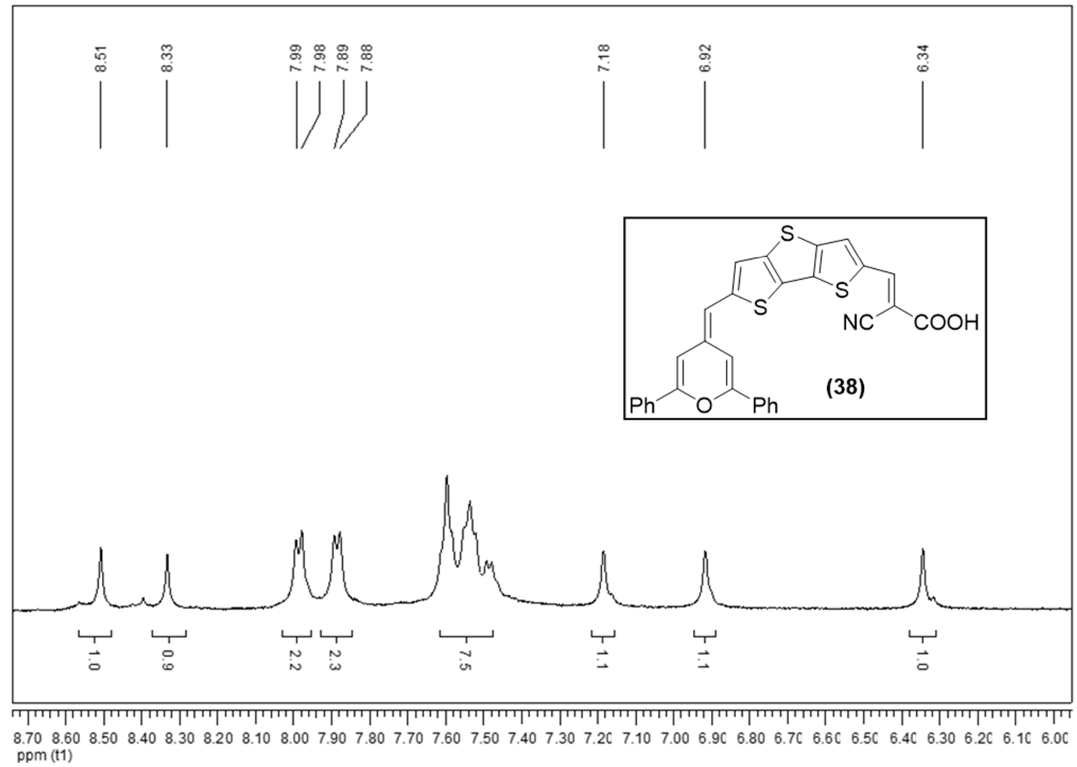

Figure S61. ^{1}H NMR spectrum of compound 38 (500 MHz, 77 °C, DMSO-d$_6$).

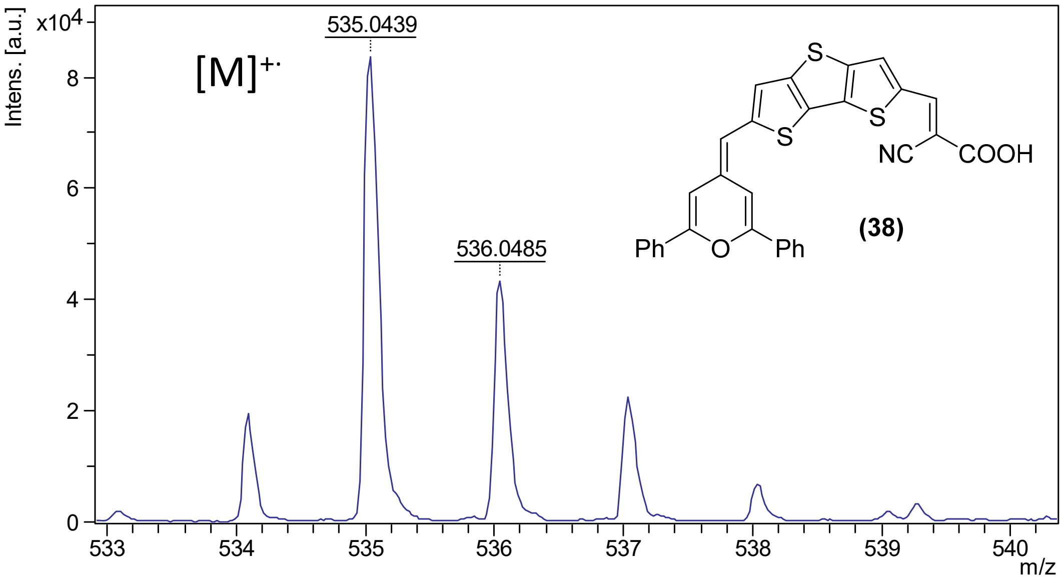

Fig. S62. HRMS (MALDI$^+$) spectrum of compound 38

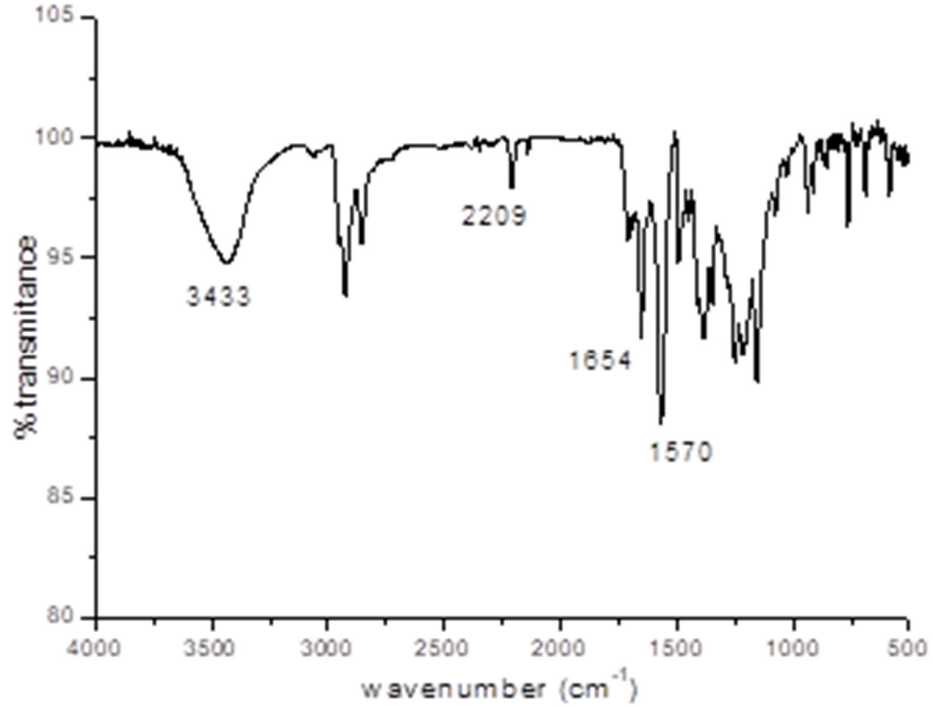

Figure S63. IR spectrum (KBr) of compound 39

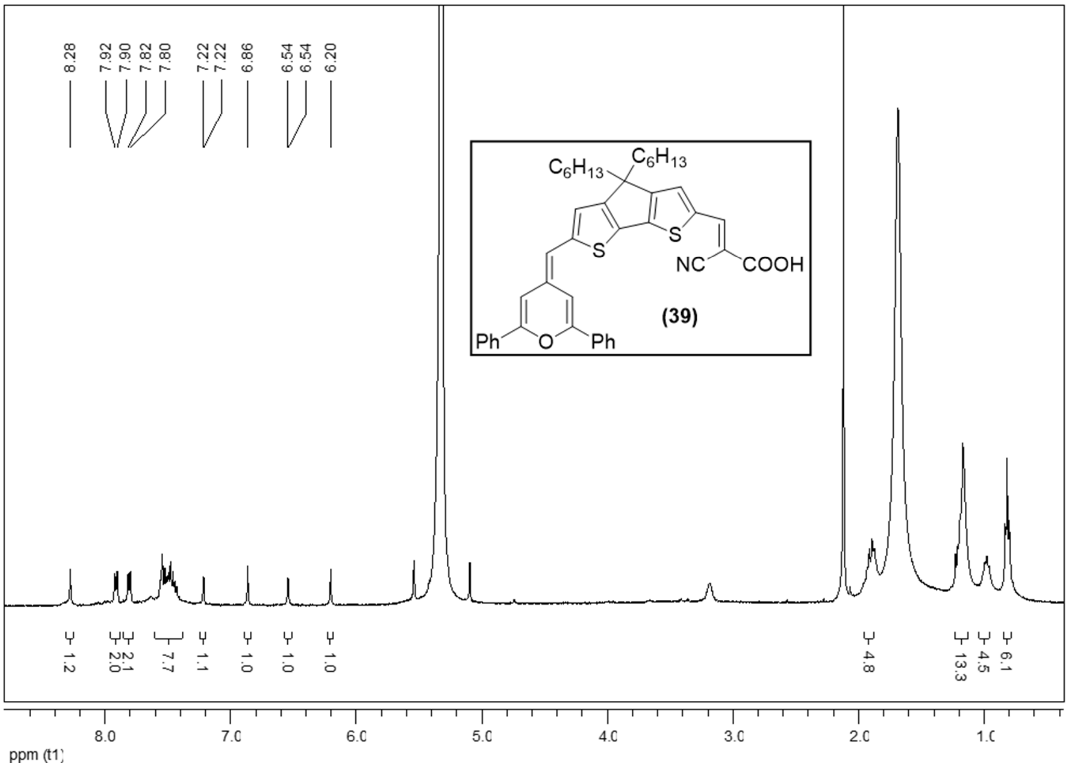

Figure S64. ^{1}H NMR spectrum of compound 39 (400 MHz, CD_2Cl_2).

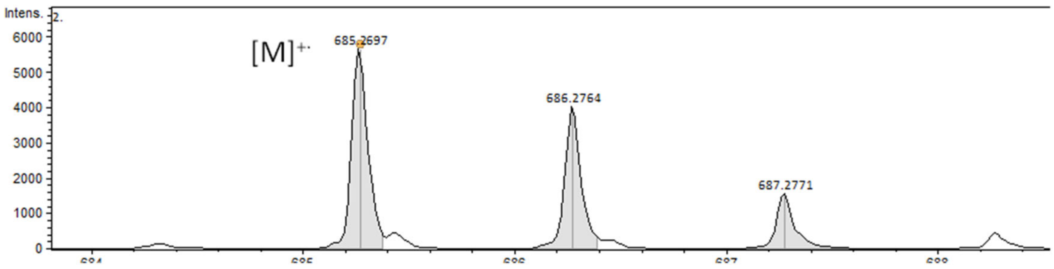

Fig. S65. HRMS (ESI+) spectrum of compound 39

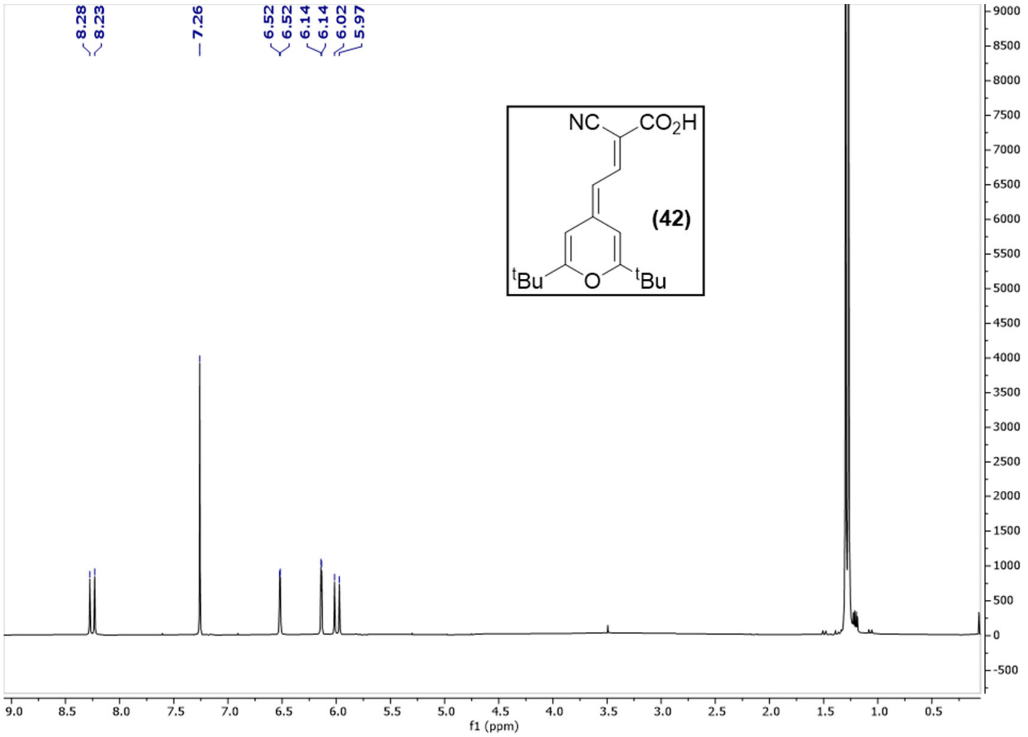

Figure S66. ^{1}H NMR spectrum of compound 42 (400 MHz, $CDCl_3$).

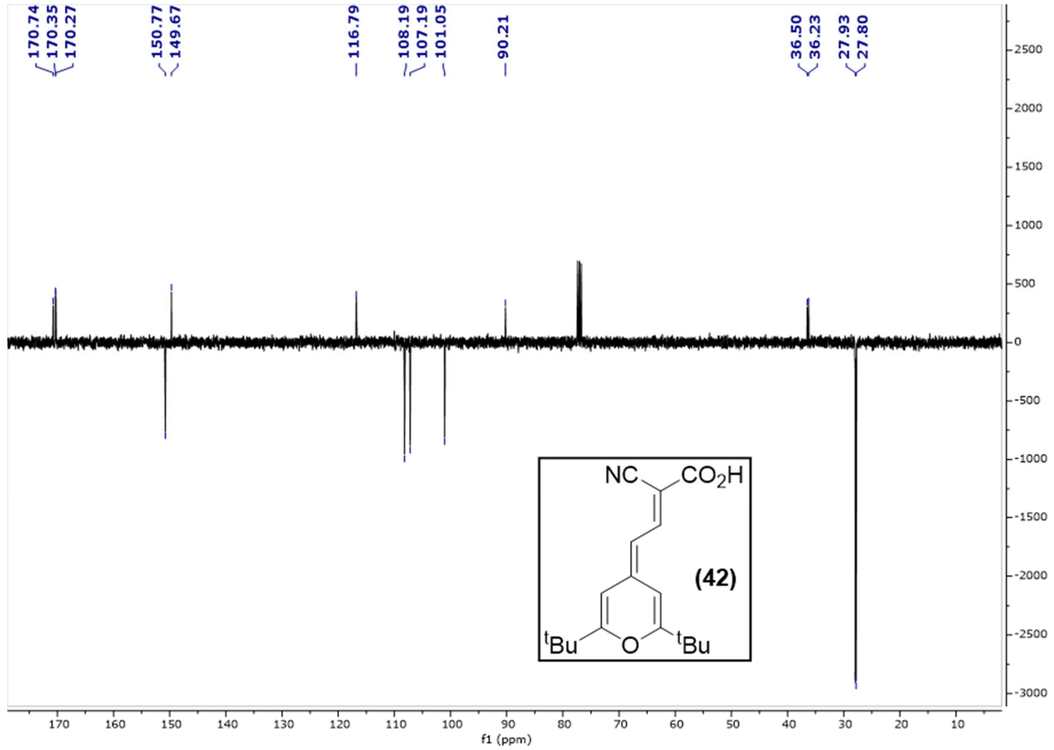

Figure S67. ^{13}**C NMR (APT) spectrum of compound 42 (100 MHz, CDCl$_3$).**

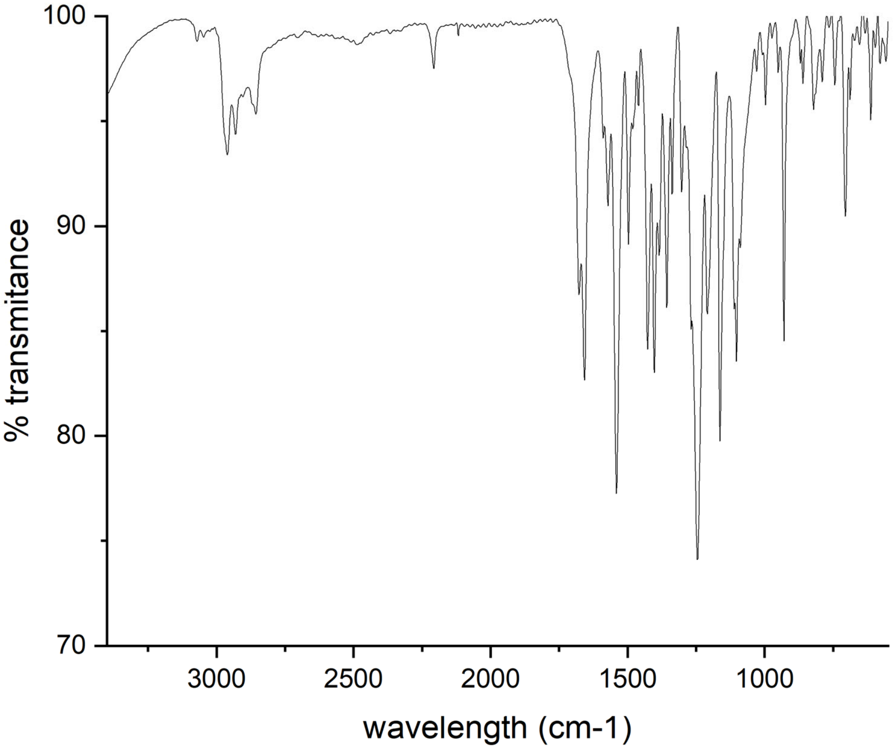

Figure S68. IR spectrum (KBr) of compound 46

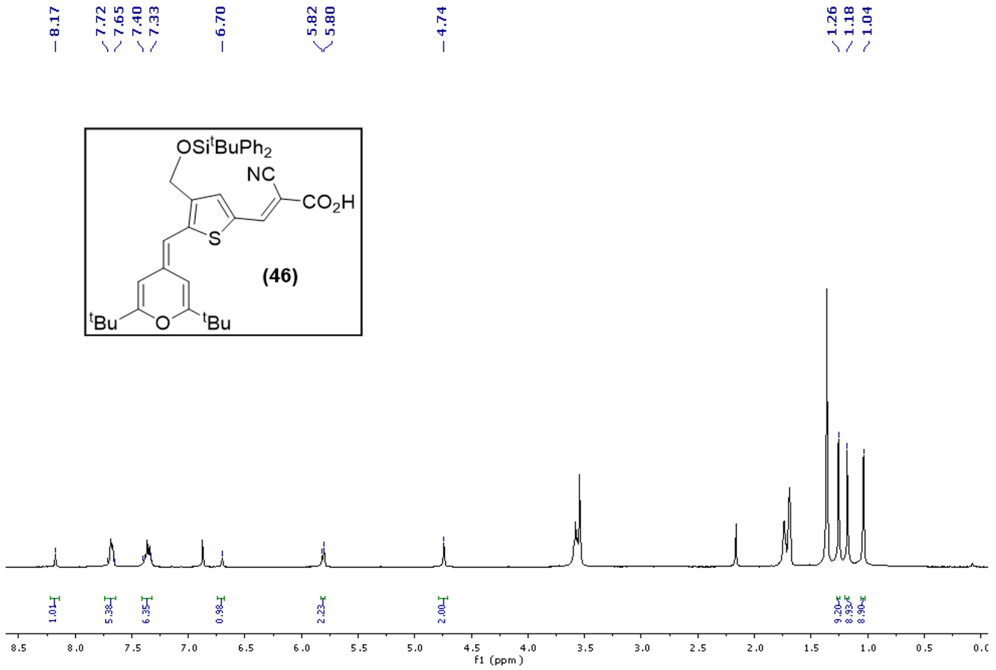

Figure S69. ^{1}H **NMR spectrum of compound 46 (400 MHz, THF-d**$_8$**).**

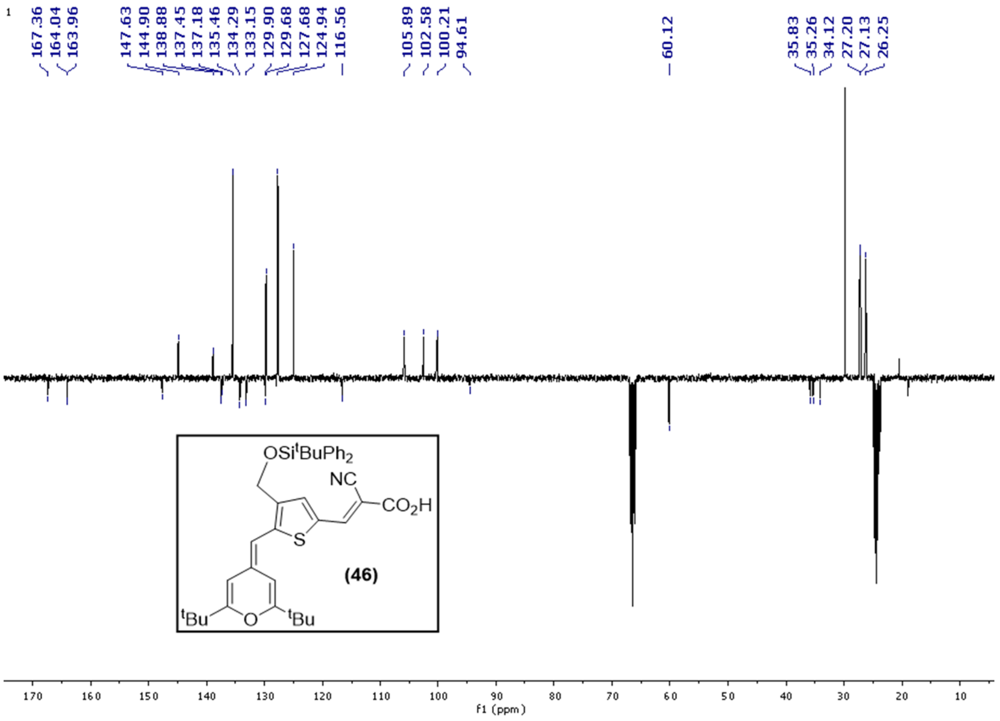

Figure S70. ^{13}C **NMR (APT) spectrum of compound 46 (100 MHz, THF-d**$_8$**).**

References

(1) Franco, S.; Garín, J.; Martínez de Baroja, N.; Pérez-Tejada, R.; Orduna, J.; Yu, Y.; Lira-Cantú, M. New D−π−A-Conjugated Organic Sensitizers Based on 4H-Pyran-4-Ylidene Donors for Highly Efficient Dye-Sensitized Solar Cells. *Org. Lett.* **2012**, *14* (3), 752–755. https://doi.org/10.1021/ol203298r.

(2) Pérez-Tejada, R.; Martínez de Baroja, N.; Franco, S.; Pellejà, L.; Orduna, J.; Andreu, R.; Garín, J. Organic Sensitizers Bearing a Trialkylsilyl Ether Group for Liquid Dye Sensitized Solar Cells. *Dyes and Pigments* **2015**, *123*, 293–303. https://doi.org/10.1016/j.dyepig.2015.07.026.

(3) Abate, A.; Pérez-Tejada, R.; Wojciechowski, K.; Foster, J. M.; Sadhanala, A.; Steiner, U.; Snaith, H. J.; Franco, S.; Orduna, J. Phosphonic Anchoring Groups in Organic Dyes for Solid-State Solar Cells. *Phys. Chem. Chem. Phys.* **2015**, *17* (28), 18780–18789. https://doi.org/10.1039/C5CP02671G.

(4) Andrés-Castán, J. M.; Andreu, R.; Villacampa, B.; Orduna, J.; Franco, S. 4*H*-Pyranylidene Organic Dyes for Dye-Sensitized Solar Cells: Twisted Structures towards Enhanced Power Conversion Efficiencies. *Solar Energy* **2019**, *193*, 74–84. https://doi.org/10.1016/j.solener.2019.09.028.

(5) Moreno-Yruela, C.; Garín, J.; Orduna, J.; Franco, S.; Quintero, E.; López Navarrete, J. T.; Diosdado, B. E.; Villacampa, B.; Casado, J.; Andreu, R. D−π−A Compounds with Tunable Intramolecular Charge Transfer Achieved by Incorporation of Butenolide Nitriles as Acceptor Moieties. *J. Org. Chem.* **2015**, *80* (24), 12115–12128. https://doi.org/10.1021/acs.joc.5b02051.

(6) Ford, J. A.; Wilson, C. V.; Young, W. R. The Preparation of 2(5H)-Furanones and Dyes Derived from Them. *J. Org. Chem.* **1967**, *32* (1), 173–177. https://doi.org/10.1021/jo01277a042.

(7) Lee, C.-H.; S. Lindsey, J. One-Flask Synthesis of *Meso*-Substituted Dipyrromethanes and Their Application in the Synthesis of *Trans*-Substituted Porphyrin Building Blocks. *Tetrahedron* **1994**, *50* (39), 11427–11440. https://doi.org/10.1016/S0040-4020(01)89282-6.

(8) Smith, M. J.; Blake, I. M.; Clegg, W.; Anderson, H. L. Push–Pull Quinoidal Porphyrins. *Org. Biomol. Chem.* **2018**, *16* (19), 3648–3654. https://doi.org/10.1039/C8OB00491A.

(9) Liu, Y.; Lin, H.; Dy, J. T.; Tamaki, K.; Nakazaki, J.; Nakayama, D.; Uchida, S.; Kubo, T.; Segawa, H. N-Fused Carbazole–Zinc Porphyrin–Free-Base Porphyrin Triad for Efficient near-IR Dye-Sensitized Solar Cells. *Chem. Commun.* **2011**, *47* (13), 4010–4012. https://doi.org/10.1039/C0CC03306E.

(10) Andrés-Castán, J. M.; Franco, S.; Villacampa, B.; Orduna, J.; Pérez-Tejada, R. New Efficient Tert-Butyldiphenyl-4H-Pyranylidene Sensitizers for DSSCs. *RSC Adv.* **2015**, *5* (129), 106706–106709. https://doi.org/10.1039/C5RA23339A.

(11) Galán, E.; Andreu, R.; Garín, J.; Mosteo, L.; Orduna, J.; Villacampa, B.; Diosdado, B. E. Influence of Thiazole Regioisomerism on Second-Order Nonlinear Optical Chromophores. *Tetrahedron* **2012**, *68* (32), 6427–6437. https://doi.org/10.1016/j.tet.2012.05.123.

(12) Marco, A. B.; Martínez de Baroja, N.; Franco, S.; Garín, J.; Orduna, J.; Villacampa, B.; Revuelto, A.; Andreu, R. Dithienopyrrole as a Rigid Alternative to the Bithiophene π Relay in Chromophores with Second-Order Nonlinear Optical Properties. *Chemistry An Asian Journal* **2015**, *10* (1), 188–197. https://doi.org/10.1002/asia.201402870.

(13) Vanallan, J. A.; Reynolds, G. A.; Petropoulos, C. C.; Maier, D. P. Reactions of Some 4-methylene-4 *H* -pyran Derivatives with Primary and Secondary Amines. *Journal of Heterocyclic Chem* **1970**, *7* (3), 495–507. https://doi.org/10.1002/jhet.5570070306.

(14) Tejeda-Orusco, V.; Blais, M.; Cabanetos, C.; Blanchard, P.; Andreu, R.; Franco, S.; Orduna, J.; Diosdado, B. E. 4H-Pyranylidene-Based Small Push-Pull Chromophores: Synthesis, Structure, Electronic Properties and Photovoltaic Evaluation. *Dyes and Pigments* **2020**, *178*, 108357. https://doi.org/10.1016/j.dyepig.2020.108357.

(15) Pérez Tejada, R.; Pellejà, L.; Palomares, E.; Franco, S.; Orduna, J.; Garín, J.; Andreu, R. Novel 4*H*-Pyranylidene Organic Dyes for Dye-Sensitized Solar Cells: Effect of Different Heteroaromatic Rings on the Photovoltaic Properties. *Organic Electronics* **2014**, *15* (11), 3237–3250. https://doi.org/10.1016/j.orgel.2014.09.003.

(16) Abaev, V; Karsanov, I; Urtaeva, Z; Blinokhvatov, A; Bumber, A. Production and Properties of (4H-Pyran-4-Yl)Diphenylphosphine Oxides. *Zh Obshch Khim* **1990**, *60*, 1012–1019.

(17) Andreu, R.; Galán, E.; Garín, J.; Herrero, V.; Lacarra, E.; Orduna, J.; Alicante, R.; Villacampa, B. Linear and V-Shaped Nonlinear Optical Chromophores with Multiple 4H-Pyran-4-Ylidene Moieties. *J. Org. Chem.* **2010**, *75* (5), 1684–1692. https://doi.org/10.1021/jo902670z.

(18) Erdmann, D. D.-C. D. 6101 T. S. K. D.-C. D. 6101 N.-R. K. W. D. 6100 D.-A. S. G. D.-B. 6100 D. C. 5-04. Process for the Preparation of 3,4-Disubstituted "(5H) - Furanones. DE2116416A1, October 19, 1972. https://patents.google.com/patent/DE2116416A1/en (accessed 2024-09-12).

(19) Holliday, S.; Ashraf, R. S.; Nielsen, C. B.; Kirkus, M.; Röhr, J. A.; Tan, C.-H.; Collado-Fregoso, E.; Knall, A.-C.; Durrant, J. R.; Nelson, J.; McCulloch, I. A Rhodanine Flanked Nonfullerene Acceptor for Solution-Processed Organic Photovoltaics. *J Am Chem Soc* **2015**, *137* (2), 898–904. https://doi.org/10.1021/ja5110602.

(20) Posner, G. H.; Li, Z.; White, M. C.; Vinader, V.; Takeuchi, K.; Guggino, S. E.; Dolan, P.; Kensler, T. W. 1 Alpha,25-Dihydroxyvitamin D3 Analogs Featuring Aromatic and Heteroaromatic Rings: Design, Synthesis, and Preliminary Biological Testing. *J Med Chem* **1995**, *38* (22), 4529–4537. https://doi.org/10.1021/jm00022a019.

(21) Reynolds, G. A.; Chen, C. H. Some Reactions of 4 *H* -pyrylium Salts with Tributylphosphine and with Tertiary Amines. *Journal of Heterocyclic Chem* **1981**, *18* (6), 1235–1237. https://doi.org/10.1002/jhet.5570180632.

(22) Ermer, S.; Lovejoy, S. m.; Bedworth, P. v.; Leung, D. s.; Warren, H. b.; Epstein, J. a.; Girton, D. g.; Dries, L. s.; Taylor, R. e.; Barto Jr., R. r.; Eades, W.; Van Eck, T. e.; Moss, A. s.; Anderson, W. w. Low-Voltage Electro-Optic Modulation Using Amorphous Polycarbonate Host Material. *Advanced Functional Materials* **2002**, *12* (9), 605–610. https://doi.org/10.1002/1616-3028(20020916)12:9<605::AID-ADFM605>3.0.CO;2-S.

(23) Nagahora, N.; Tokumaru, H.; Ikaga, S.; Hanada, T.; Shioji, K.; Okuma, K. Synthetic Studies on a Series of Functionalized Pyrylium Salts, 4-Chloro- and 4-Bromophosphinines. *Tetrahedron* **2018**, *74* (15), 1880–1887. https://doi.org/10.1016/j.tet.2018.02.053.

(24) Abdelrazek, F. M.; Michael, F. A. New Data about the Reaction of Benzyolacetonitrile with Malononitrile and Its Self-condensation. *Journal of Heterocyclic Chem* **2006**, *43* (1), 7–10. https://doi.org/10.1002/jhet.5570430102.

(25) Marco, A. B.; Andreu, R.; Franco, S.; Garín, J.; Orduna, J.; Villacampa, B.; Alicante, R. Efficient Second-Order Nonlinear Optical Chromophores Based on Dithienothiophene and Thienothiophene Bridges. *Tetrahedron* **2013**, *69* (19), 3919–3926. https://doi.org/10.1016/j.tet.2013.03.027.

(26) Andreu, R.; Galán, E.; Orduna, J.; Villacampa, B.; Alicante, R.; Navarrete, J. T. L.; Casado, J.; Garín, J. Aromatic/Proaromatic Donors in 2-Dicyanomethylenethiazole Merocyanines: From Neutral to Strongly Zwitterionic Nonlinear Optical Chromophores. *Chemistry – A European Journal* **2011**, *17* (3), 826–838. https://doi.org/10.1002/chem.201002158.
