# Supplementary material for: New Chemical Scaffold with Antimicrobial Activity Identified in a Screening of Industrial Photoactive Compounds
Source: Antibiotics (Basel). 2026 Mar 20;15(3):321. doi: 10.3390/antibiotics15030321 (PMC13024089; doi:10.3390/antibiotics15030321)
Supplement: Supplementary file 1 [file antibiotics-15-00321-s001.zip › Supp/Table S6 UV spectra.pdf]

Table S6. UV spectra of the compounds used in this study

Reference	UV spectrum
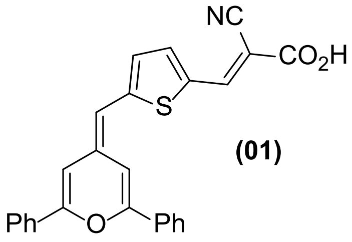 **(01)**	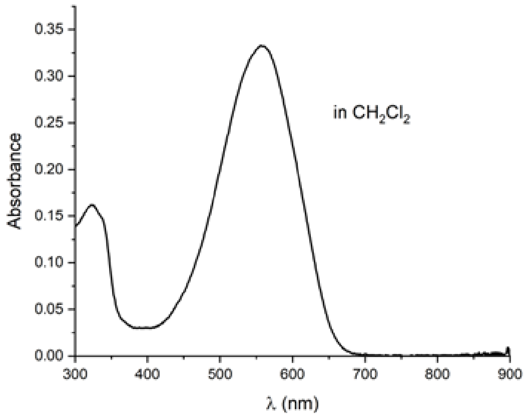
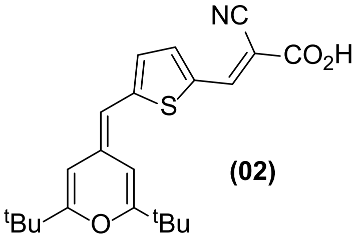 **(02)**	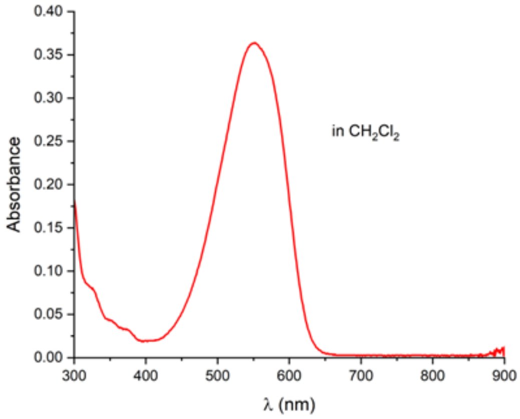
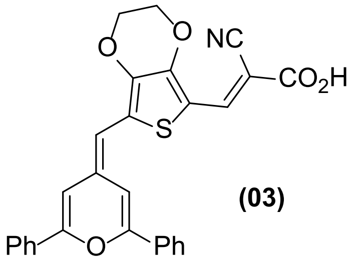 **(03)**	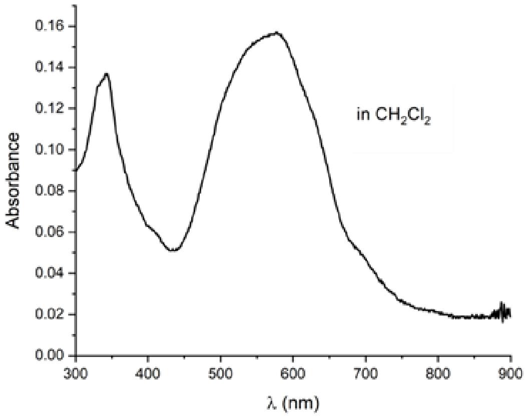
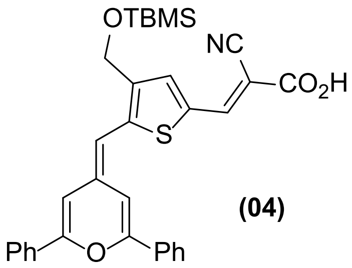 **(04)**	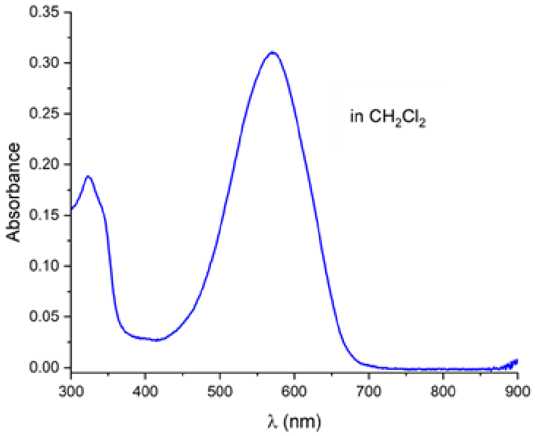

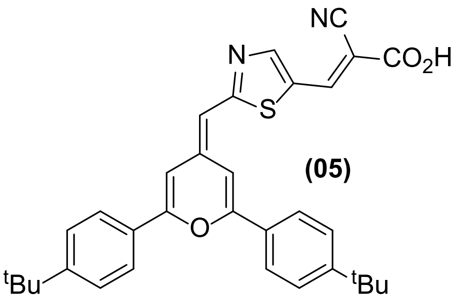 **(05)**	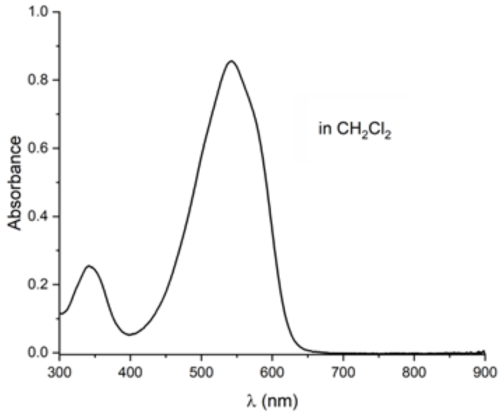Absorbance<br>λ (nm)<br>in CH_2Cl_2
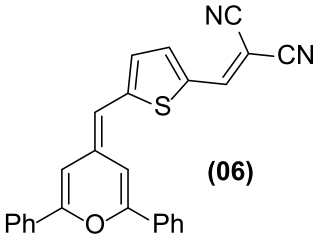 **(06)**	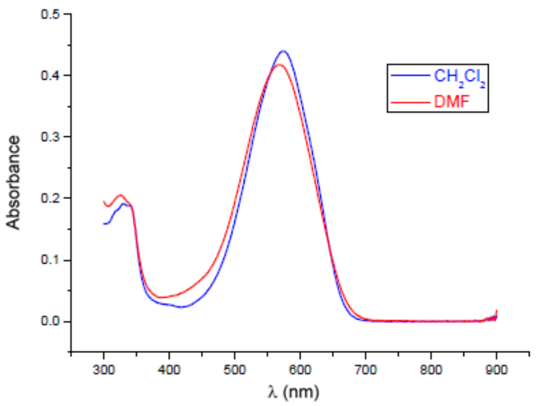Absorbance<br>λ (nm)<br>CH_2Cl_2<br>DMF
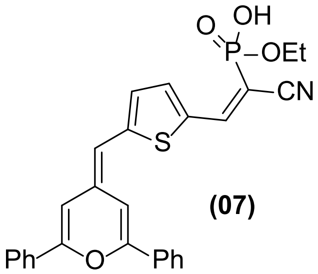 **(07)**	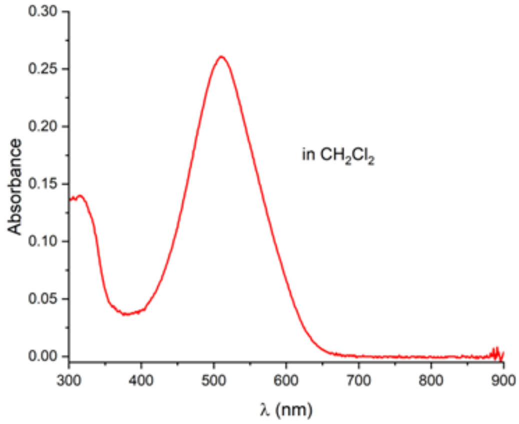Absorbance<br>λ (nm)<br>in CH_2Cl_2
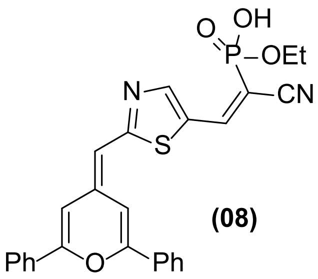 **(08)**	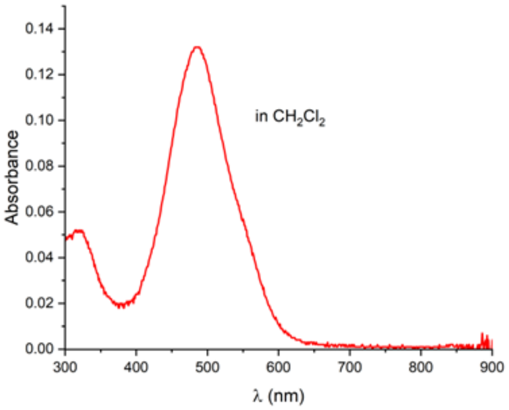Absorbance<br>λ (nm)<br>in CH_2Cl_2

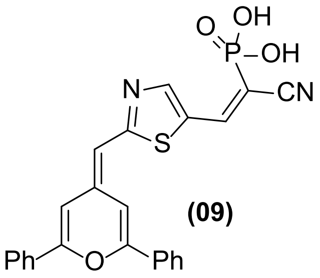 **(09)**	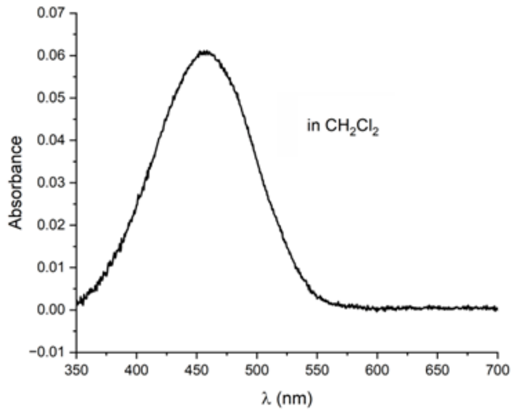 Absorbance<br>λ (nm)<br>in CH_2Cl_2
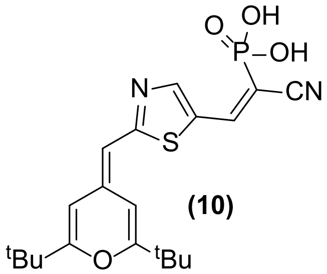 **(10)**	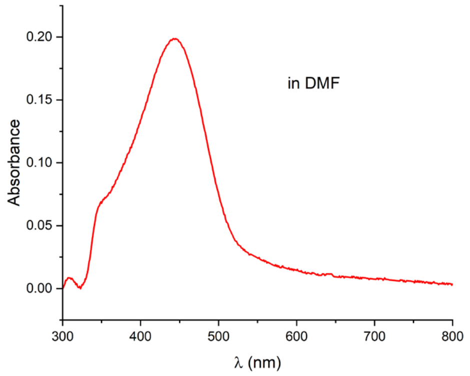 Absorbance<br>λ (nm)<br>in DMF
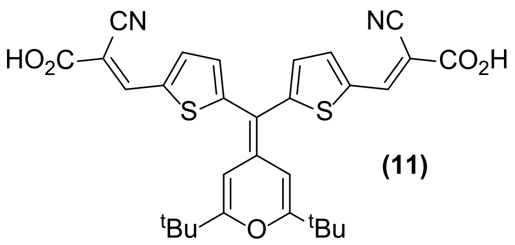 **(11)**	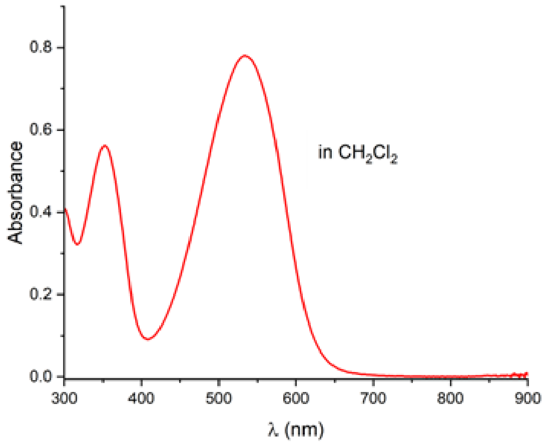 Absorbance<br>λ (nm)<br>in CH_2Cl_2
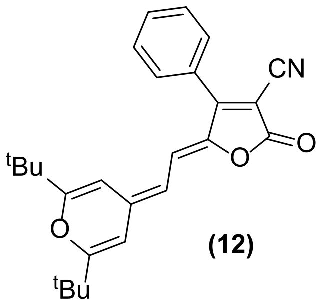 **(12)**	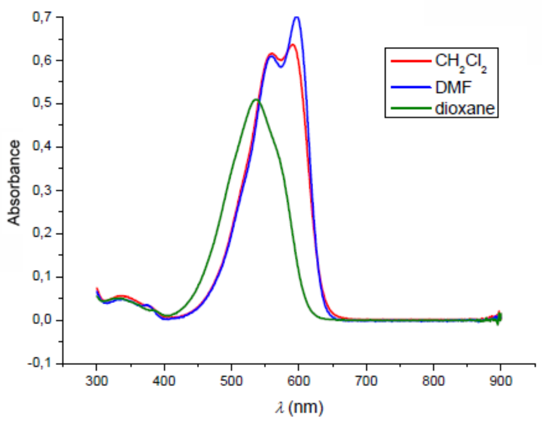 Absorbance<br>λ (nm)<br>CH_2Cl_2<br>DMF<br>dioxane

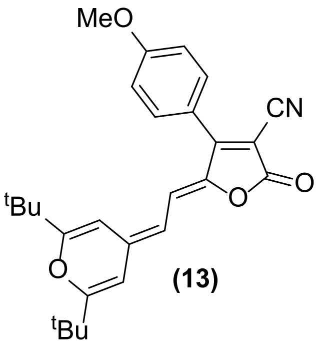

(13)

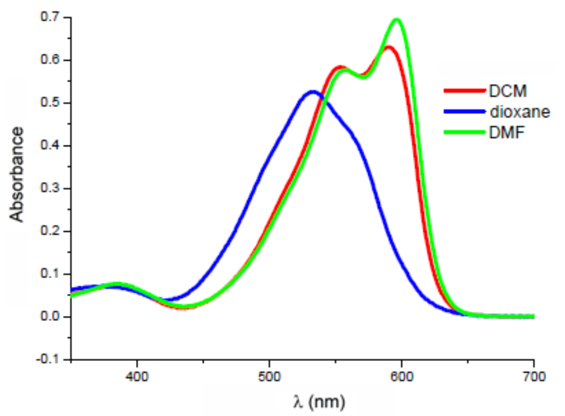
Absorbance
λ (nm)
DCM
dioxane
DMF

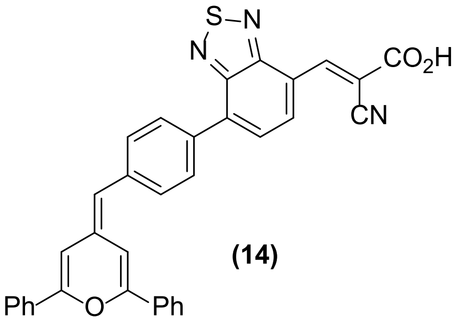

(14)

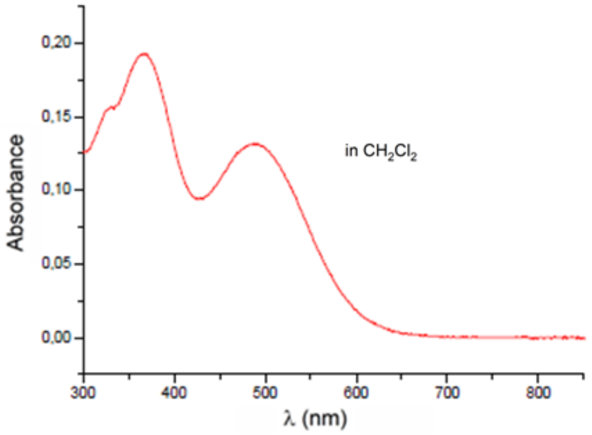
Absorbance
λ (nm)
in CH_2Cl_2

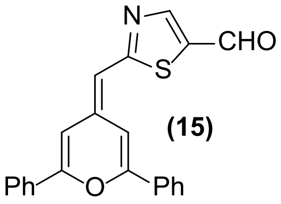

(15)

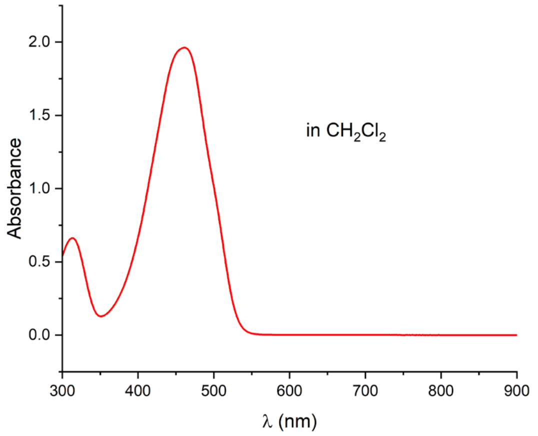
Absorbance
λ (nm)
in CH_2Cl_2

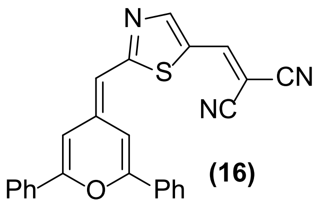

(16)

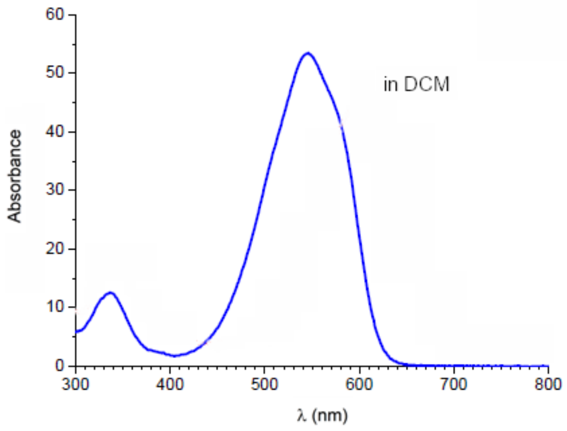
Absorbance
λ (nm)
in DCM

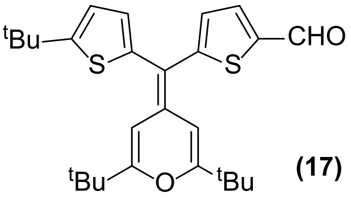 **(17)**	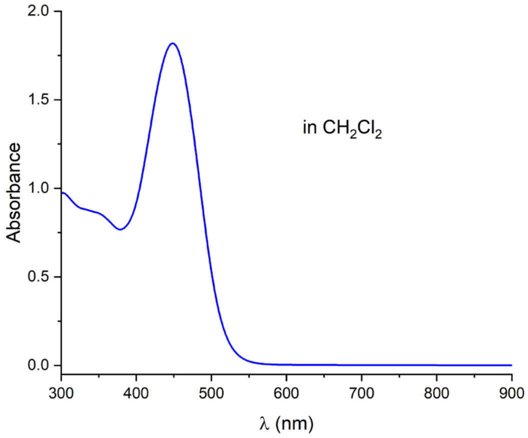Absorbance<br>λ (nm)<br>in CH2Cl2
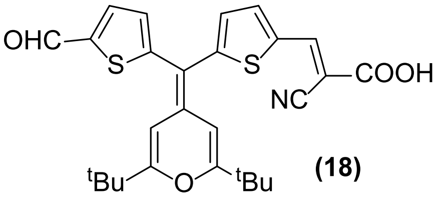 **(18)**	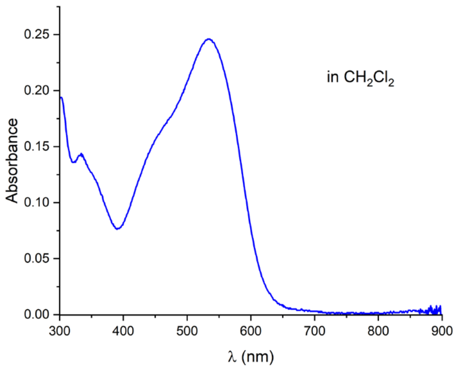Absorbance<br>λ (nm)<br>in CH2Cl2
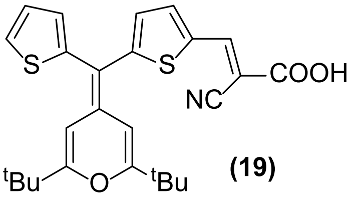 **(19)**	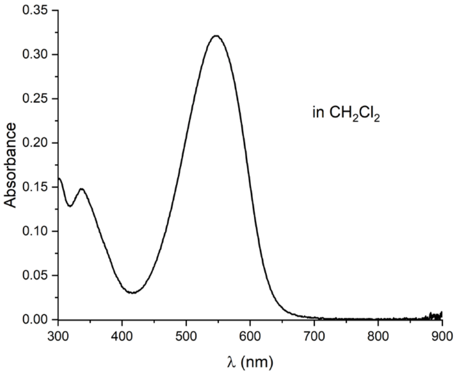Absorbance<br>λ (nm)<br>in CH2Cl2
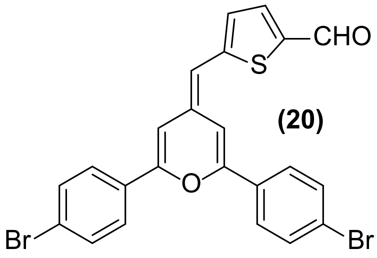 **(20)**	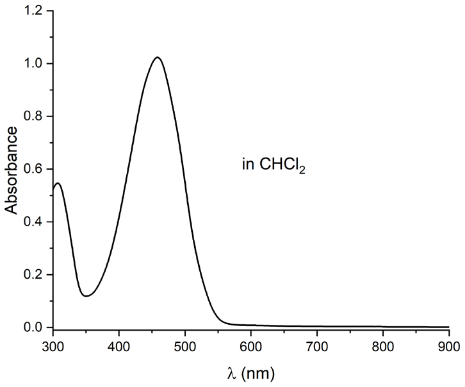Absorbance<br>λ (nm)<br>in CHCl2

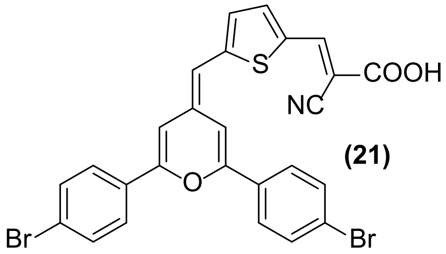S<br>COOH<br>NC<br>O<br>Br<br>Br **(21)**	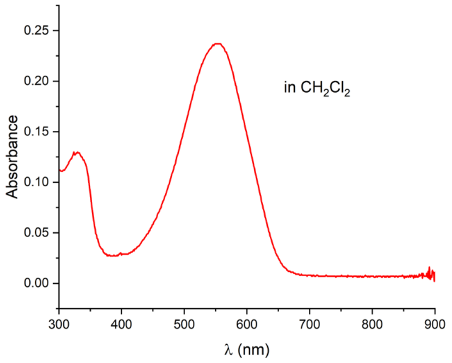Absorbance<br>λ (nm)<br>in CH_2Cl_2
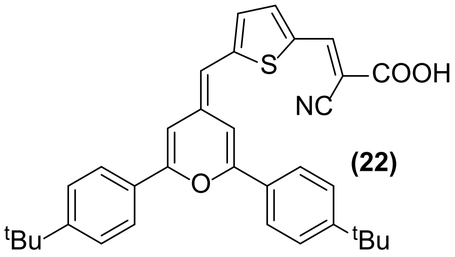S<br>COOH<br>NC<br>O<br>^{t}Bu<br>^{t}Bu **(22)**	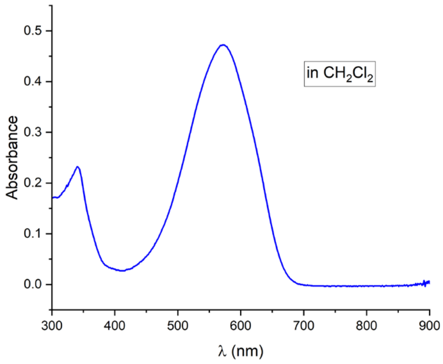Absorbance<br>λ (nm)<br>in CH_2Cl_2
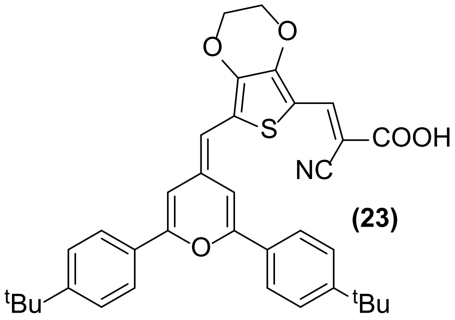O<br>O<br>S<br>COOH<br>NC<br>O<br>^{t}Bu<br>^{t}Bu **(23)**	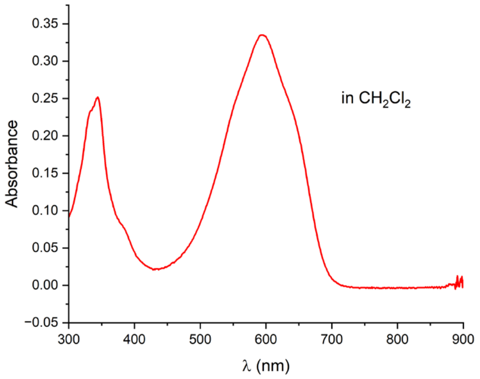Absorbance<br>λ (nm)<br>in CH_2Cl_2
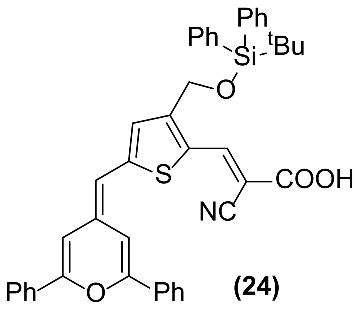Ph<br>Ph<br>Si<br>^{t}Bu<br>O<br>S<br>COOH<br>NC<br>Ph<br>O<br>Ph **(24)**	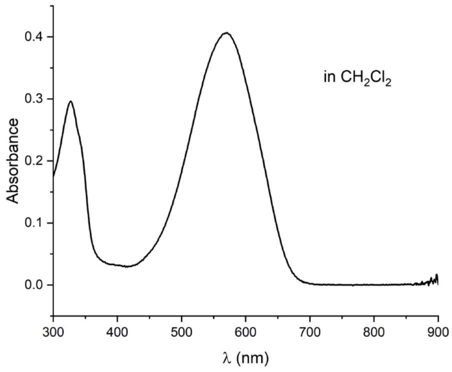Absorbance<br>λ (nm)<br>in CH_2Cl_2

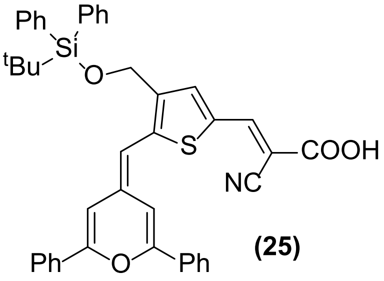
Ph
Ph
Si
tBu
O
S
COOH
NC
Ph
O
Ph

(25)

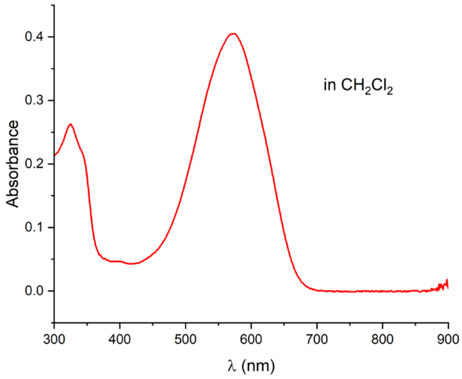
in CH2Cl2
Absorbance
λ (nm)
0.0
0.1
0.2
0.3
0.4
300
400
500
600
700
800
900

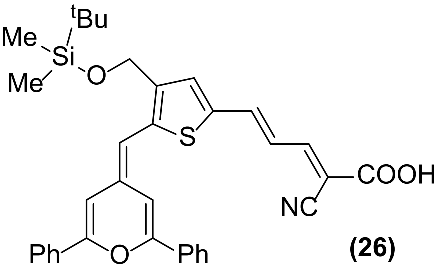
tBu
Me
Si
Me
O
S
COOH
NC
Ph
O
Ph

(26)

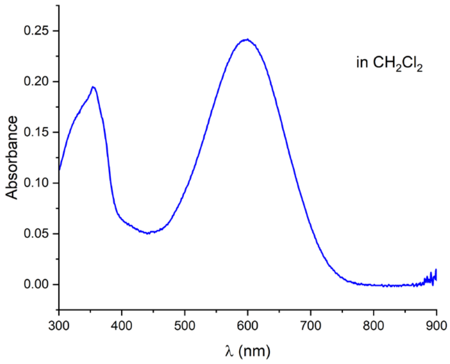
in CH2Cl2
Absorbance
λ (nm)
0.00
0.05
0.10
0.15
0.20
0.25
300
400
500
600
700
800
900

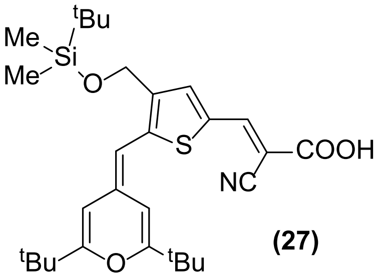
tBu
Me
Si
Me
O
S
COOH
NC
tBu
O
tBu

(27)

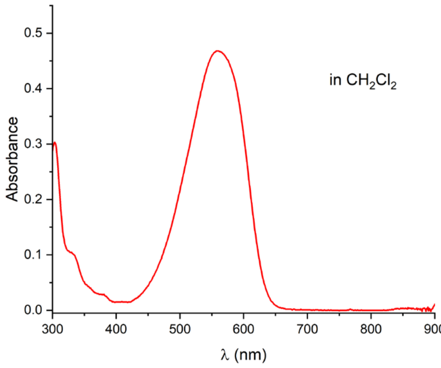
in CH2Cl2
Absorbance
λ (nm)
0.0
0.1
0.2
0.3
0.4
0.5
300
400
500
600
700
800
900

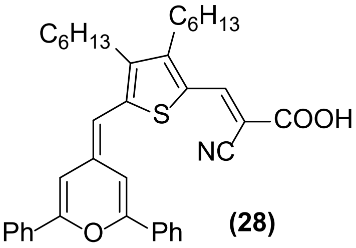
C6H13
C6H13
S
COOH
NC
Ph
O
Ph

(28)

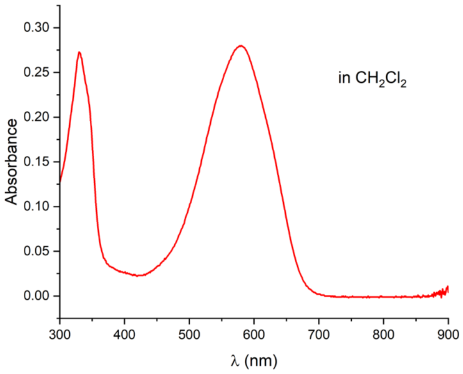
in CH2Cl2
Absorbance
λ (nm)
0.00
0.05
0.10
0.15
0.20
0.25
0.30
300
400
500
600
700
800
900

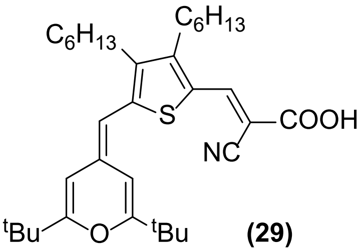 **(29)**	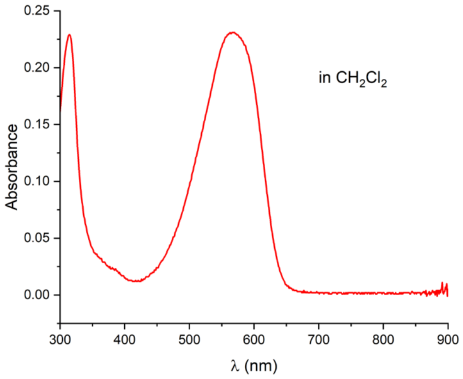
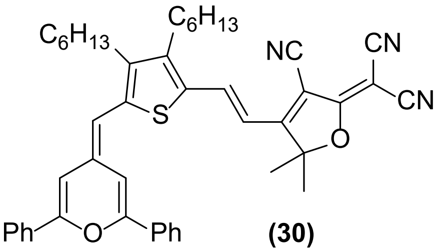 **(30)**	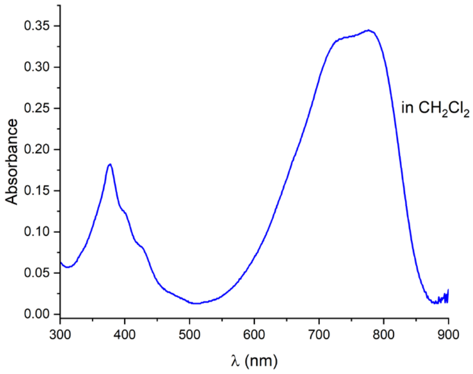
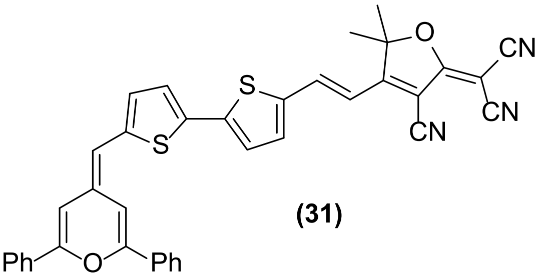 **(31)**	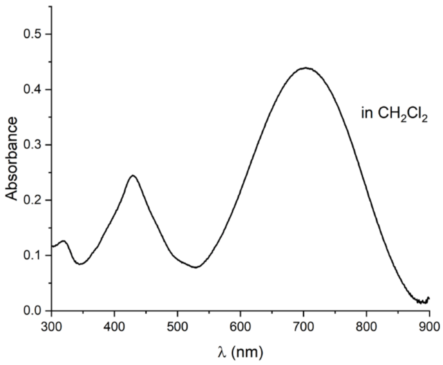
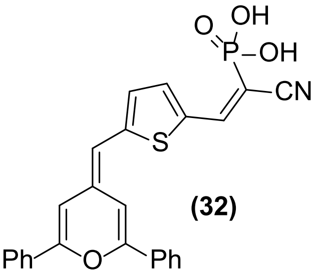 **(32)**	Not available

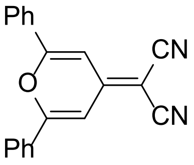

(33)

Not available

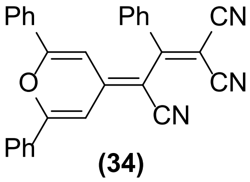

(34)

Not available

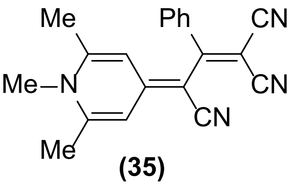

(35)

Not available

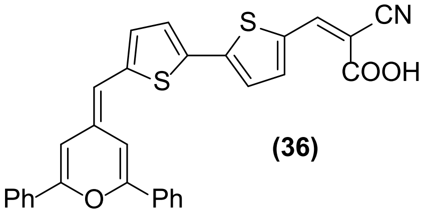

(36)

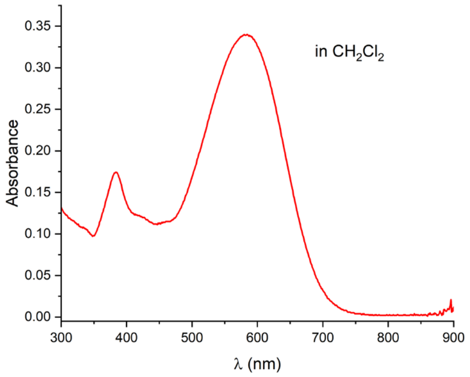

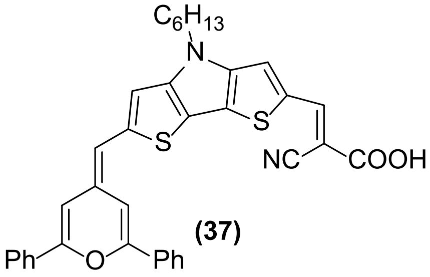

(37)

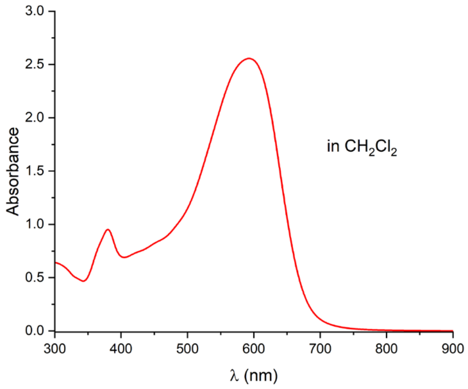

Absorbance
λ (nm)
in CH2Cl2

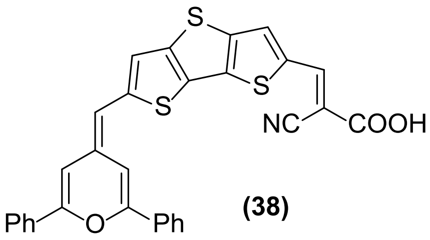

(38)

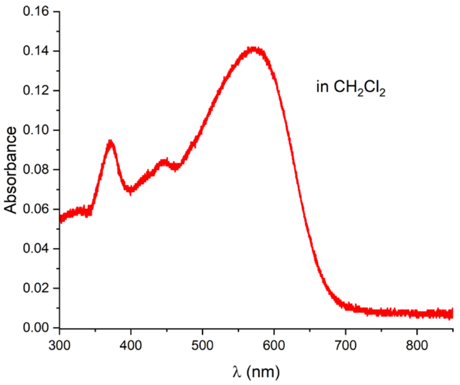

Absorbance
λ (nm)
in CH2Cl2

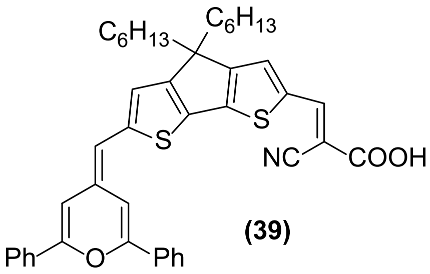

(39)

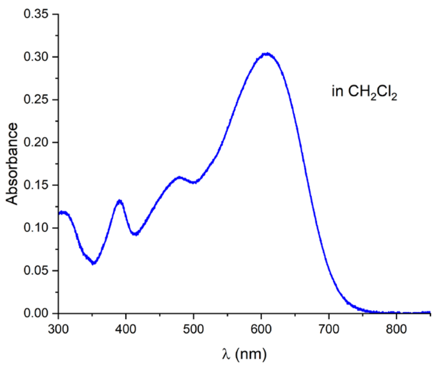

Absorbance
λ (nm)
in CH2Cl2

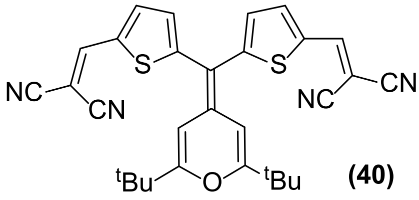

(40)

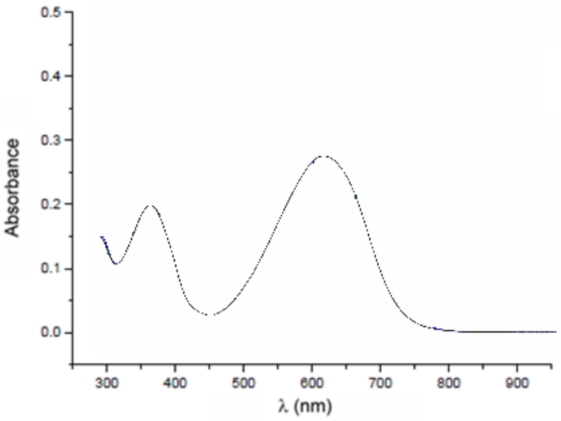

Absorbance
λ (nm)

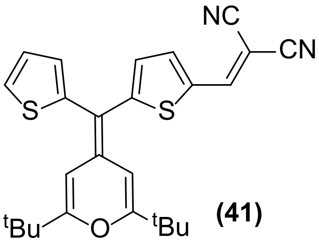 NC<br>CN<br>S<br>S<br>tBu<br>O<br>tBu **(41)**	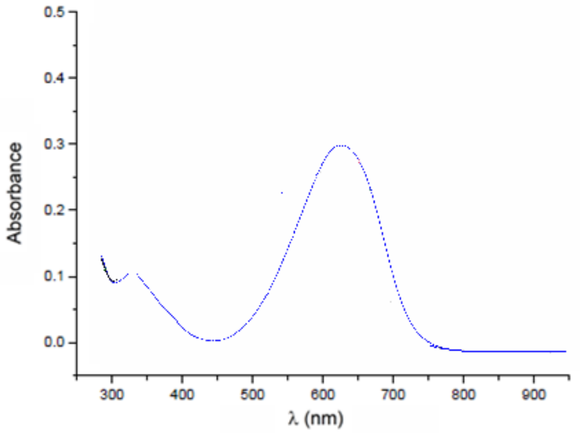 Absorbance<br>λ (nm)
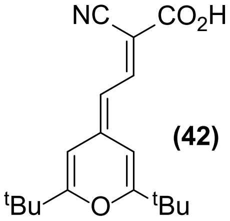 NC<br>CO2H<br>tBu<br>O<br>tBu **(42)**	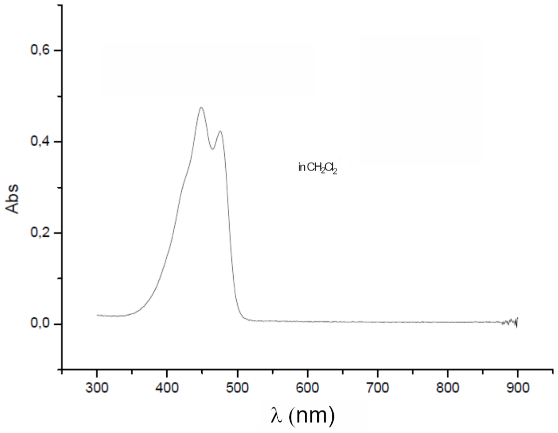 Abs<br>in CH2Cl2<br>λ (nm)
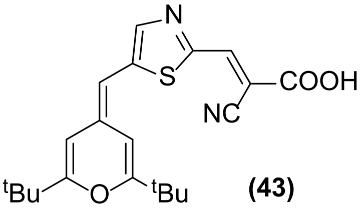 N<br>S<br>COOH<br>NC<br>tBu<br>O<br>tBu **(43)**	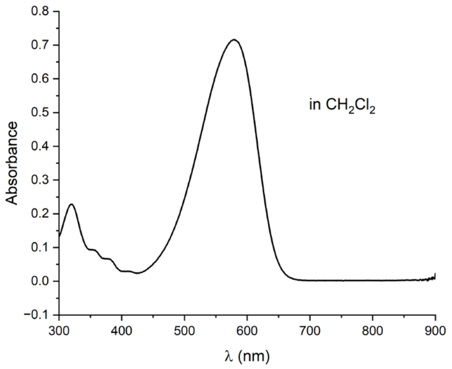 Absorbance<br>in CH2Cl2<br>λ (nm)
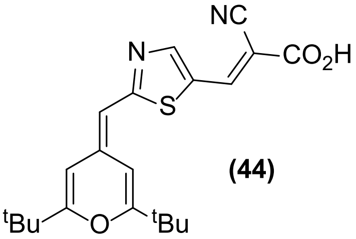 NC<br>N<br>CO2H<br>S<br>tBu<br>O<br>tBu **(44)**	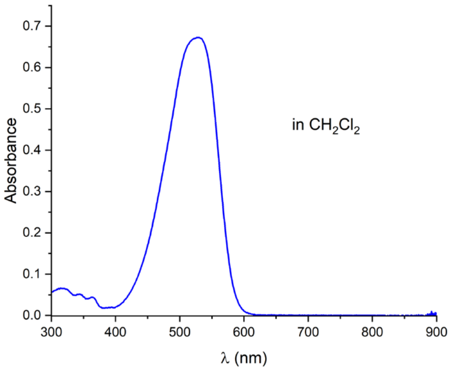 Absorbance<br>in CH2Cl2<br>λ (nm)

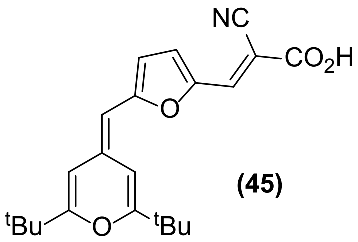
NC
CO2H
O
tBu
tBu
O

(45)

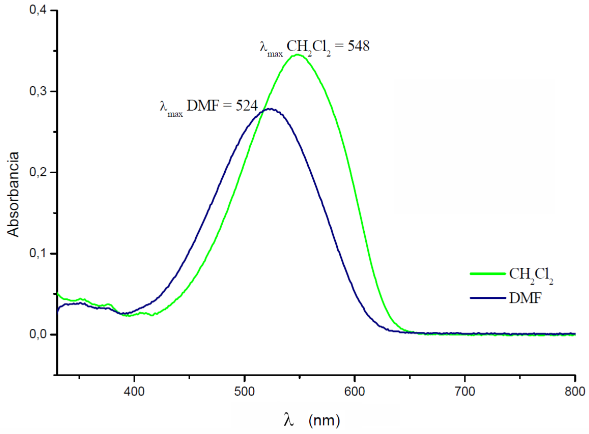
λmax CH2Cl2 = 548
λmax DMF = 524
Absorbancia
λ (nm)
CH2Cl2
DMF
0,0
0,1
0,2
0,3
0,4
400
500
600
700
800

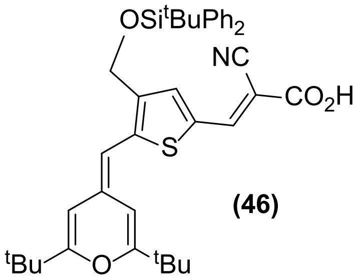
OSitBuPh2
NC
CO2H
S
tBu
O
tBu

(46)

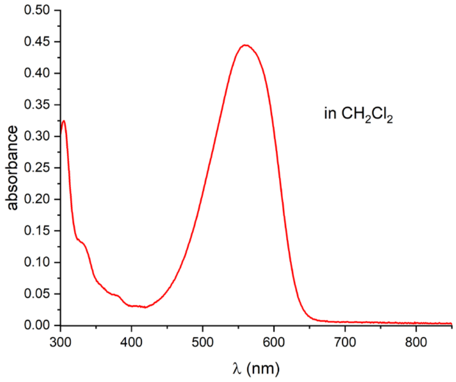
in CH2Cl2
absorbance
λ (nm)
0.00
0.05
0.10
0.15
0.20
0.25
0.30
0.35
0.40
0.45
0.50
300
400
500
600
700
800

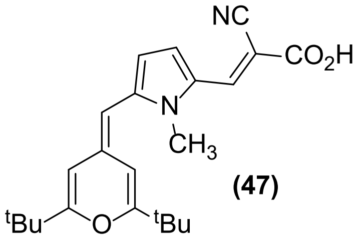
NC
CO2H
N
CH3
tBu
O
tBu

(47)

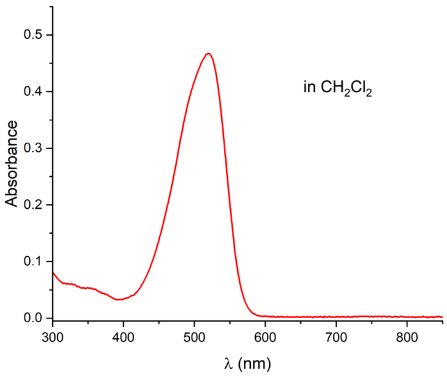
in CH2Cl2
Absorbance
λ (nm)
0.0
0.1
0.2
0.3
0.4
0.5
300
400
500
600
700
800
